# Primer PICKR: literature-mined scoring platform for robust RT–qPCR primers

**DOI:** 10.1038/s41467-026-73648-2

**Published:** 2026-05-28

**Authors:** Thomas G. Molley, Abhinaba Banerjee, Alis Balayan, Jun K. Robbins, Adam J. Engler

**Affiliations:** 1 Shu Chien-Gene Lay Department of Bioengineering, University of California, San Diego, La Jolla, CA USA; 2 Sanford Consortium for Regenerative Medicine, La Jolla, CA USA; 3 Biomedical Sciences Program, University of California, San Diego, La Jolla, CA USA

**Keywords:** PCR-based techniques, Reverse transcription polymerase chain reaction, Synthetic biology

## Abstract

Reliable reverse-transcription quantitative PCR (RT–qPCR) depends on well-designed primers, yet undocumented or poorly validated sequences continue to compromise reproducibility. Existing resources catalog only modest sets of empirically verified primers or generate de-novo primer pairs without experimental validation. Here, we introduce *Primer PICKR* (Publication Integrations for Composite Knowledge Ranking), a large-scale, open, continuously updated database that systematically converts >7,000,000 community-validated oligonucleotides from >400,000 papers into actionable design resources. PICKR aligns sequences to reference transcriptomes and assembles ranked primer pairs for over 6000 genes across ten model organisms. Composite scoring integrates citation frequency, biophysical quality, and primer-pair synergy, while experimental validation of 154 human primer pairs spanning the score distribution demonstrates near-perfect amplification success above a PICKR score of 80. By converting decades of scattered primer choices into an immediately searchable database, Primer PICKR reduces empirical screening, conserves scarce samples, and accelerates reproducible assay development.

## Abstract (web summary)

Reverse transcription PCR is widely used but often suffers from unreliable primer selection. Here, the authors introduce Primer PICKR, a database that ranks primers using literature data, achieving near-perfect amplification success for high-scoring pairs and improving reproducibility.

## Introduction

Reverse transcription quantitative polymerase chain reaction (RT–qPCR) remains one of the most widely adopted tools to quantify gene expression, referenced in over 1.5 million publications in the last three decades. Despite its ubiquity, RT–qPCR remains uniquely vulnerable to a persistent pain point: primer selection. Over three decades of RT–qPCR use has led to consensus-based primer design rules, including the ideal melting temperature (T_m_ ≈ 60 °C), balanced GC content (40–60%), amplicons <200 bp, negligible self- or cross-complementarity, and strict avoidance of genomic repeats^1–5^. Even when primers conform to these rules, many users encounter off-target amplification, low reaction efficiencies, or failed amplification. Crucially, even a single base-pair mismatch can distort cycle-threshold (CT) values, and silent cross-reactivity to pseudogenes or orthologues can inflate apparent expression, compromising reproducibility and rigor.

Multiple strategies implemented over a decade ago attempted to eliminate these methodological challenges with varied success. Decades-old tools, e.g., Primer3^6–8^ and Primer-BLAST^9^, automate the in silico design of primer pairs yet return long un-ranked lists of candidates, sometimes with poor amplification efficiency. PrimerBank^10–12^ adds manual curation, but its ~130,000 human primers are optimized for conventional PCR and lack primer metadata, including complementarities and off-target specificity. Commercial probe collections, e.g., TaqMan, offer proprietary validation but come with additional costs and limited flexibility for unconventional targets. Thus, researchers routinely purchase multiple primer pairs per gene to identify those that amplify, inflating reagent use, consuming valuable samples, and increasing operator time significantly.

Evidence of this problem is apparent in our survey of PubMed Central (PMC), which reveals that 31% of all oligo sequences found are unique and 56% of sequences appear in fewer than ten publications; these data underscore how fragmented the primer landscape is. Even for common housekeeping genes, hundreds to thousands of distinct primer sequences exist, rather than the adoption of empirically validated designs. In this respect, primer choice lags far behind antibody selection, where services such as CiteAb^13^ use crowd-sourced performance data to guide purchasing suggestions; an equivalent, community-validated tool for RT–qPCR primers is absent.

**Fig. 1:**
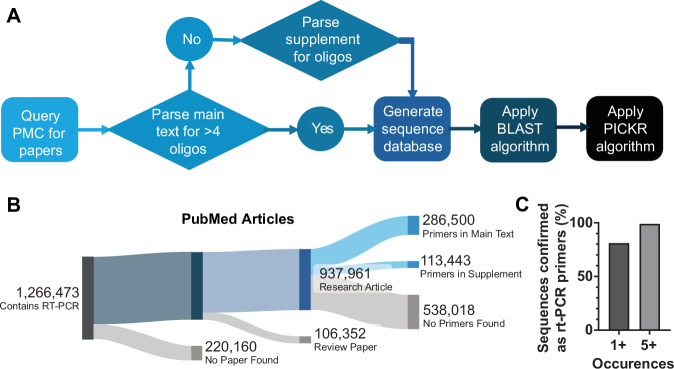
Primer capture pipeline. **A** Schematic of the PICKR workflow used to scrape and score primer pairs. **B** Sankey diagram of the breakdown of papers used to scrap primers. **C** Quantification of manually validated sequences that were RT–qPCR primers. Source data are provided as a Source Data file.

To address this fundamental gap, we introduce Primer PICKR (Publication Integrations for Composite Knowledge Ranking), a literature-mined, continuously updated and open-access database that converts global RT–qPCR experience into actionable primer recommendations (Fig. 1A). By mining ~400,000 open-access manuscripts from Europe PMC (Fig. 1B), we extract 7.1 million nucleotide strings, filter, and BLAST-map 290,191 unique primers to 6647 genes across ten species, including >5,000 human loci^14,15^. For every gene, we generated forward-reverse combinations and computed a composite “PICKR score” that integrates literature evidence, individual primer biophysics, and primer-pair synergy. We demonstrate the predictive power of the PICKR score through experimental validation of 154 primer pairs spanning five score deciles. High-scoring pairs ( > 80) yield 99 % amplification success while lower scoring pairs (50–60) show reduced performance (85% success rate). Finally, Primer PICKR is self-improving: through monthly updates, it integrates new publications and re-weights scoring metrics, allowing primer pairs that consistently perform well in the community to rise in rank while ineffective pairs sink. To make Primer PICKR widely accessible, we present an intuitive web interface (www.primerpickr.com) that returns ranked pairs in milliseconds, with direct PubMed links, pre-computed BLAST cross-checks, one-click sequence copying, and species-specific filters. Ultimately, Primer PICKR aims to save researchers’ time while elevating the quality and reproducibility of RT–qPCR experiments with a single, simple-to-use primer database.

## Results

### Building a primer corpus from publishing history

To mine primer sequences efficiently, we used only the Europe PMC as it contains the highest density of full-text, freely accessible articles ( > 20 million). A bespoke parser identified strings ≤40 nt composed solely of A, T, C, and G; sequences spanning table line-breaks were re-joined, and only articles listing ≥4 such strings were retained, a heuristic that captures forward, reverse, and housekeeping primers. About 1.2 million open-access life-science articles mentioned “RT–qPCR” and its derivatives in Methods, yet only 399,000 had ≥4 nucleotide strings. The primary loss stemmed from diagnostic COVID-19 reports that reference RT–PCR kits without listing sequences, plus older manuscripts that relegated primer details to pay-walled supplementary PDFs. However, the current pipeline yielded 8.1 million candidate oligos. Manual inspection of 100 randomly selected primers with ≥5 occurrences showed that 99% were bona-fide RT–qPCR primers, whereas the full set (no occurrence filter) contained ~20% non-RT–qPCR sequences such as CRISPR sgRNAs, shRNAs, and miRNAs (Fig. 1C).

### Global primer design trends

**Fig. 2:**
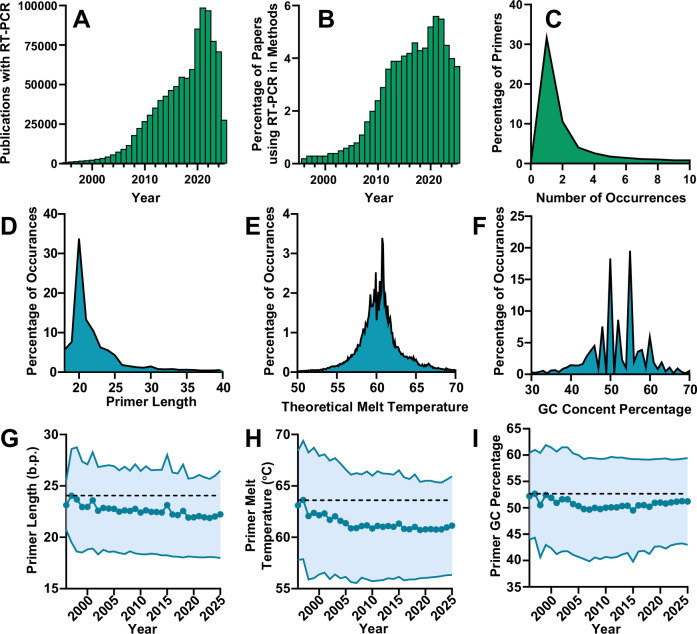
Primer statistics from scraped database. Histograms of total (**A**) and percentage (**B**) of papers published containing RT–qPCR in their methods. **C** Frequency plot of sequence occurrence. Percentage of occurrences plotted against primer length (**D**), theoretical melt temperature (**E**), and GC content (**F**). Changes per year in mean primer length (**G**), theoretical melt temperature (**H**) and GC content (**I**). All graphs were generated from 399,493 papers accessed through the Europe PMC database. Source data are provided as a Source Data file. The code used in the creation of these data is published under a CC-BY-NC-ND license (https://creativecommons.org/licenses/by-nc-nd/4.0/deed.en).

Of the 8.1 million oligo sequences, ~46% were published after 2015 and 28% after 2020 (Fig. 2A). The 2020–2022 spike corresponds to SARS-CoV-2 diagnostics. Interestingly, the share of research papers using RT–qPCR as a method has stabilized at ~4% since 2012, suggesting widespread adoption and accessibility across the community (Fig. 2B). However, literature analysis reveals striking fragmentation of assay design: 31% of all unique primer sequences appear in only a single publication, and 56% are cited 10 times or fewer (Fig. 2C). This heterogeneity implies that even for well-studied genes, most laboratories still design fresh primers instead of adopting pairs that the community has validated, prolonging optimization cycles and eroding reproducibility. *GAPDH* alone harbored ~10,000 primer variants, an illustration of redundant design efforts of primer pairs over the last several decades. For primers reused ≥5 times (to ensure sequences are RT–qPCR primers), >90% measured 18–22 nucleotides, 89% had 40–60% GC, and theoretical T_m_ followed a near-Gaussian distribution centered at 60.3 ± 1.7 °C (Fig. 2D–F). Over time, mean primer length and theoretical Tm remained relatively stable after approximately 2000, while GC content has remained remarkably constant across the same period (Fig. 2G–I).

### Species mapping and cross-specificity

**Fig. 3:**
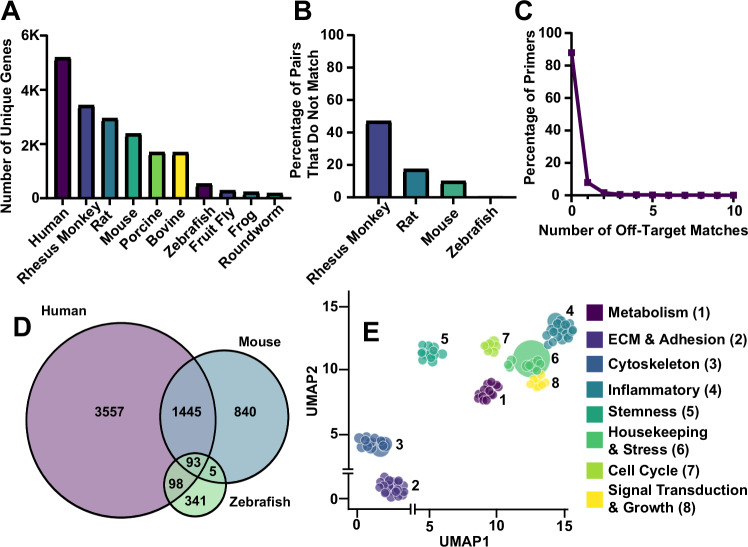
Mapped primers to species. **A** Number of unique genes found per species. **B** Percentage of species overlap for all human primers. **C** Number of off-target hits for human primers. **D** Venn diagram of gene matches between human, mouse, and zebrafish. **E** UMAP projection of top 100 genes clustered via GO term analysis with bubble size correlating to gene occurrences. All graphs were generated from the 290,071 unique primers used at least 3 times in the literature from the 399,394 papers scraped. Numbers correspond to the indicated clusters. Source data are provided as a Source Data file. The code used in the creation of these data is published under a CC-BY-NC-ND license (https://creativecommons.org/licenses/by-nc-nd/4.0/deed.en).

Using sequences with multiple occurrences greatly reduced our false positive rate (Fig. 1C). Therefore, we aggregated oligo sequences with ≥3 occurrences, leading to 290,191 unique RT–qPCR primers (53% of the 7,170,000 oligos). These primers were then aligned against RefSeq-RNA for human, rhesus macaque, mouse, rat, pig, cow, frog (*Xenopus laevis*), zebrafish, roundworms (*Caenorhabditis elegans*), and fruit flies (*Drosophila melanogaster*). To reduce the false positive rate to near 0, primers had to match exactly one transcript with 0 mismatched base pairs for each species to be added to species-specific gene lists. Gene coverage was over 5000 for humans, with over 1500 for all mammals tested (Fig. 3A). Phylogenetically distant species were found to have fewer than 600 genes, with as few as 181 for *Caenorhabditis elegans*. High gene counts in mammals are likely not only driven only by model species usage but also by genetic similarity to humans. To validate this, we ran a species cross check of all human-matched primer sequences and found that 50% of human primers had perfect rhesus matches whereas only 11% and 1% cross-matched to mouse and zebrafish, respectively (Fig. 3B). Yet, zebrafish had >60% of genes that are uniquely zebrafish (Supplementary Fig. 1A), suggesting that real zebrafish primers were accessed from manuscripts. To assess primer specificity within human mRNA, each sequence was tested against all other mRNAs with a Hamming distance up to 4 as >4 mismatches is highly unlikely to cause amplification^16^. At ≥5 mismatches, predicted duplex stability drops by orders of magnitude (ΔΔG ≈ +5–10 kcal/mol → ~10³–10⁵-fold weaker binding at RT-qPCR temperatures), and the likelihood of a 3′-proximal mismatch becomes high, further suppressing extension. Here, 87% of all primers were found to have no cross-specificity (Fig. 3C), with around 6% of sequences having a mismatch of only one nucleotide (Supplementary Fig. 1B).

To assess housekeeping gene coverage, fifteen common genes were compared (Supplementary Data 1). *GAPDH* and *ACTB* accounted for 82% of housekeeping primer occurrences and 24% of all primer occurrences. The most cited human primer in the database was for *GAPDH* with 3182 occurrences, with the highest overall primer being a mouse U6 primer at 5892 occurrences. The *GAPDH* sequence matches the forward sequence of the top PrimerBank entry, confirming historical dependence on that database. Staggeringly, *GAPDH* has almost 9000 unique primer sequences found at least 5 times. Given that only 9% of all sequences occur >3 times, it suggests potentially many thousands of unique sequences for *GAPDH* alone. Beyond housekeepers, we also wanted to know what aspects of cell biology are measured the most by RT–qPCR. Thus, we selected the top 100 genes from the most-cited primers, conducted Gene Ontology enrichment, and plotted a UMAP embedding clustered by unsupervised k-means with bubble size relative to primer occurrences (Fig. 3E). Eight distinct domains emerged with furthest and tightest domains corresponding to cytoskeletal proteins and ECM & adhesion proteins. Clustering and domain types echo contemporary priorities in regenerative medicine and tumor immunology.

### The PICKR scoring algorithm

Within research papers, forward vs reverse orientation as well as 5’−3’ primer orientation were denoted using a wide variety of notations, leading our scraping algorithm to identify correct orientation with insufficient accuracy. Therefore, we generated primer pairings de-novo and back-mapped pairings to our raw reads to identify pairs with shared citations. Here, the top 30 cited sequences per gene had reverse complements generated and permuted such that pairs whose 3′ ends lay 50–350 bp apart on the same mRNA strand were kept. To create our PICKR score, we used the following weighted component function:

1 $${PICKR}\,{score}=100(0.5e+0.3b+0.2s)$$

These three components were chosen to jointly capture the reliability, molecular suitability, and compatibility of a primer pair. The Evidence score, *e*, represents the joint article count for the pair (log-scaled, capped at 25), plus individual citations and reverse complement citations (capped at 100 and 500, respectively). The Biophysics score, *b*, penalizes for |T_m_ – 60 °C | , |GC – 50%|, length > 24 nt, self-hairpins ≥ 6 bp, and off-target hits with ≤ 4 mismatches. Finally, the Synergy score, *s*, penalizes for ΔT_m > 0 °C (with a score of 0 for > 3 °C) and hetero-dimerization ≥ 5 bp. Any pair complementarity >6 or perfect cross-specificity match assigns PICKR = 0 to avoid reporting poor primers. Only the top 30 scored primer pairs for each gene were kept and scores of 0 were deleted. The full breakdown of the scoring algorithm and individual functions can be found in the supplementary text.

**Fig. 4:**
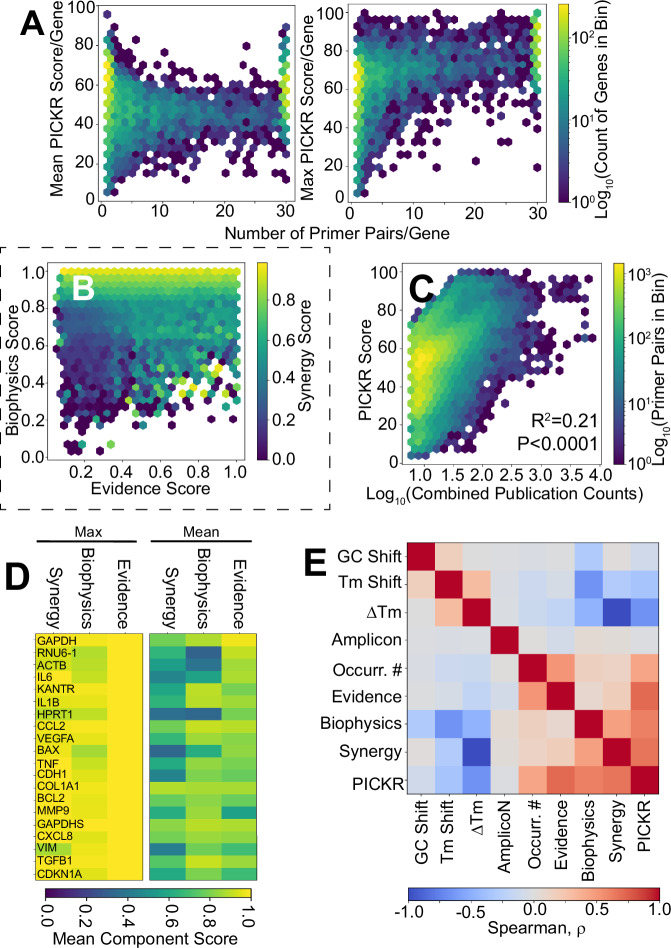
PICKR scores for human primers. **A** Hexbin plots of mean and maximum PICKR score for all human genes. **B** Hexbin plot of the three component scores (evidence, biophysics, and synergy). **C** Hexbin plot of PICKR score against log-transformed publication counts for primer pairs. **D** Component scores of the average and top primer pair for the twenty most used genes. **E** Pearson correlation plot of PICKR scores, the component scores, and the metadata used to calculate the scores. Sample sizes for all graphs were the 52,941 generated primer pairs derived from the 290,071 primers pulled from the literature. Source data are provided as a Source Data file. The code used to generate these data are published under a CC-BY-NC-ND license (https://creativecommons.org/licenses/by-nc-nd/4.0/deed.en).

Across 4.2 million evaluated pairs, the median PICKR score was 48 (interquartile range was 20-70), with a maximum score of 99.7. Genes with ≤10 viable pairings had lower median scores, reflecting limited empirical evidence, whereas heavily studied genes (30 pairings) invariably contained ≥1 high-scoring pair ( ≥ 80) (Fig. 4A). Correlation analysis revealed a strong coupling between biophysics and synergy scores, with a weaker coupling between evidence and synergy scores, driven by the fact that frequently reused primers often share similar T_m_ (Fig. 4B). This is seen clearly when plotting PICKR score against the log-transformed citation count for the pair (r^2^ = 0.206; *P* < 0.0001) (Fig. 4C). Plotting the top twenty most used genes revealed high evidence and synergy scores for best-ranked primers (Fig. 4D). Pearson correlation analysis revealed high correlations of the components of each subscore with the subscore itself (Fig. 4E). Interestingly, weak negative correlations were found between ΔT_m_ and the evidence score, suggesting that highly used primers typically had more similar melting temperatures, although we would have expected a stronger correlation to be found. Similar relations were found for mouse primers as well (Supplementary Fig. 2).

### Bench-top validation

**Fig. 5:**
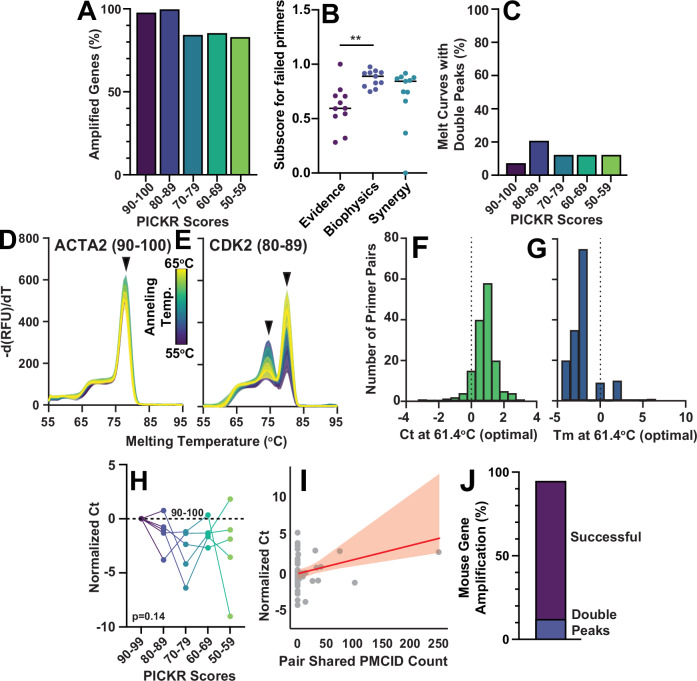
Experimental Validation of Primer PICKR scores. **A** Percentage of primers that amplified from each score bucket. **B** Plot of unweighted sub-scores, i.e., varying from 0 to 1, for failed primers indicating which sub-score is most sensitive to the failure. One-way ANOVA was used for statistical comparisons with **p = 0.0079. **C** Percentage of primer pairs with secondary peaks for at least 1 temperature. Example good (**D**) and bad (**E**) melt curves with peaks indicated by arrowheads. Histograms of the CT difference (**F**) and melt temperature difference (**G**) for all primer pairs at 61.4 °C compared to their most optimal melt temperature. **H** Difference in normalized CT of the 90-100 bucket as compared to all other buckets for the five genes with a primer pair from each bucket, i.e., *FN1*, *GAPDH, HPRT1, TBP*, and *VIM*. The nonparametric Friedman’s test with multiple comparisons was used for statistical analysis. **I** Scatter plot of PMCID versus CT. Red shading indicates the 95% confidence interval. **J** Amplification of 24 mouse primer pairs from >80 PICKR score. Sample sizes were: (**A**) *n* = 154, (**B**) *n* = 11 primers/score, (**C**) *n* = 144, (**F**) *n* = 132, (**G**) *n* = 132, (**H**) *n* = 5/score, (**I**) *n* = 115, and (**J**) is 24. Source data are provided as a Source Data file. The code used in the creation of these data are published under a CC-BY-NC-ND license (https://creativecommons.org/licenses/by-nc-nd/4.0/deed.en).

To explore how Primer PICKR primers perform in experimental validation, we selected 154 primer pairs stratified into PICKR score bins: 90–100, 80–90, 70–80, 60–70, and 50–60 targeting 94 unique transcripts in human lung fibroblasts. A detailed list of primers and their sequences can be found in Supplementary Data 2. We used a two-step cycling protocol with 95 °C 15 s for denaturation, 55-65 °C gradient 30 s for annealing/extension, repeated for 40 cycles followed by melting of the amplicon. Amplification success (defined as a CT value of <36 at any of the annealing temperatures) declined from 98% ( ≥ 90 score) to 83 % (50–60 score) (Fig. 5A**and** Supplementary Fig. 3-33). When a primer pair failed, another primer pair from a different scoring bin was tested. MMP3 and MMP9 were not expressed by human lung fibroblasts and were replaced as well. Critically, no primer pairs with at least one shared citation between the forward and reverse primers failed (62 pairs). Of the 11 primer pairs that failed, the median evidence score was 59, with biophysics and synergy median scores of 89 and 84, respectively (Fig. 5B). Together, this supports our algorithm weighting with evidence score accounting for half the PICKR score.

To detect whether the tested primers were specific, the derivative of each melt curve was analyzed for secondary peaks. We identified 24 primer pairs with secondary peaks, which represented 15% of all tested primers, where 90-100 bin primers had only ~9% (Fig. 5C-E, Supplementary Fig. 3, Supplementary Data 3). From the 24 primer pairs exhibiting secondary melt peaks, a subset of 12 reactions with visually distinct, high-amplitude secondary peaks were selected for amplicon purification and Sanger sequencing, and the resulting reads were aligned to the Homo sapiens reference genome using BLASTn to assess genomic specificity (Supplementary Data 4). In all cases, the dominant amplicon mapped uniquely to the intended target gene with high confidence (mean query coverage = 97%, mean percent identity = 99.6%, *p* values ranging from 10⁻³⁶ to 10⁻¹³⁹). No dominant off-target genomic loci were detected, indicating that secondary peaks likely reflect low-abundance artifacts (e.g., primer dimers or minor mis-primed species) rather than off-target amplification. However, one primer set, i.e., *TP53*_3, had no sequenceable amplicon and this case was excluded from specificity classification. Additionally, the 24 primer pairs with secondary peaks were tested without cDNA template to identify if they were due to primer dimer formation. The non-template controls for three pairs (*TP53_3*, *PDGFRb_1* and *CASP9_5*) showed peaks at low annealing temperatures (Supplementary Fig. 30-33). While the melting temperature curves of *PDGFRb_1* and *CASP9_5* had small peaks corresponding to secondary peaks in the experimental group, peak height was significantly lower than the threshold we set for classifying secondary peaks, i.e., 10% of all amplified primary peaks. Additionally, the recorded CT values were >38, indicating that even if primer dimers formed, there was no true amplification in the non-template controls. For *TP53_3*, the no cDNA control peak height corresponded with the experimental group, indicating that this was a true primer dimer. Secondary peaks may occur for several reasons beyond primer dimers, including non-specific amplification, amplicons with secondary structures, and long amplicons with uneven GC content distribution^17^. Importantly, the *TP53*_3 primer pair was generated de-novo by our algorithm with a lower PICKR score (70-80) while *TP53*_1 (90-100 score) that appeared in ~19 published articles was specific, underscoring the predictive power of community validation within our algorithm.

We then identified the top 3 annealing temperatures at which each of the 154 primers amplified best, i.e., were in the top 3 highest peaks on the melting-curve plots (Supplementary Fig. 3-33). We then compared the performance of these to the performance of the primers at the temperature closest to the theoretical T_m_. We observed that the top three best performing T_m_ had empirical optima averaged 3.0 ± 0.1°C above predicted T_m_ corresponding to an average 1.1 ± 0.2 CT value below predicted T_m_ (Supplementary Fig. 34A-B). This indicates that the selected primers with our experimental setup and choice of chemistry (SYBR Green) for RT–qPCR amplified better at higher T_m_ than predicted. However, among the annealing temperatures tested, 61.4°C was the closest experimentally tested temperature to the majority of the theoretical temperatures (Supplementary Fig. 34C) at which the performance of the primers was only marginally affected with 0.8 ± 0.7 CT above the best performing annealing temperature (Fig. 5F-G), strongly indicating that researchers are able to perform the assay at 61.4°C without compromising amplification efficiency. However, for genes with low expression, melting curve optimization may still be required to lower CT values above threshold of no amplification (generally >35-37 CT). When comparing the performance of 5 genes tested across all five PICKR bins, we observed that on average the CT values were 2.1 cycles lower for low-scoring pairs with increased variance (Fig. 5H; *p* = 0.14). For genes tested across PICKR score bins, there was moderate correlation (R^2^ = 0.101, p = 0.02) between pairs with high shared citation counts as opposed to de-novo pairs generated using our algorithm (Fig. 5I and Supplementary Fig. 35). Reduced correlation could be due to having very narrow boundaries for these parameters in our initial algorithm to weed out primer sets that have great differences in GC content and melting temp, i.e., primers that are likely to fail in experimental validation. Further, to assess cross-species performance, we tested 24 mouse primer pairs with PICKR scores >80 (Supplementary Data 2) using NIH/3T3 murine fibroblast cells. Of these, 95% amplified with 12% having double peaks (83% success rate; Fig. 5J and Supplementary Fig. 36-39), similar to the amplification rate of human PICKR scores >80 (Fig. 5A), supporting the generalizability of the PICKR scoring across species.

**Fig. 6:**
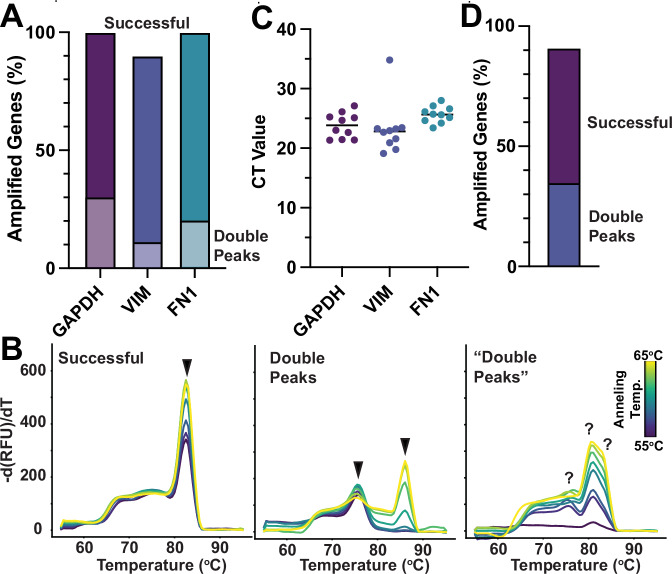
Validation Breadth and Depth with Primer-BLAST. **A** Plot of amplification success for 10 primer sets/gene generated using Primer-Blast. **B** Three example GAPDH primer plots of a normal single peak, double peak, and questionable melt curve, with the latter categorized as double peak for simplicity. Peaks are indicated by arrowheads, while questionable curves are denoted by question marks. **C** Plot of the same samples now illustrating CT values when amplification occurred. *n* = 10 primers/gene. **D** Plot of the amplification success rate and double peak rate for 46 additional primer sets designed in Primer-BLAST (using the first primer on the results list). Source data are provided as a Source Data file. The code used in the creation of these data are published under a CC-BY-NC-ND license (https://creativecommons.org/licenses/by-nc-nd/4.0/deed.en).

To benchmark PICKR against existing tools, we compared its performance with Primer-BLAST as it is one of the most widely used primer design tools ( ~ 8000 citations)^9^. We tested both the depth (per-gene quality) and breadth (cross-gene reliability) of Primer-BLAST. For depth, we tested the amplification of 10 human primer pairs each for *GAPDH*, *VIM*, and *FN1* generated using Primer-BLAST in IMR90 human fibroblasts (Supplementary Data 5). Overall, we found that almost all primer pairs amplified (Fig. 6A), though ~20% of the amplicons had double peaks (Fig. 6B and Supplementary Fig. 40–42) and the CT values were less consistent (Fig. 6C), even from the same template. Although we broadly categorized them as double peaks, some of the amplifying primers have additional features such as delayed amplification or abnormal curve shapes, suggesting suboptimal design despite successful amplification (Fig. 6B). For breadth benchmarking in IMR90 human fibroblasts, we randomly selected 46 human primer pairs generated by Primer-BLAST for genes previously tested in the 90–100 PICKR score group (Fig. 5A and Supplementary Data 5). We observed an ~89% amplification rate but a 35% double peak rate (i.e., a 54% success rate; Fig. 6Dand Supplementary Fig. 43-50) making outcomes likely worse than PICKR, especially for human PICKR scores >80. Given these data, we believe that the PICKR approach can save significant costs and help the community build consensus for high-performance primers.

### Database deployment and user experience

**Fig. 7:**
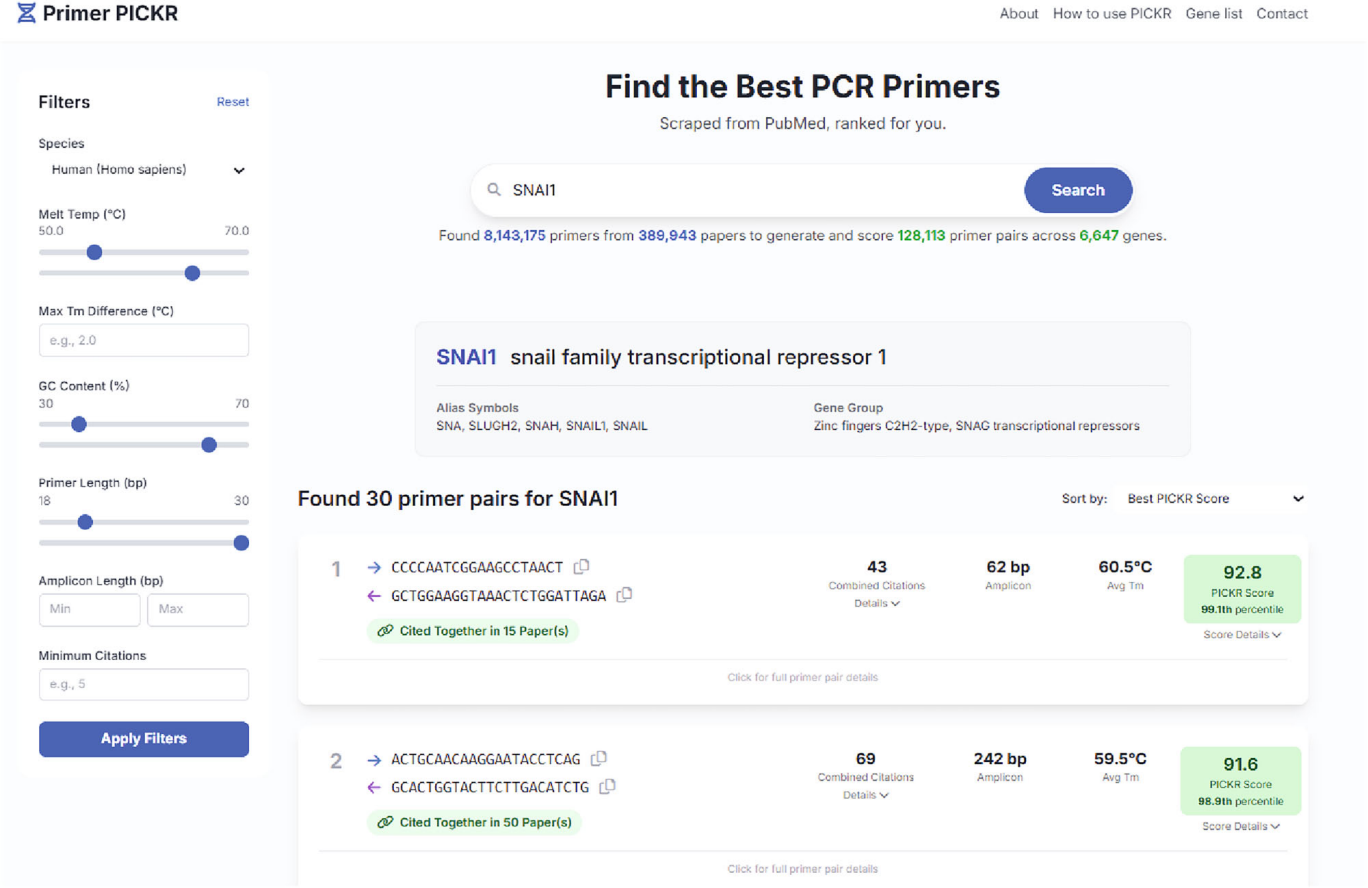
Screenshot of the homepage of www.primerpickr.com. Screenshot of the Primer PICKR website displaying search results for the human gene SNAI1. The interface includes adjustable filters for species, melting temperature, GC content, primer length, amplicon length, and minimum citation count (left panel). For each primer pair, the results display the forward and reverse primer sequences, combined citation count, predicted amplicon size, average melting temperature, and overall PICKR score with percentile ranking.

To host our tool for all to access, we built the primer PICKR database using Django with PostgreSQL and pre-computed BLAST indices, enabling gene queries in <150 ms. The search bar accepts HGNC symbols or common aliases. Results appear as sortable cards listing PICKR score, T_m_, GC, amplicon length, ΔT_m_, shared citations, and total citations (Fig. 7). Left-hand filters allow users to constrain any parameter—for instance, pairs with ΔT_m_ ≤ 1°C, GC 45–55%, amplicon 70–120 bp, and ≥5 shared citations. Clickable primer cards have the following features: (i) one-click clipboard copying of each oligo; (ii) forward and reverse primer characteristics, e.g., T_m_ and GC content; (iii) full list of citing articles with PubMed hyperlinks; (iv) breakdown of evidence, biophysics, and synergy contributions under more score details; and (v) explicit flags for any off-target hits ≤ 4 mismatches (Supplementary Fig. 51). A “Gene List” page also enumerates all supported loci (currently 5,193 human, 2,383 mouse, 537 zebrafish, etc.) and refreshes monthly via automated pipeline. The database is hosted on PythonAnywhere for $6 a month with multiple workers, allowing for simple long-term hosting at a low price. This enables the database to handle millions of queries per day. Current maximum usage has been 10,000 queries in 12 hours with minimal impact on loading times, with 100,000 queries since launch (mostly web crawlers).

## Discussion

Primer PICKR converts three decades of disparate practice into a single, literature-curated, quantitative list of RT–qPCR primer reliability. The approach differs fundamentally from hand-curated repositories: by mining the literature directly, we capture all disclosed sequences and harness collective usage as an implicit performance metric. The power of co-citation is evident as none of the 62 primer pairs with ≥1 shared citation failed in our validation. Yet, 31% of primers are still used only once, with many scientists running into fresh primers failing a majority of the time. Our ranking algorithm identifies the consensus sequences that genuinely ‘work.’

While we believe that this is a great step forward for assay reproducibility, the resource could be further refined in several ways. For example, our PDF parser was still incomplete, and there are likely thousands of uncaptured primer sequences in the literature. Given an average of 18 oligonucleotide sequences per paper and ~220,000 papers mapped as potential candidates that we failed to extract sequences from, we may have missed ~3-4 million unique oligos, equating to 30% of our database. This could enable our gene counts to reach 10,000. Future text-mining iterations will incorporate optical character recognition for locked PDFs to further close this gap. This will include the use of Mistral OCR 3 and LLM integration. Due to the enormous number of ways in which scientists list primers in their manuscripts, meta-data extraction for primers was not possible, including primer direction, melt temperatures used, and even which genes primers were linked to. This led to our algorithm needing to rederive all of this information using BLAST and other algorithms. By matching directly to the Ref_seq databases, no primers were lost due missing metadata. Low evidence score also predicted primer pair failure more than any other metric, supporting a 0.5 weighting; although, a flawed primer may propagate as noted by a minority of primers tested with double peaks in their melt curves. Evidence thresholds of 25, 100, and 500 for matched papers, unmatched papers with the correct sequence, and unmatched papers with reverse complement sequences were chosen due to perceived increase in mistrust. All three biophysics sub-parameters were weighed equally as there was no evidence of one factor having more influence than another. Biophysics and synergy scores were less predictive of primer quality when no off-target effects are found, yet off-targets will lead to significant trust issues with primer choice, reflecting the need for a higher weight for biophysics than synergy. An increasing synergy penalty for forward and reverse primers with ΔTm > 0 °C (with 0 for ΔTm > 3°C) was implemented to increase the likelihood of top primer pairs amplifying the best around 60 °C. A synergy score of 0 was also applied for any primer pairs with complementarity > 5 to limit off-target amplifications. Penalty scores for the types of off-target hits reflect the perceived increase in mistrust of primer pairs. In addition, our validation focused on moderately expressed fibroblast genes; low-abundance transcripts or complex clinical samples may pose additional challenges.

Rare primer dimers and unexplained melt-curve artefacts highlight the continued need for empirical validation: PICKR aims to minimize, not abolish, wet-lab QC. While our database covers over 5,000 human genes, many less-common genes may not be present or have only a few citations with a poor score. Given that only 15% of sequences are even used 3 or more times, a gene may be studied dozens of times with RT–qPCR yet not appear in our database. Furthermore, to reduce the instances of false positives, sequences only containing standard bases (A, C, T, and G) were used. This led to a loss of valid primer pairs that limit our database’s ability to suggest primers which identify gene families or homologs where exact sequences vary. Our database also has limited representation of genes for less studied species which share less genetic overlap with humans, such as C. elegans or Zebrafish, due to the inherent nature of crowd-sourcing. In addition, our database is limited to SYBR Green qPCR, as the requirement for proprietary, probe-dependent design in TaqMan assays precludes large-scale, cross-study primer reuse and ranking. Finally, our data suggest that the best CT values per gene moderately correlated with the  number of shared citations. Yet as use of the PICKR tool increases with well-validated sequences, the predictability of primer pairs that will amplify with the best efficiency will improve beyond the current correlation.

Despite these limitations on a literature-curated approach, our experimental results demonstrate robustness in this database. Scores over 80 from the PICKR algorithm had a ~ 99% amplification rate among the 83 genes tested. Below this threshold, there was still a high chance of amplification ~85%, even for scores in the 50 s. These 83 primer pairs represent 4.1% of our 2,017 primer pairs over a score of 80. Applying the Wilson Score Interval gives us a 95% confidence interval of a true amplification efficiency of all primers with PICKR scores >80 as 93.6 to 99.8%. Further validation will come from community-tested sequences that cite our manuscript which will be highlighted on the database. We attribute this success rate to our stringent inclusion criteria for sequences from literature. One notable discrepancy is theoretical melt temp which is based on a generic master mix; our particular master mix for SYBR Green shifted the experimental optimal T_m_ 3°C higher than the theoretical. However, even with a shift of 3°C, we were only off by about ~0.8 CT, and ~95% of genes were still amplified with CTs below 36 with 87% below 32 CT. Therefore, even if users have different master mixes, this data give us confidence that most users only need to run RT–qPCR once using our theoretical melt temperature and picking primer pairs with scores greater than 80 and achieve reliable amplification. In cases where genes failed to amplify at 61.4 °C, they had average CT values of 37.3. This suggests that even our failed primers may amplify at a lower efficiency where amplification can be seen in cell lines with orders of magnitude higher gene expression. Critically, a unique benefit to the Primer PICKR database is that users who purchase multiple pairs and only publish the pair with the best CT values will help ideal pairs shift up in rank, enabling the community to experimentally determine the most efficient pairs and improve PICKR as a tool over time and increase the amplification likelihood above 98.8%.

Primer PICKR promises to shorten assay development, reduce reagent consumption and improve cross-study comparability. The resource is complementary to Primer-BLAST, which remains essential for custom targets or splice-variant discrimination, and to MIQE guidelines, which mandate transparency but not selection rationales. Ultimately, we envision Primer PICKR becoming a de-facto starting point for RT–qPCR, with genes missing from our dataset being designed with Primer-BLAST or other tools. Primer PICKR is freely accessible, accepts user feedback, and releases monthly snapshots under a CC-BY license. We invite the community to submit validation data, report anomalies, and propose new species references. Widespread adoption by the research community will rapidly reinforce effective primer pairs through citation-driven score improvements, thereby organically elevating the quality of recommendations. Ultimately, this dynamic, collective approach will have profound benefits, especially for under-studied genes and less-common model organisms.

## Methods

### Text mining and filtering

We queried Europe PMC programmatically in batches of 1,000 papers for RT–qPCR–related open-access articles published since 1990. Weekly date ranges were generated and passed to the REST API; returned PMCID lists were capped at 1 × 10⁶ hits per query. For each paper, we retrieved full-text XML and, when available, supplementary PDF or DOCX files. XML tables were parsed with *lxml*; PDF tables were extracted with Camelot (lattice + stream modes), and DOCX tables with *python-docx*. Candidate <100mer strings were detected by a DNA-specific regular expression, stripped of 5′/3′ indicators and orientation prefixes, and screened for valid IUPAC bases (A, C,T,G) and a length of 18–40 nt. Summary statistics are shown in Supplementary Data 6. Putative gene names were assigned by proximity to HGNC-validated symbols and table headers. Sequences failing length, alphabet or self-complementarity heuristics were discarded. Duplicates across sources were removed on the tuple {PMCID, gene, sequence, orientation, source, page}. The resulting primer list was saved as monthly snapshots and fed into downstream scoring.

### Primer–transcript alignment and annotation

To determine the gene for each sequence, each unique primer was aligned against NCBI RefSeq-RNA for all species using *blastn-short* (v2.15.0) with dust masking disabled, *max_target_seqs* = 25 and 24 CPU threads. A local human database (makeblastdb –dbtype nucl) ensured reproducible performance. BLAST output (fields = qseqid, sacc, stitle, staxids, bitscore, e-value, qcovs) was parsed with Pandas; only hits exhibiting 100 % query coverage were considered for gene assignment. Gene symbols were extracted from the RefSeq definition line by regex and validated against a pre-cached HGNC catalog ( ≈  44k entries). For each primer, the most frequent symbol among perfect-coverage hits was retained; when no symbol met HGNC criteria, the script recorded the best e-value and later applied a fallback to author-supplied gene lists. Newly annotated primers were merged with prior BLAST results to avoid redundancy. Subsequent modules computed nearest-neighbor melting temperature and GC% (described in more detail separately), ranked primers by literature usage, and identified exact binding coordinates plus reverse-complement partners across six vertebrate transcriptomes, generating per-gene CSV outputs for downstream scoring.

### Primer theoretical melt temperature and GC content calculation

We calculated primer melting temperature (T_m_) with the SantaLucia nearest-neighbor^18^ model implemented in Biopython. For each primer, the dinucleotide enthalpies (ΔH) and entropies (ΔS) were summed and corrected for helix initiation; the entropy was then salt-corrected to the reaction buffer (50 mM Na⁺, 1.5 mM Mg²⁺, 0.2 mM dNTPs) using the Owczarzy mixed-salt term^19^, giving an effective [Na⁺] of 189 mM. The temperature at which half of the duplex is denatured was obtained from:

2 $${{{\rm{Tm}}}}=\frac{\Delta {{{\rm{H}}}}}{\Delta {{{\rm{S}}}}+{{{\rm{R}}}}{ln}({{{\rm{C}}}}/4)}-273.15+16.6{\log }_{10}[{{{{\rm{Na}}}}}^{+}]$$

where R = 1.987 cal mol^−1^ K^−1^ and a strand concentration *C*_*t*_ = 250 nM. This procedure reproduces experimental T_m_ values within ~1–2 °C for 18–25 nt RT–qPCR primers. Finally, we report GC content as the percentage of guanine and cytosine bases in the primer; this, together with T_m_, feeds the biophysical component of the PICKR score.

### Pair generation and metadata enrichment

Annotated single-primer files were first stratified by species and gene symbol. Forward primers (orientation = ‘forward’) were exhaustively paired with reverse-complement primers (orientation = ‘reverse_complement’). Pairs were retained only when their genomic coordinates predicted an amplicon between 50 bp and 300 bp and when self-complementarity of each primer and inter-primer complementarity (calculated as the longest contiguous reverse-complement match) did not exceed six nucleotides. For each species, all unique primers present in the surviving pairs were concatenated into a single FASTA and queried once against the corresponding species-specific RefSeq-RNA BLAST database (blastn-short, 100 % query coverage, ≤ 4 mismatches, 24 threads). Results were cached in memory; for every mismatch class (0–4) the script stored the set of off-target gene symbols and, when mismatches occurred, a position-encoded pattern of the substituted bases. These cross-specificity annotations were then appended to every primer pair row. Finally, global sequence→PMCID mappings from the raw corpus were overlaid to compute publication lists and citation counts for each forward, reverse and reverse-complement sequence, as well as their intersection.

### Primer scoring and ranking

Each candidate pair received a composite PICKR score (0–100) built from three orthogonal terms. Evidence (weight 0.50) quantifies prior community use: log-scaled counts of publications that cite both primers together (shared), each primer individually (regular), and the sum of regular + inverted-orientation citations, normalized to ceilings of 25, 100 and 500 articles, respectively. Biophysics (0.30) averages individual primer quality with three components: length, GC-content and nearest-neighbor T_m_ compliance. A multiplicative specificity penalty derived from all off-target BLAST hits carrying 1–4 mismatches was also used with perfect off-targets or >6-nt self/hetero-dimer runs receiving a zero score. Synergy (0.20) penalizes ΔT_m_ between primers and residual dimerization. Scores were computed row-wise, converted to percentiles (μ = 54.6, σ = 16.1) and pairs scoring zero were discarded. For each gene–species combination, rows were sorted by PICKR and the top 30 retained.

### Cell culture

IMR90 human lung fibroblasts (ATCC CCL-186) and NIH/3T3 (ATCC CRL-1658) were cultured in DMEM (Thermo Fisher Scientific 11965092) with 10% fetal bovine serum (Omega Scientific FB-01) and 1% penicillin streptomycin (Thermo Fisher Scientific 15140122). Culture media was replaced every two days.

### RNA extraction, cDNA synthesis and RT–qPCR

Total RNA was extracted using RNeasy Mini isolation kits (Qiagen 74136) and a NanoDrop spectrophotometer (ND-2000, Thermo Fisher) was used to obtain RNA concentration. SuperScript™ IV First-Strand Synthesis System (Invitrogen™ 18091050) with MJ Mini Personal Thermocycler (BioRad) was used for cDNA synthesis from 4 µg of RNA per reaction. We performed RT–qPCR reactions (PowerUp SYBR Green, Applied Biosystems, 4367659) using 10 ng cDNA and 1 μM primers on the CFX96 Real-Time PCR Detection System (Bio-Rad). Annealing temperature gradients (55–65°C) was used to identify optimal amplification conditions. CT < 36 with single-peak melt curve indicated successful and specific amplification of the target genes.

### RT–qPCR Data Processing and Analysis

All RT–qPCR data analysis was performed using custom Python scripts utilizing the pandas, NumPy, Matplotlib, Seaborn, and SciPy libraries. First, raw RT–qPCR data from multiple experimental plates were automatically aggregated. For each plate, melt curve derivative data and quantification summary data, which included Cycle Threshold (CT) values and experimental annealing temperatures (T_m_), were programmatically located and loaded. This data was merged to create a comprehensive dataset linking every melt curve data point to its corresponding primer set, experimental T_m_, and CT value across all biological replicates. Reactions with missing or non-numeric CT values were excluded from further analysis.

### Performance ranking by melt curve analysis

To determine the optimal experimental conditions for each primer set, a performance ranking was conducted based on melt curve peak height. The maximum value of the first derivative of the melt curve (-d(RFU)/dT), representing the main peak height, was identified for each replicate. These peak heights were then averaged across all biological replicates for each unique combination of primer set and experimental T_m_. For each primer set, the experimental T_m_s were ranked based on their average peak height in descending order. The condition yielding the highest average peak was assigned Rank 1, the second highest was Rank 2, and the third highest was Rank 3. The top three performing experimental T_m_’s and their corresponding average CT values were selected for subsequent analysis. For reactions exhibiting secondary melt peaks and passing CT thresholds, PCR products were purified and subjected to Sanger sequencing. Sequence reads were aligned to the Homo sapiens reference genome (taxid:9606) using BLASTn (core_nt), and classified as on-target when the top hit exhibited ≥90% query coverage, ≥90% percent identity, and an E-value ≤ 1 × 10⁻¹⁰.

### Primer specificity and off-target amplification analysis

To ensure that the analysis was performed only on reactions with successful amplification, any reaction with a CT value greater than 36 or a missing CT value was excluded from the specificity assessment.

### Data-driven thresholding and peak detection

A robust, data-driven approach was implemented to distinguish genuine off-target peaks from baseline noise. First, the maximum melt curve peak height was determined for all validly amplifying reactions (CT ≤ 36). An absolute noise floor was then established by calculating the 10^th^ percentile of these main peak heights, effectively creating a threshold based on the lower limit of successful amplification signals in the dataset. For each individual reaction that passed the CT filter, the number of significant melt curve peaks was determined using the find_peaks function from the SciPy library. A peak was considered significant if its height exceeded both the calculated absolute noise floor and a relative height threshold of 50% of the main peak’s height for that specific reaction. The final specificity for each primer set was determined by examining the peak counts across all its biological replicates and experimental temperatures. The maximum number of significant peaks observed for a given primer set was identified. If this maximum count was greater than one, the primer set was classified as having “Multiple Peaks”; otherwise, it was classified as “Specific”.

### Calculation of Performance Metrics

The top three ranked conditions for each primer set were merged with a primer annotation file containing computationally predicted features, including the theoretical melting temperature (Theoretical_T_m_). ΔT_m_ and ΔCT were calculated as performance metrics. For ΔT_m_, the difference between the theoretical and experimental melting temperatures (Theoretical_T_m_ - Experimental_T_m_) was calculated for each of the top three ranked conditions to assess the accuracy of the in-silico prediction. To quantify the performance difference between an experimentally determined optimal condition and the condition suggested by theoretical T_m_, the ΔCT was calculated as (Average CT of the ranked condition) - (Average CT at the experimental T_m_ closest to the Theoretical_T_m_). A negative value indicates that the ranked condition was more efficient. To determine the ΔT_m_ and ΔCT of the experimentally top ranked condition and 61.4°C, we performed the above calculations where Theoretical_T_m_ was replaced with results we obtained at 61.4°C.

### Normalized CT Regression Analysis

To compare the performance of different primer designs within the same gene set (e.g., GAPDH from 90-100, GAPDH from 80-90, etc.), a normalized CT analysis was performed. This analysis was restricted to the best-performing (Rank 1) primer sets that exhibited robust amplification (average CT ≤ 36). Within each gene set, the primer with the highest PICKR score was identified, and its average CT value was used as the reference. The CT values for all other primers in that set were then normalized against this reference using the formula: Normalized CT = (Reference CT) - (Primer’s Avg CT). A positive Normalized CT value indicates a more efficient primer (lower CT) compared to the reference. Linear regression analysis was performed to evaluate the relationship between the Normalized CT and various primer features (e.g., PICKR score, Diff_T_m_, GC content). For each regression, the coefficient of determination (R²) and *p*-value were calculated, and plots were generated showing the regression line with a 95% confidence interval. Data visualizations were generated using Matplotlib and Seaborn.

### Statistics & Reproducibility

Success-rate differences were assessed by one-way ANOVA, but for nonparametric datasets with multiple comparisons, Friedman’s test was used. Correlation strength is measured by Pearson r. Expectation-values were computed by BLASTn based on Karlin–Altschul statistics. No statistical methods were used to predetermine sample size, and the experiments were not randomized. The investigators were not blinded to allocation during experiments and outcome assessment.

### Reporting summary

Further information on research design is available in the Nature Portfolio Reporting Summary linked to this article.

## Supplementary information

Supplementary Information

Description of Additional Supplementary Files

Supplementary Data 1

Supplementary Data 2

Supplementary Data 3

Supplementary Data 4

Supplementary Data 5

Supplementary Data 6

Reporting Summary

Transparent Peer Review file

## Source data

Source Data

## Data Availability

The full primer database has been deposited in the GitHub database under accession code (https://github.com/tmolley2/PICKR_DB). The raw numbers for charts and graphs are available in the Source Data file whenever possible. Source data are provided with this paper. Figures, tables and, other raw data for validation is available at (10.6084/m9.figshare.31082878). Source data are provided with this paper.

## Code availability

Source code for text mining, alignment and scoring is available at (https://github.com/tmolley2/PICKR). It is further available via a DOI at [10.5281/zenodo.17058652]. This code is licensed under CC-BY-NC-ND 4.0, the Creative Commons Attribution-Non-Commercial-No Derivatives 4.0 International license (https://creativecommons.org/licenses/by-nc-nd/4.0/deed.en).
