# Supplementary material for: Primer PICKR: literature-mined scoring platform for robust RT–qPCR primers
Source: Nat Commun. 2026 May 28;17:6938. doi: 10.1038/s41467-026-73648-2 (PMC13388996; doi:10.1038/s41467-026-73648-2)
Supplement: Supplementary file 1 — Supplementary Information [file 41467_2026_73648_MOESM1_ESM.pdf]

**Primer PICKR: Literature-Mined Scoring Platform for Robust RT–qPCR Primers**

Thomas G. Molley[1,3*], Abhin
aba Banerjee[1,3*], Alis Balayan[2,3*], Jun K. Robbins[1], and Adam J. Engler[1-3†]

[1]Shu Chien-Gene Lay Department of Bioengineering and [2]Biomedical Sciences Program, University of California, San Diego; La Jolla, CA 92093 USA
[3]Sanford Consortium for Regenerative Medicine; La Jolla, CA 92037 USA
[*]These Authors contributed equally to this work.
[†]Corresponding Author: aengler@ucsd.edu
Phone: 858-246-0678
Fax: 858-534-5722

# Supplementary Text

The full math of the PICKR score can be seen here:

$$\textbf{PICKR score:} \quad = \quad 100\,\big(W_E\, e + W_B\, b + W_S\, s\big)$$

$$(W_E, W_B, W_S) = (0.50,\ 0.30,\ 0.20)$$

**Evidence score:** $e_{\text{raw}} = s_{\text{shared}} + s_{\text{reg}} + s_{\text{tot}}$

$$e = \min(e_{\text{raw}},\ 1)$$

$$s_{\text{shared}} = 0.5\ \min\!\Big(\frac{\log_{10}(shared+1)}{\log_{10}(P_{\max}+1)},\ 1\Big),$$

$$s_{\text{reg}} = 0.5\ \tfrac{1}{2}\Big[\min\!\Big(\frac{\log_{10}(f_{\text{reg}}+1)}{\log_{10}(R_{\max}+1)}, 1\Big) + \min\!\Big(\frac{\log_{10}(r_{\text{reg}}+1)}{\log_{10}(R_{\max}+1)}, 1\Big)\Big],$$

$$s_{\text{tot}} = 0.5\ \tfrac{1}{2}\Big[\min\!\Big(\frac{\log_{10}(f_{\text{tot}}+1)}{\log_{10}(T_{\max}+1)}, 1\Big) + \min\!\Big(\frac{\log_{10}(r_{\text{tot}}+1)}{\log_{10}(T_{\max}+1)}, 1\Big)\Big]$$

$$f_{\text{tot}} = f_{\text{reg}} + f_{\text{inv}}, \quad r_{\text{tot}} = r_{\text{reg}} + r_{\text{inv}}$$

Literature citation the strength of prior experimental use

**Biophysics score:** $b = 0.5\,(Q_f + Q_r), \quad Q_j = \frac{f_L(\ell_j) + f_G(\mathrm{GC}_j) + f_T(T_{m,j})}{3};$

For each primer side $p \in \{f, r\}$

$$X_p = \prod_{a \in \mathcal{A}_p} \sigma(d_a, n_a^{\triangleleft}), \quad \sigma(d, n^{\triangleleft}) = \min\!\Big(1,\ \beta_d + (1-\beta_d)\,\rho\,\frac{n^{\triangleleft}}{d}\Big).$$

$$\beta_1 = 0.10, \quad \beta_2 = 0.35, \quad \beta_3 = 0.65, \quad \beta_4 = 0.90, \quad \rho = 0.60$$

Amplicon length, GC content, Melt temperature

**Synergy score:** $s = \max\!\Big(0,\ 1 - \big|T_m^f - T_m^r\big|3 - \max(0, PC - D_cD_c)\Big)$

Differences in melt temp, Complementarity

**Parameters:** $P_{\max} = 25, \quad R_{\max} = 100, \quad T_{\max} = 500$

Here, e is the evidence score, b is the biophysics score, and s is the synergy score. We, Wb, and Ws are the weights for those three scores respectively. Sshared is the evidence component score for the number of pmcids that are shared between a forward and reverse primer. Sregular is the individual pmcids associated with each orientation at that exact direction, and Stot is Sreg but also includes the matched reverse complements for a primer, i.e. a forward sequence for one person was inverted and used as a reverse for another person. Qf and Qr are the component biophysics score for the forward and reverse primers respectively. Fl is the amplicon length function, fg is for GC content, and ft is for melt temperature. Xp calculated the penalty for cross specificity matches, summing up the products of mismatches, with the penalties for mismatches at x hamming distances being equal to Bx. For the synergy score, PC is the pair complementarity score, and DcDc is set to 6. Tm is melt temperature.

## Supplementary Figures

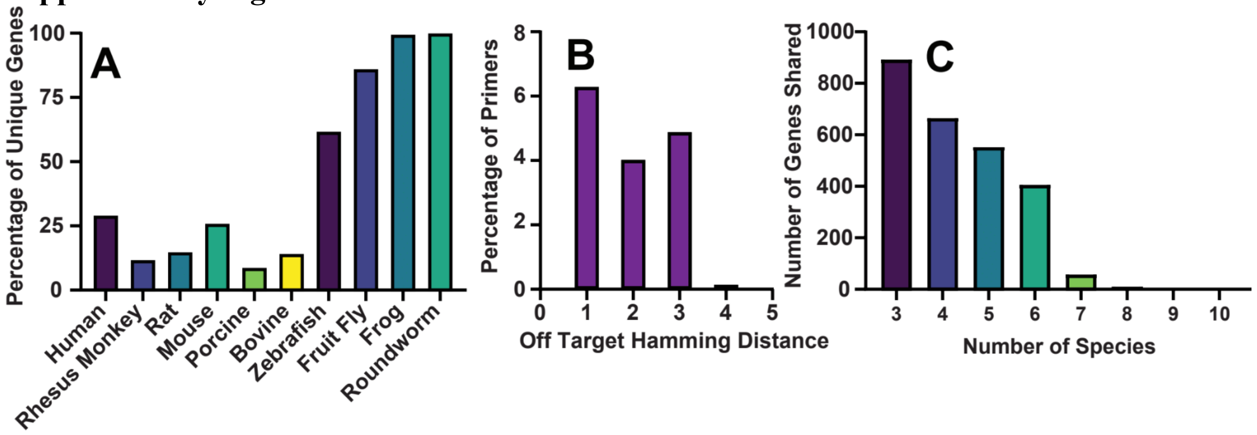


**Supplementary Figure 1: Primer uniqueness. (A)** Percentage of primers for each species that are unique only to that species. **(B)** Percentage of primers with off-target hits at different Hamming distances. **(C)** number of genes that are shared across multiple species. All graphs generated with the 290,071 unique primers used at least 3 times in the literature from the 399,394 papers scraped. Source data are provided as a Source Data file. The code used in the creation of these data are published under a CC-BY-NC-ND license (https://creativecommons.org/licenses/by-nc-nd/4.0/deed.en).

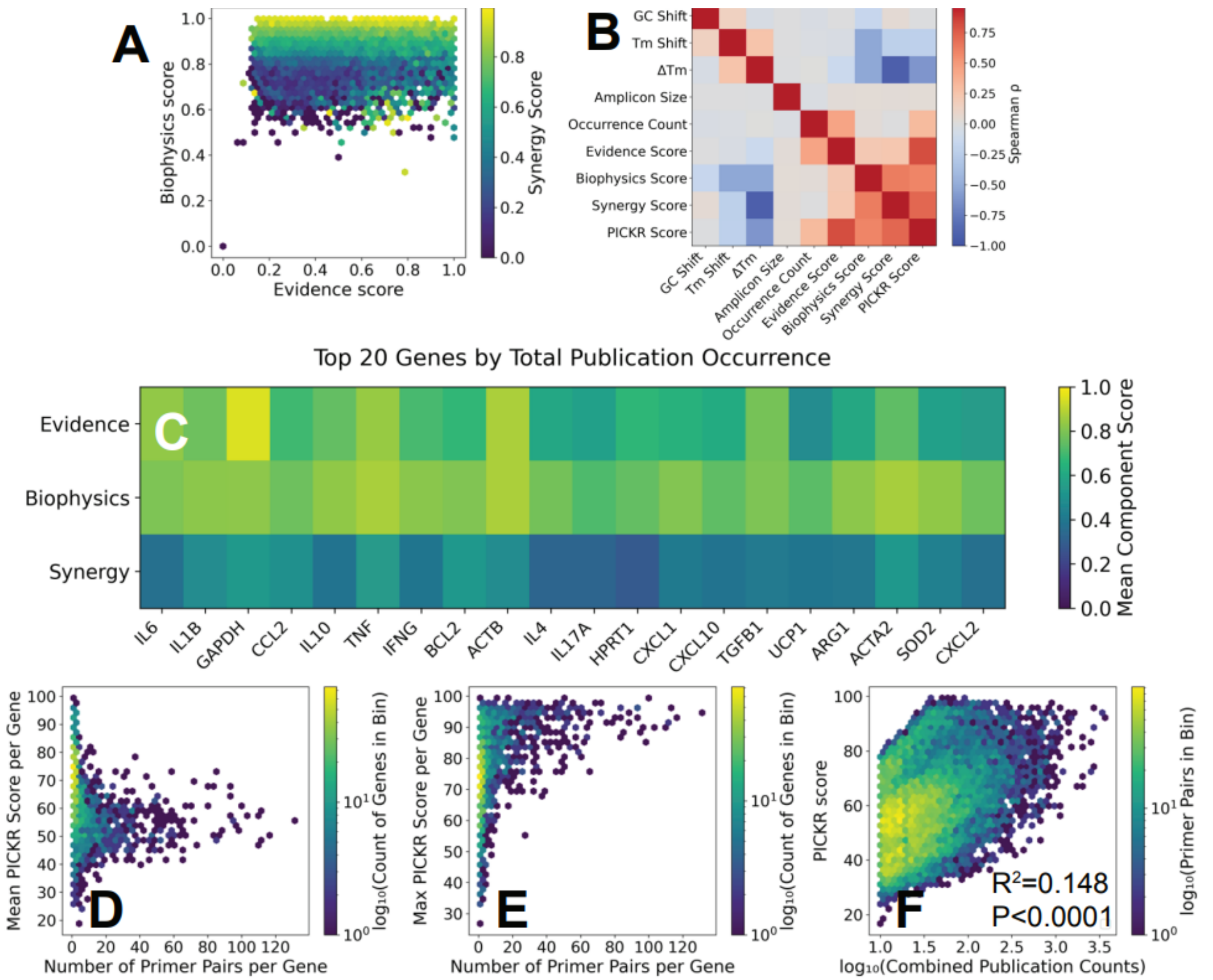


**Supplementary Figure 2: Primer score characteristics for Mouse.** **(A)** Hexbin plot of the three component scores (evidence, biophysics, and synergy). **(B)** Pearson correlation plot of PICKR scores, the component scores, and the meta data used to calculate the scores. **(C)** Component scores of the average primer pair for the twenty most used genes. Hexbin plots of mean **(D)** and maximum PICKR **(E)** score for all human genes. **(F)** Hexbin plot of PICKR score against log transformed publication counts for primer pairs. Sample sizes for all graphs were the 16,529 generated primer pairs derived from the 290,071 primers pulled from the literature. Source data are provided as a Source Data file. The code used in the creation of these data are published under a CC-BY-NC-ND license (https://creativecommons.org/licenses/by-nc-nd/4.0/deed.en).

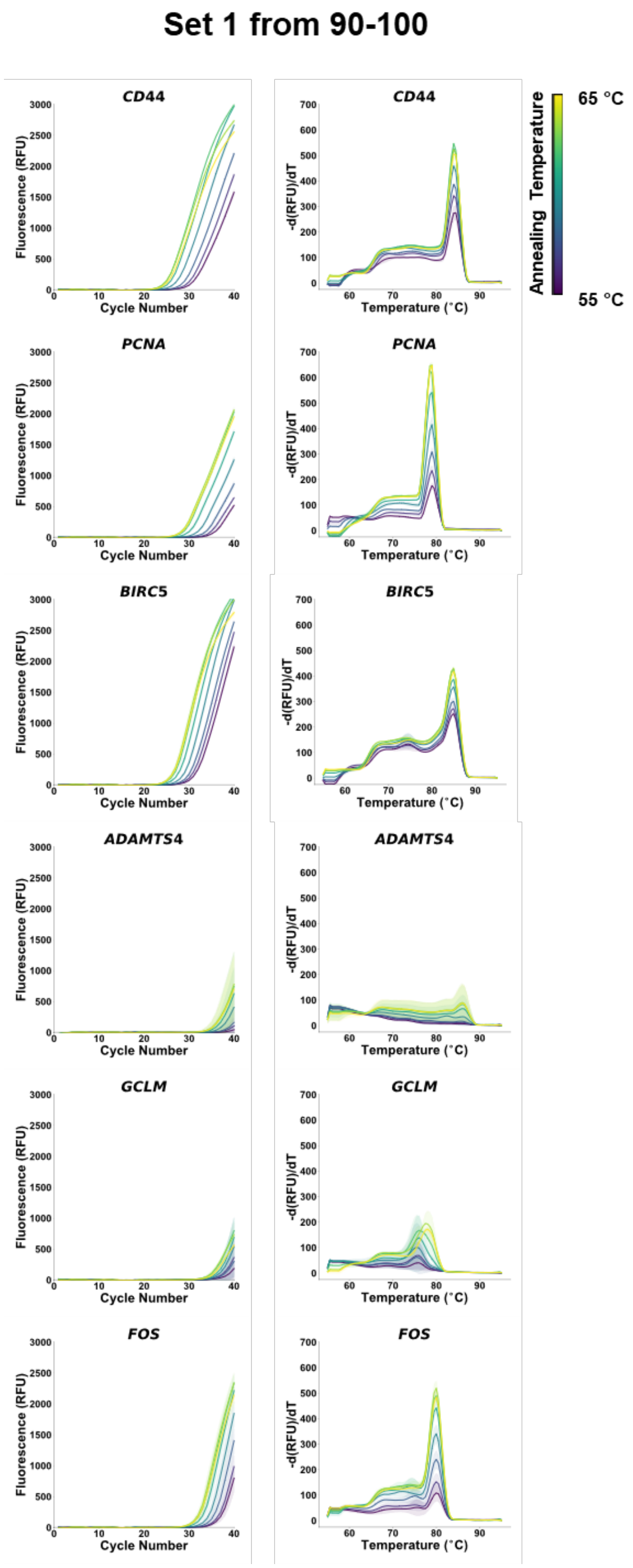


**Supplementary Figure 3. Amplification curves and melt curves for 90-100 bin set 1.** 8 different temperatures were chosen to create amplification curves (left) and melt temperature curves (right). Source data are provided as a Source Data file.

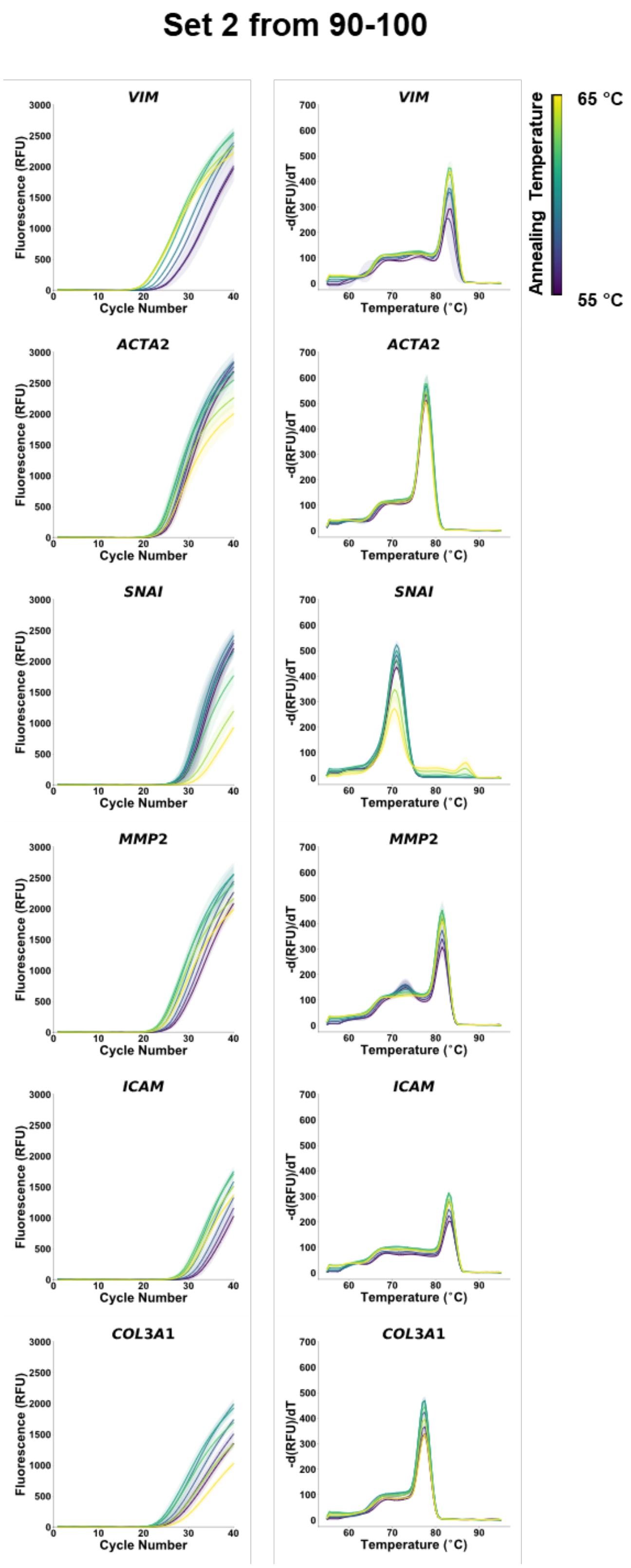


**Supplementary Figure 4. Amplification curves and melt curves for 90-100 bin set 2.** 8 different temperatures were chosen to create amplification curves (left) and melt temperature curves (right). Source data are provided as a Source Data file.

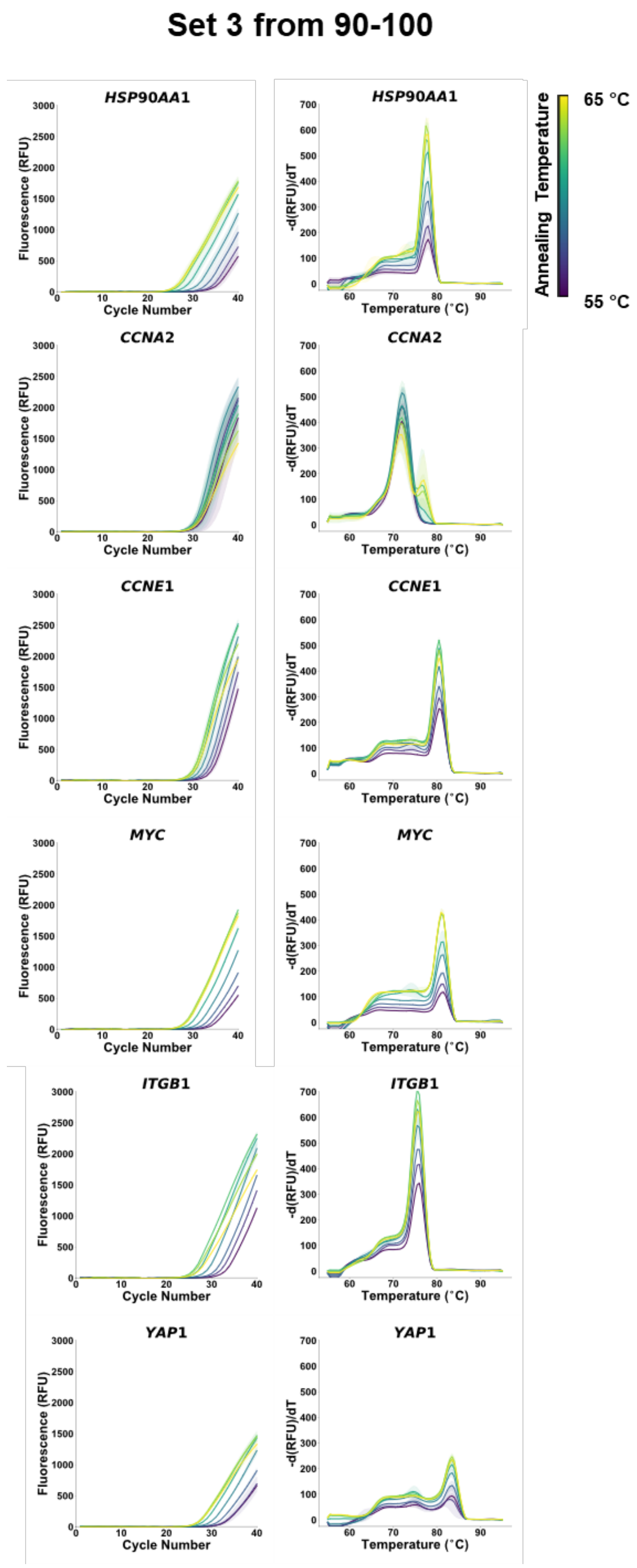


**Supplementary Figure 5. Amplification curves and melt curves for 90-100 bin set 3.** 8 different temperatures were chosen to create amplification curves (left) and melt temperature curves (right). Source data are provided as a Source Data file.

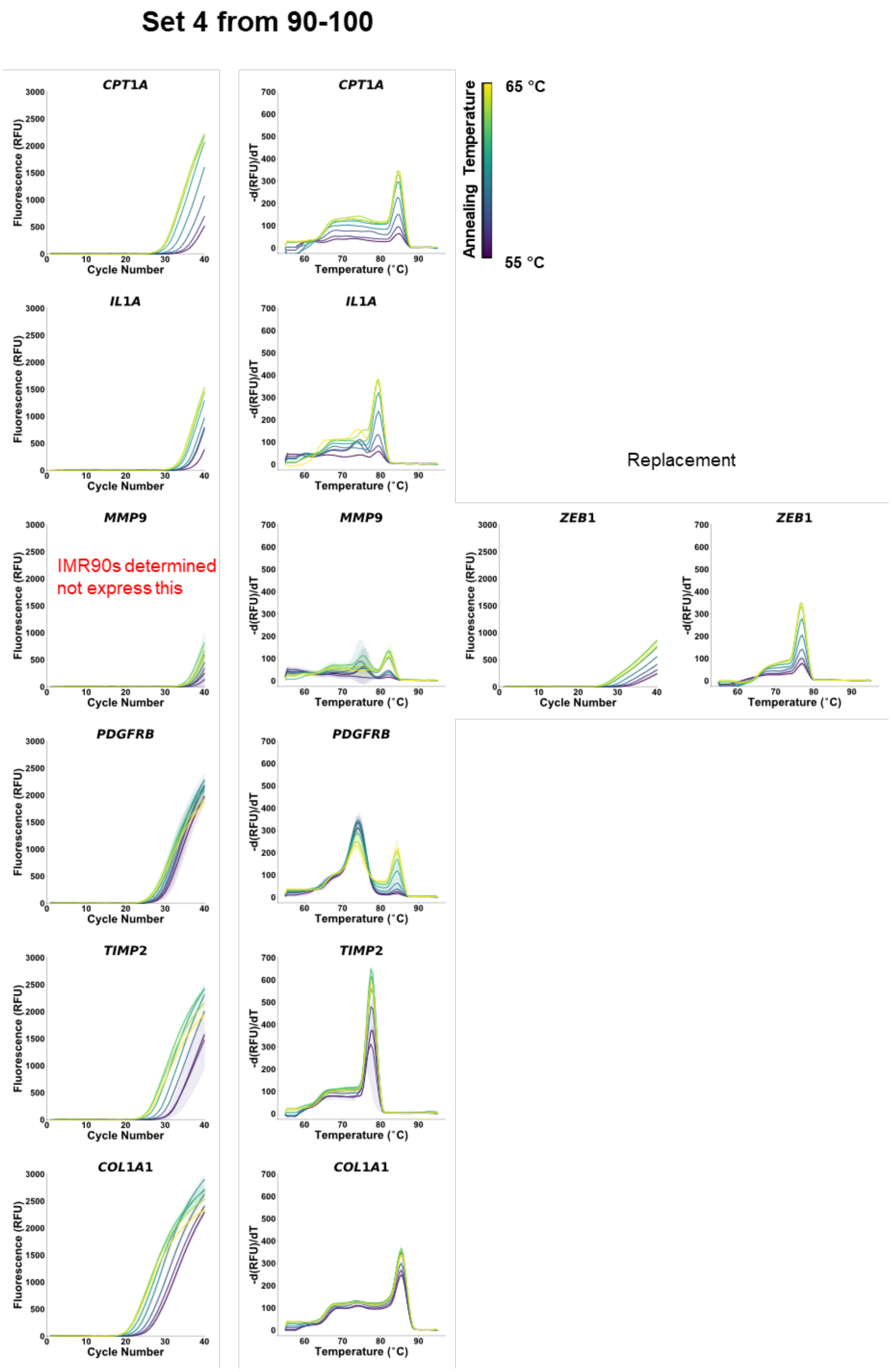


**Supplementary Figure 6. Amplification curves and melt curves for 90-100 bin set 4.** 8 different temperatures were chosen to create amplification curves (left) and melt temperature curves (right). Source data are provided as a Source Data file.

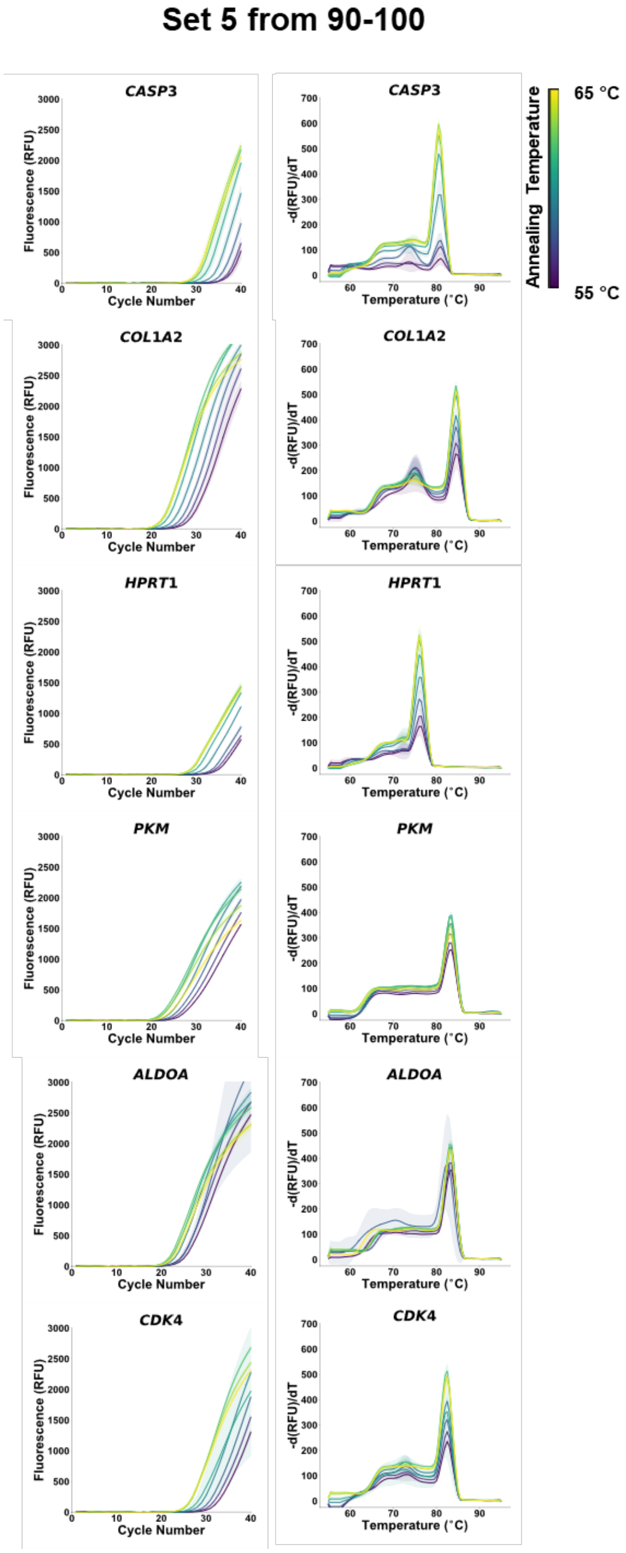


**Supplementary Figure 7. Amplification curves and melt curves for 90-100 bin set 5.** 8 different temperatures were chosen to create amplification curves (left) and melt temperature curves (right). Source data are provided as a Source Data file.

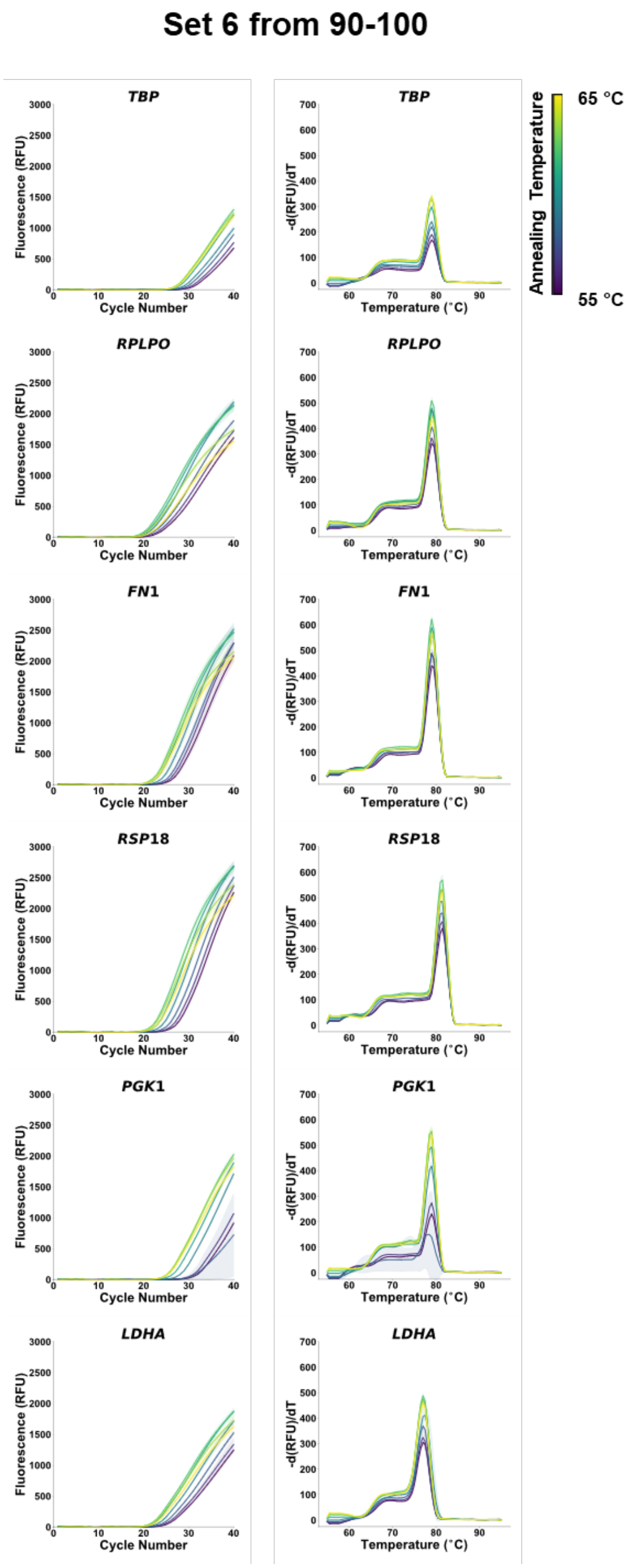


**Supplementary Figure 8. Amplification curves and melt curves for 90-100 bin set 6.** 8 different temperatures were chosen to create amplification curves (left) and melt temperature curves (right). Source data are provided as a Source Data file.

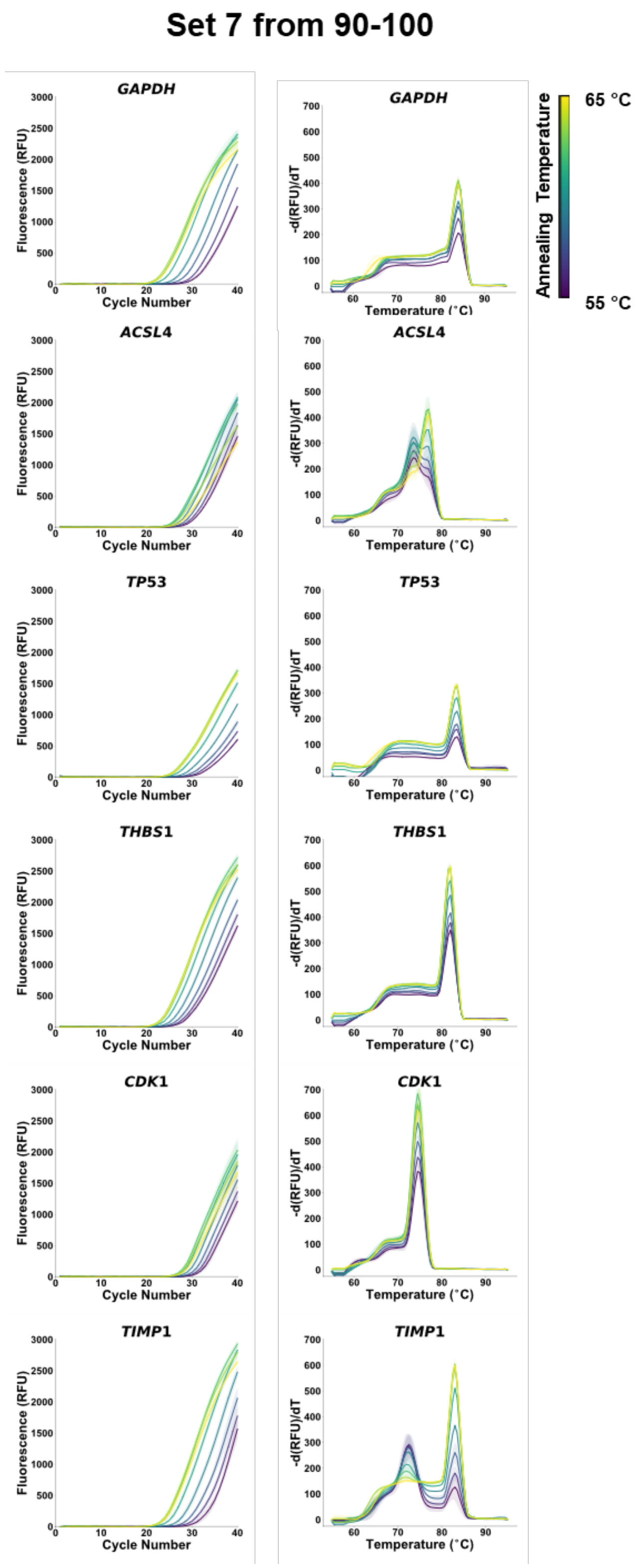


**Supplementary Figure 9. Amplification curves and melt curves for 90-100 bin set 7.** 8 different temperatures were chosen to create amplification curves (left) and melt temperature curves (right). Source data are provided as a Source Data file.

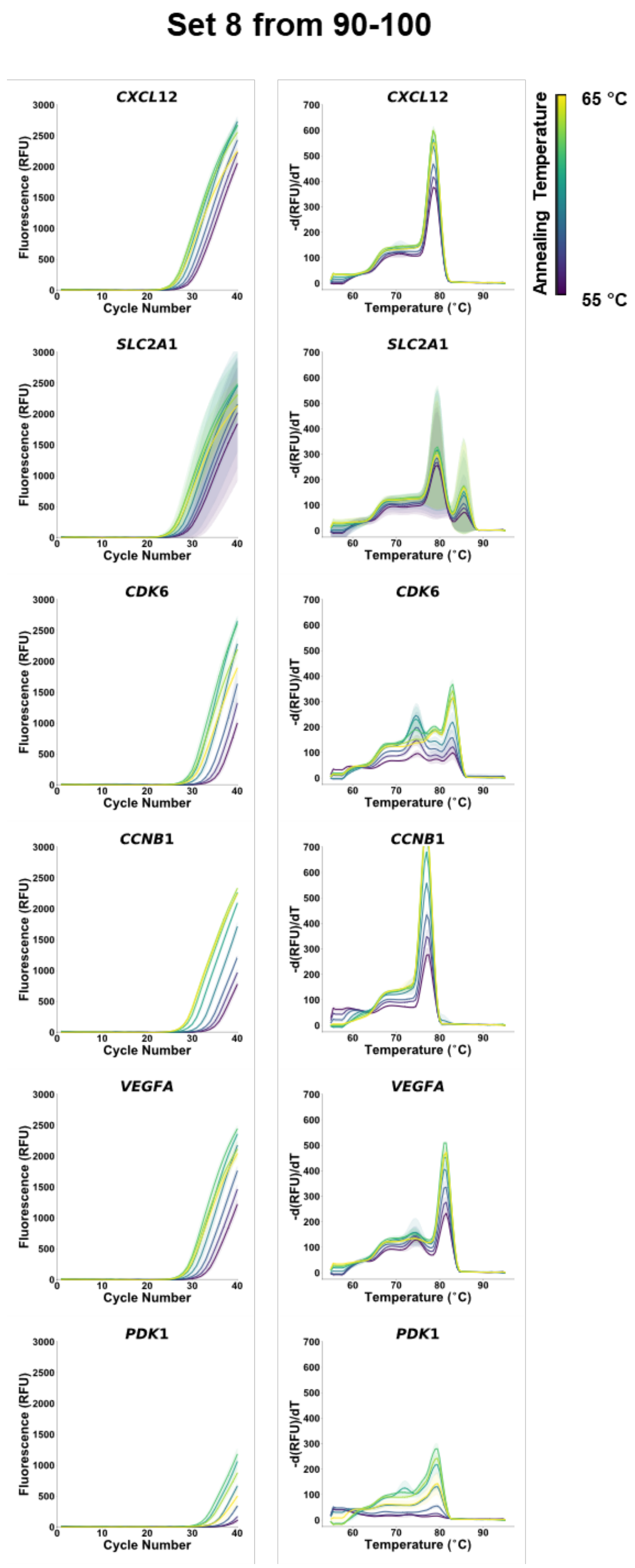


**Supplementary Figure 10. Amplification curves and melt curves for 90-100 bin set 8.** 8 different temperatures were chosen to create amplification curves (left) and melt temperature curves (right). Source data are provided as a Source Data file.

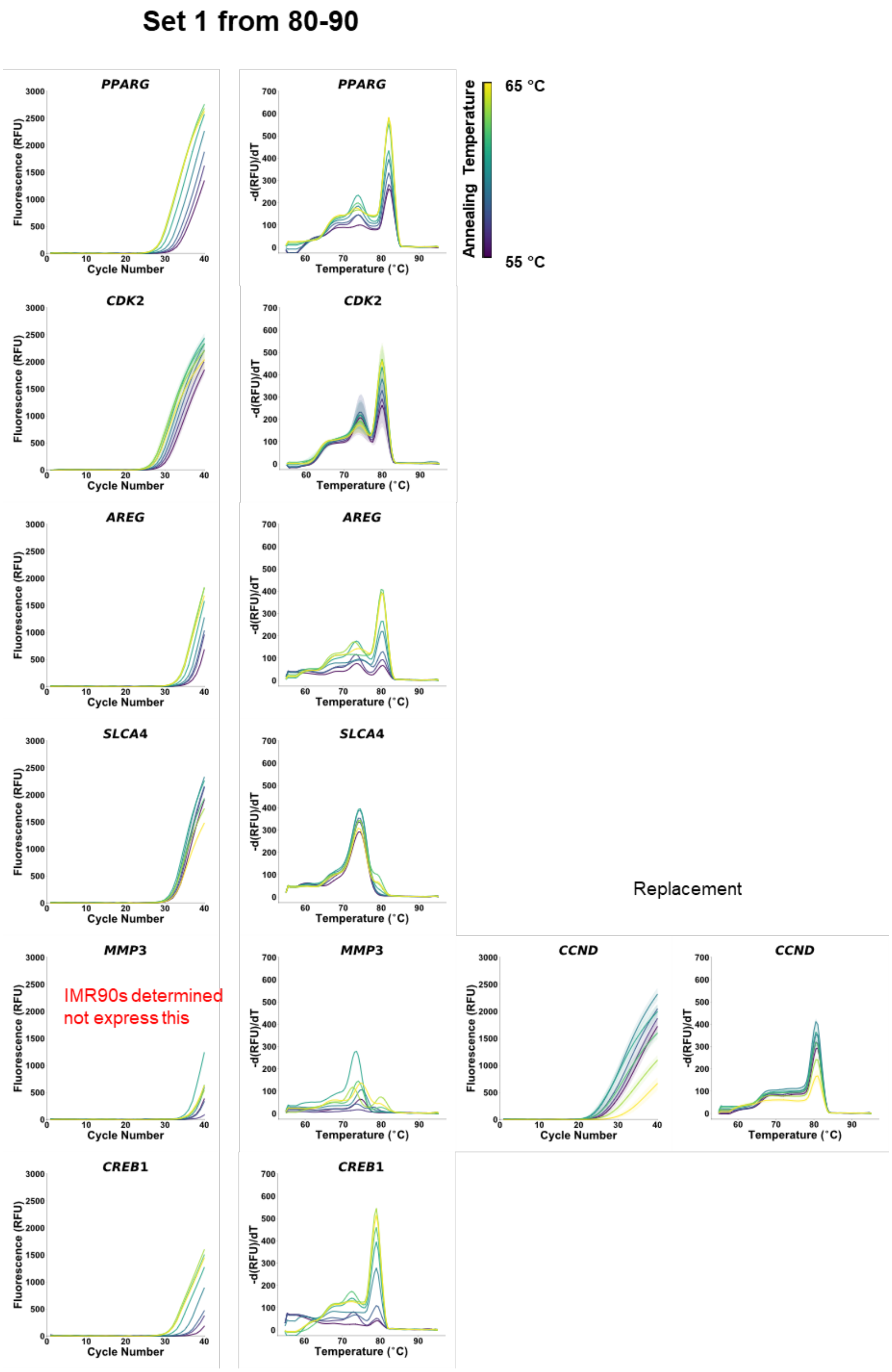


**Supplementary Figure 11. Amplification curves and melt curves for 80-90 bin set 1.** 8 different temperatures were chosen to create amplification curves (left) and melt temperature curves (right). Source data are provided as a Source Data file.

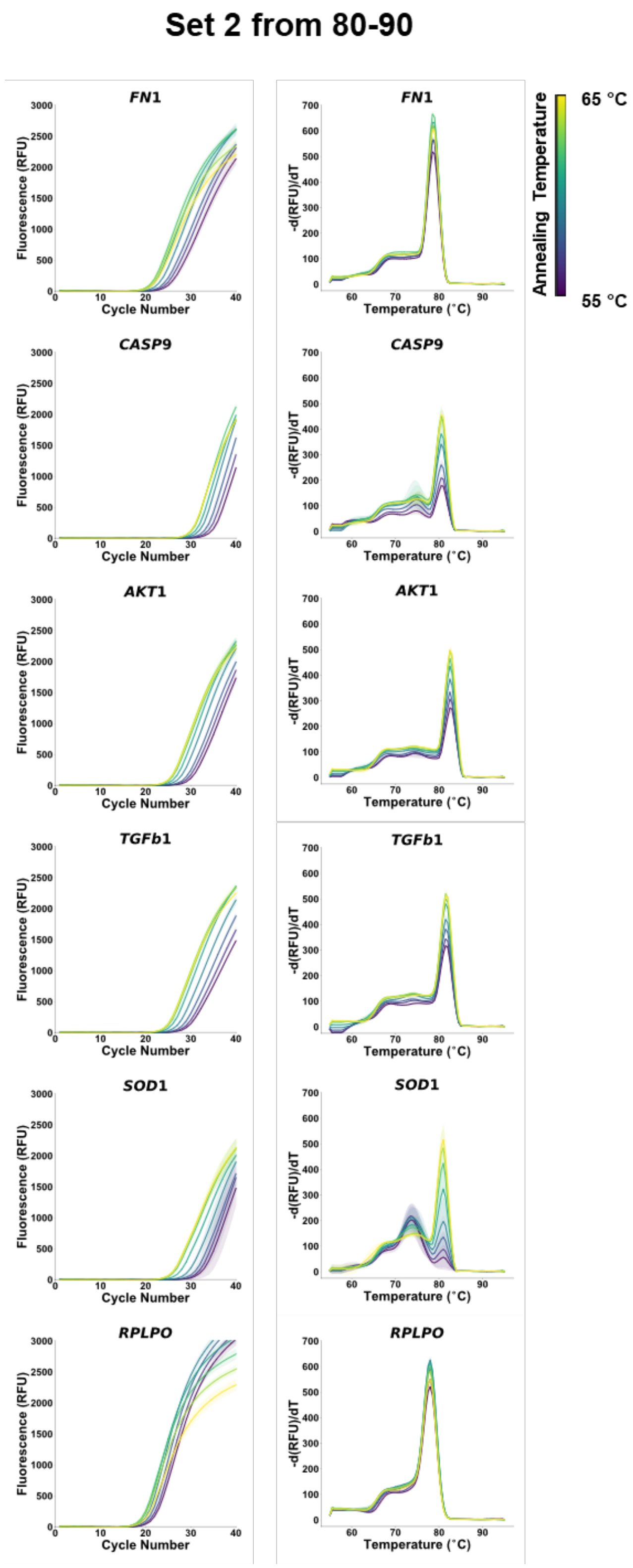


**Supplementary Figure 12. Amplification curves and melt curves for 80-90 bin set 2.** 8 different temperatures were chosen to create amplification curves (left) and melt temperature curves (right). Source data are provided as a Source Data file.

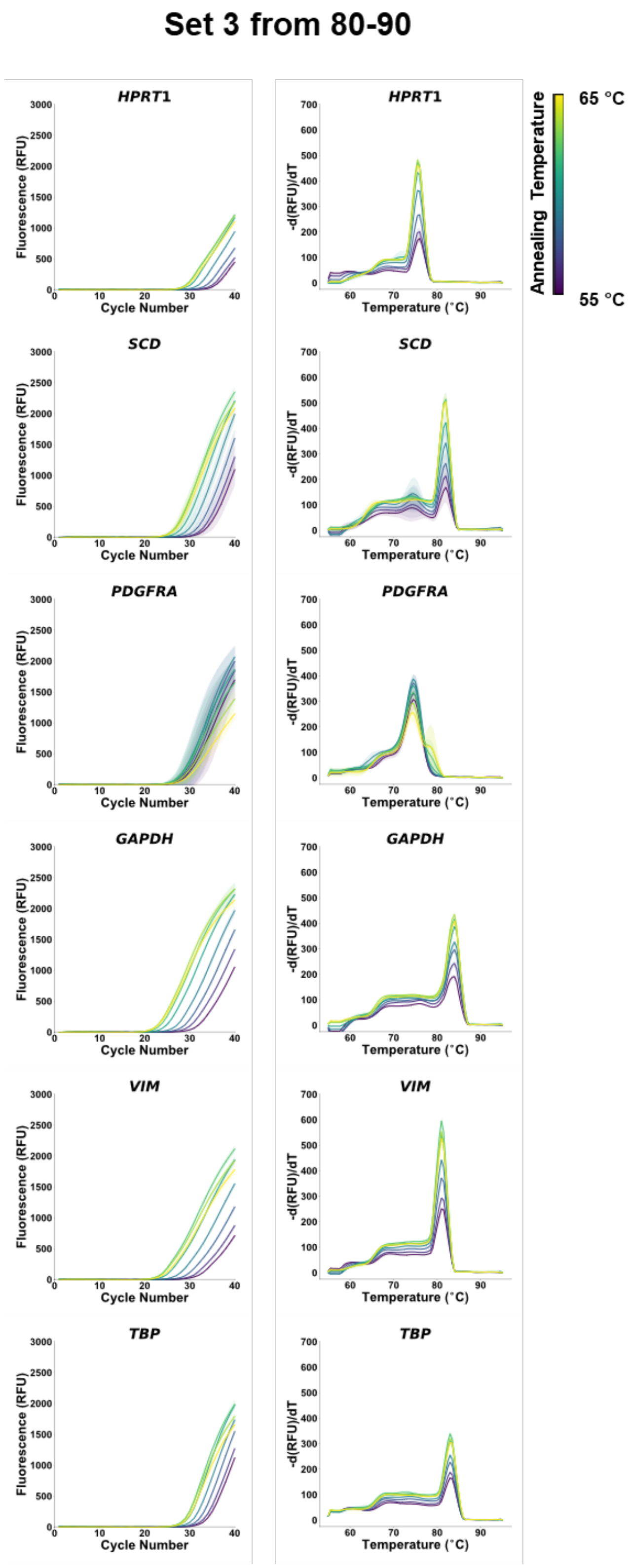


**Supplementary Figure 13. Amplification curves and melt curves for 80-90 bin set 3.** 8 different temperatures were chosen to create amplification curves (left) and melt temperature curves (right). Source data are provided as a Source Data file.

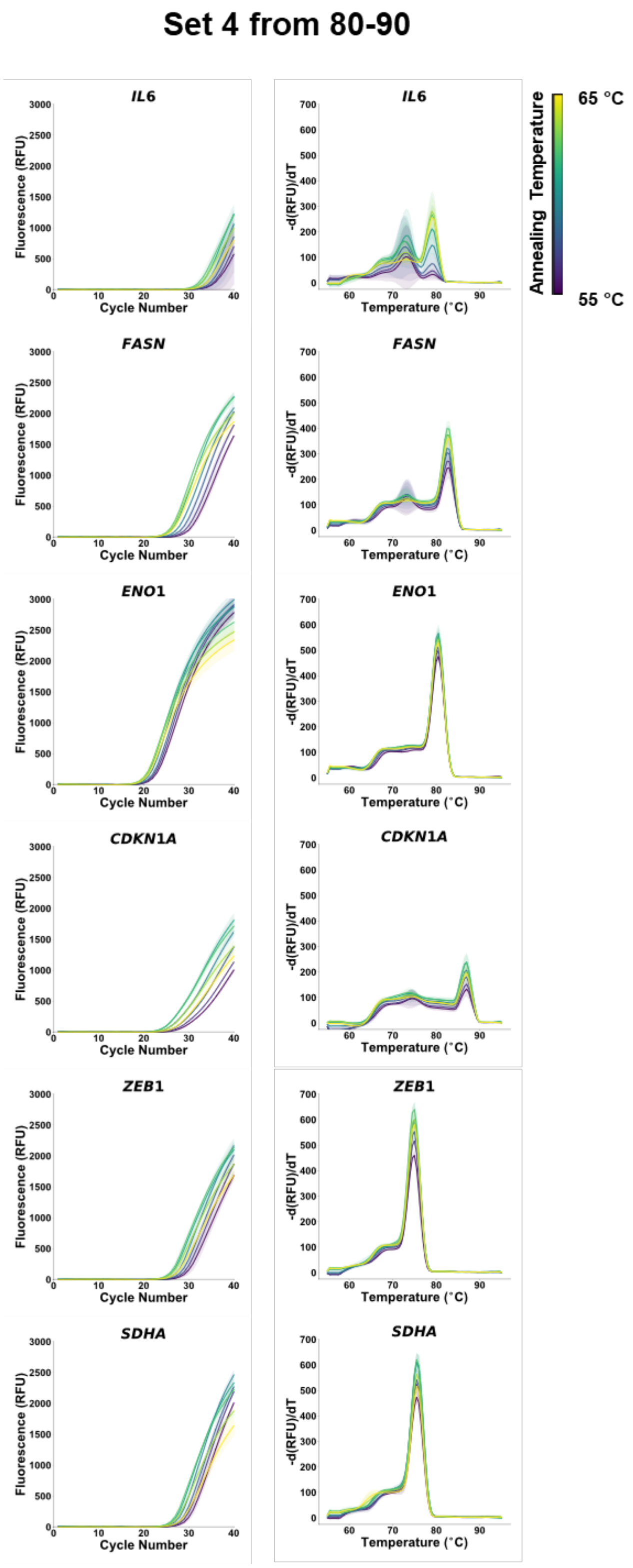


**Supplementary Figure 14. Amplification curves and melt curves for 80-90 bin set 4.** 8 different temperatures were chosen to create amplification curves (left) and melt temperature curves (right). Source data are provided as a Source Data file.

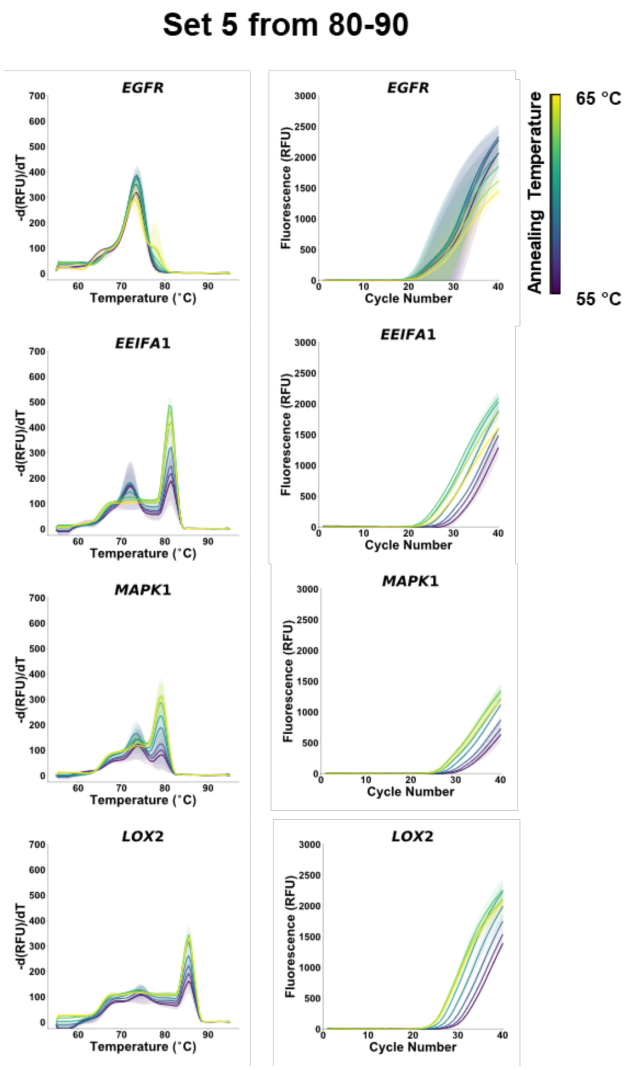


**Supplementary Figure 15. Amplification curves and melt curves for 80-90 bin set 5.** 8 different temperatures were chosen to create amplification curves (left) and melt temperature curves (right). Source data are provided as a Source Data file.

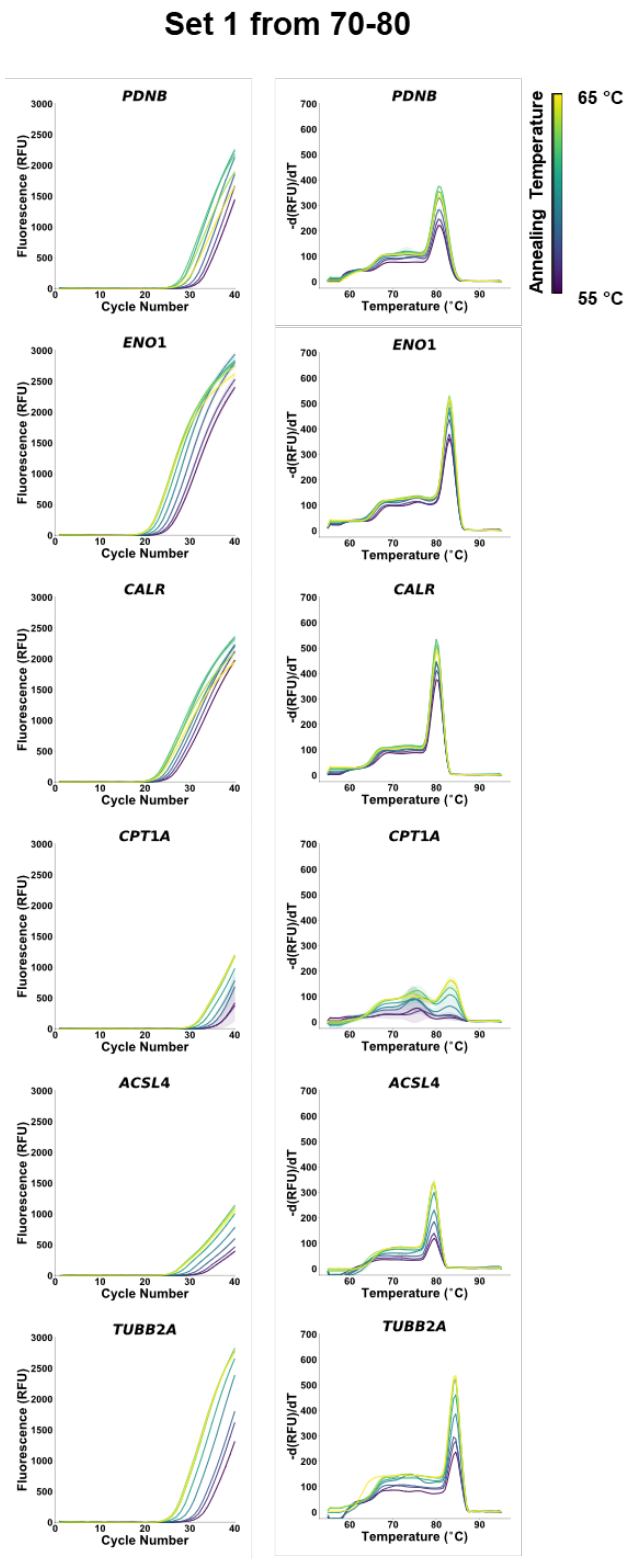


**Supplementary Figure 16. Amplification curves and melt curves for 70-80 bin set 1.** 8 different temperatures were chosen to create amplification curves (left) and melt temperature curves (right). Source data are provided as a Source Data file.

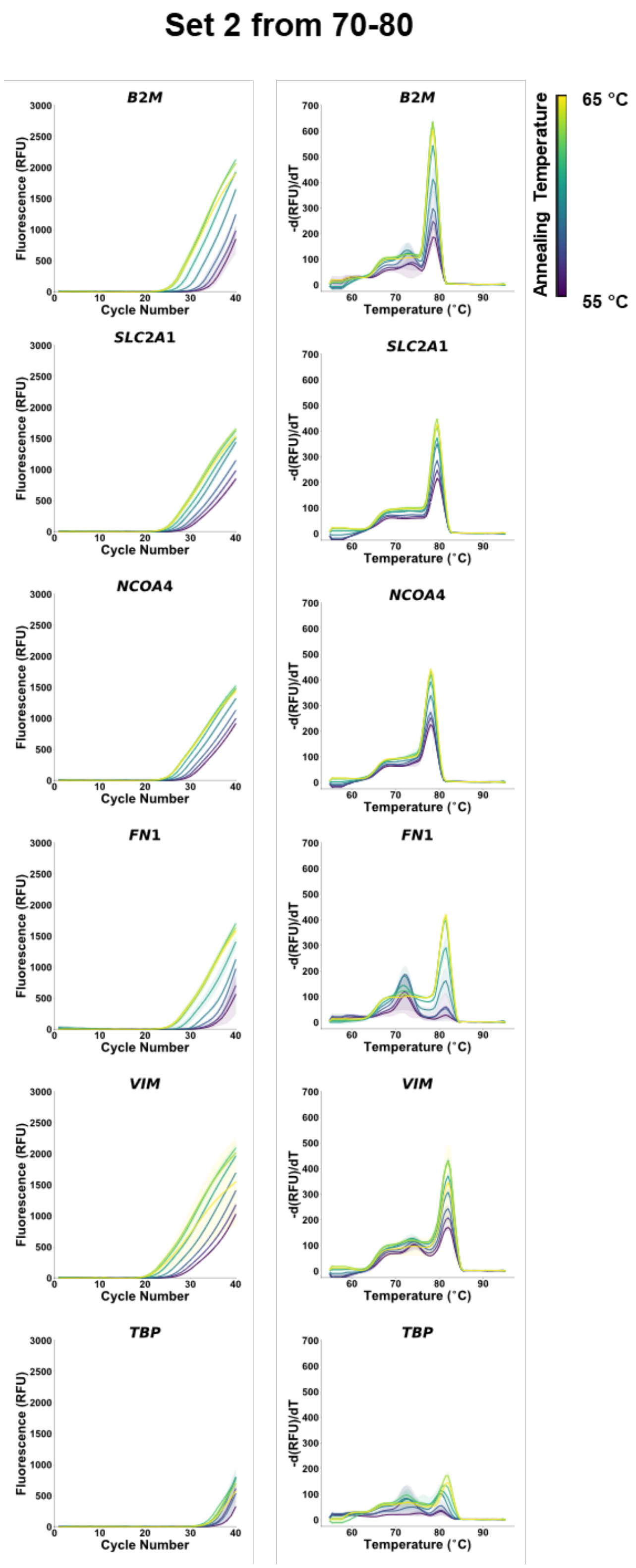


**Supplementary Figure 17. Amplification curves and melt curves for 70-80 bin set 2.** 8 different temperatures were chosen to create amplification curves (left) and melt temperature curves (right). Source data are provided as a Source Data file.

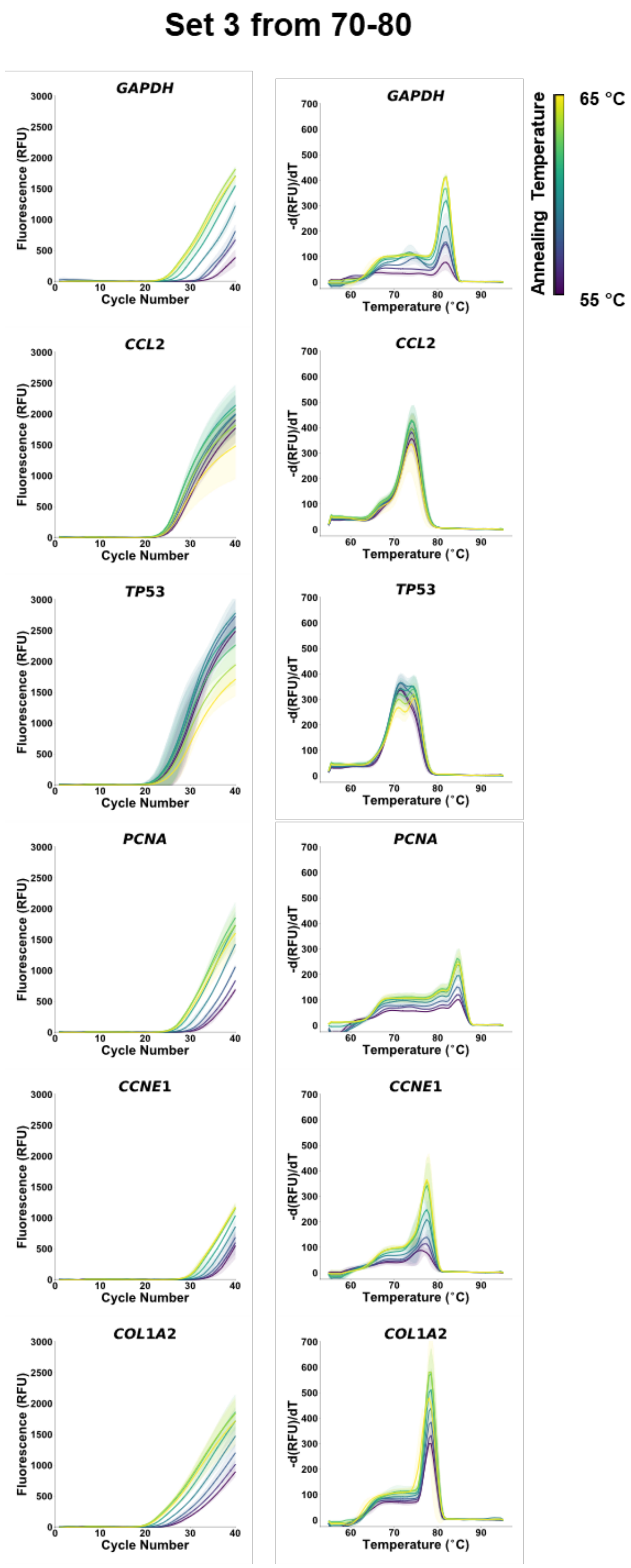


**Supplementary Figure 18. Amplification curves and melt curves for 70-80 bin set 3.** 8 different temperatures were chosen to create amplification curves (left) and melt temperature curves (right). Source data are provided as a Source Data file.

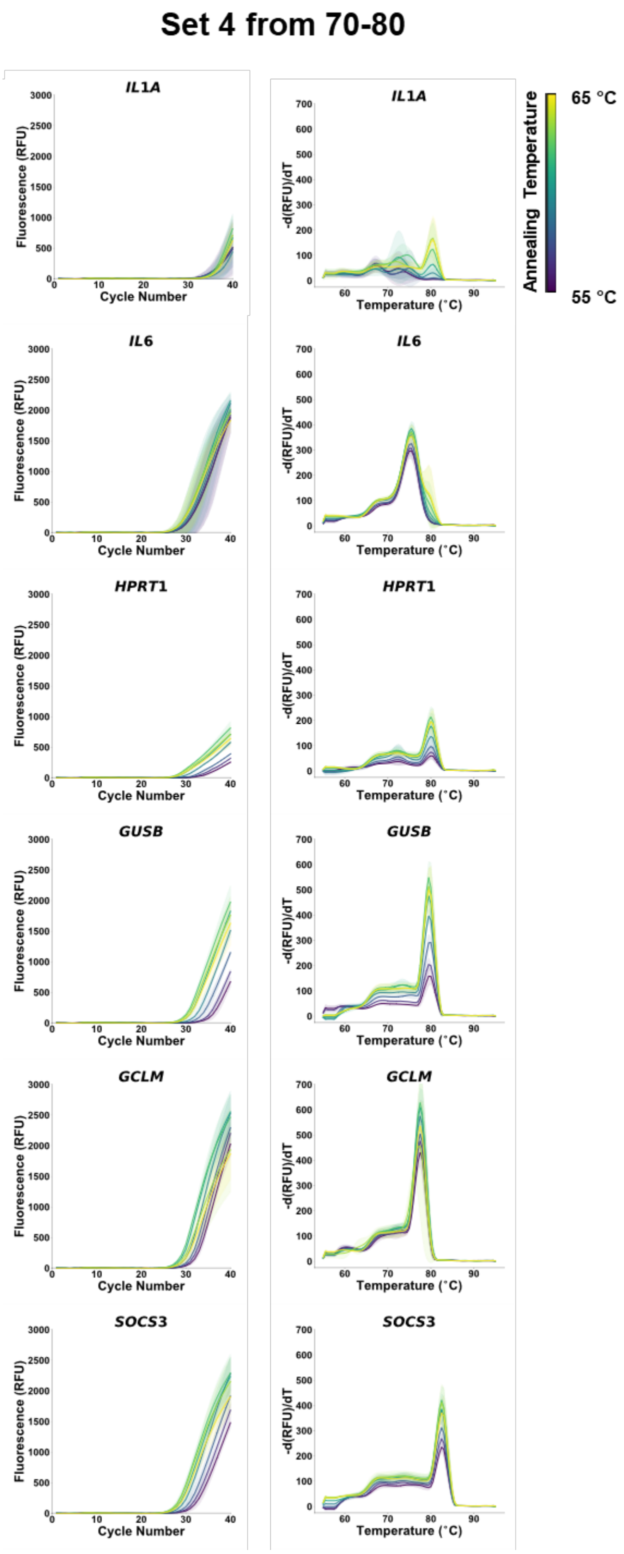


**Supplementary Figure 19. Amplification curves and melt curves for 70-80 bin set 4.** 8 different temperatures were chosen to create amplification curves (left) and melt temperature curves (right). Source data are provided as a Source Data file.

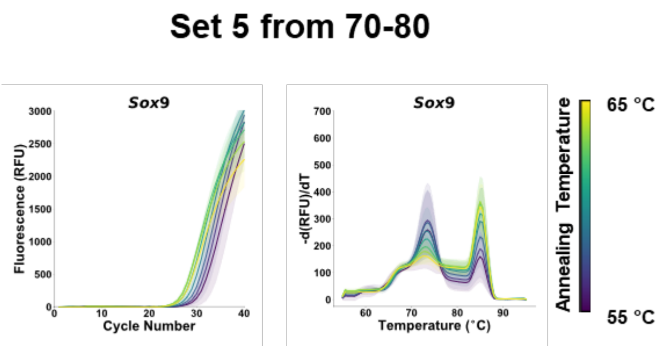


**Supplementary Figure 20. Amplification curves and melt curves for 70-80 bin set 5.** 8 different temperatures were chosen to create amplification curves (left) and melt temperature curves (right). Source data are provided as a Source Data file.

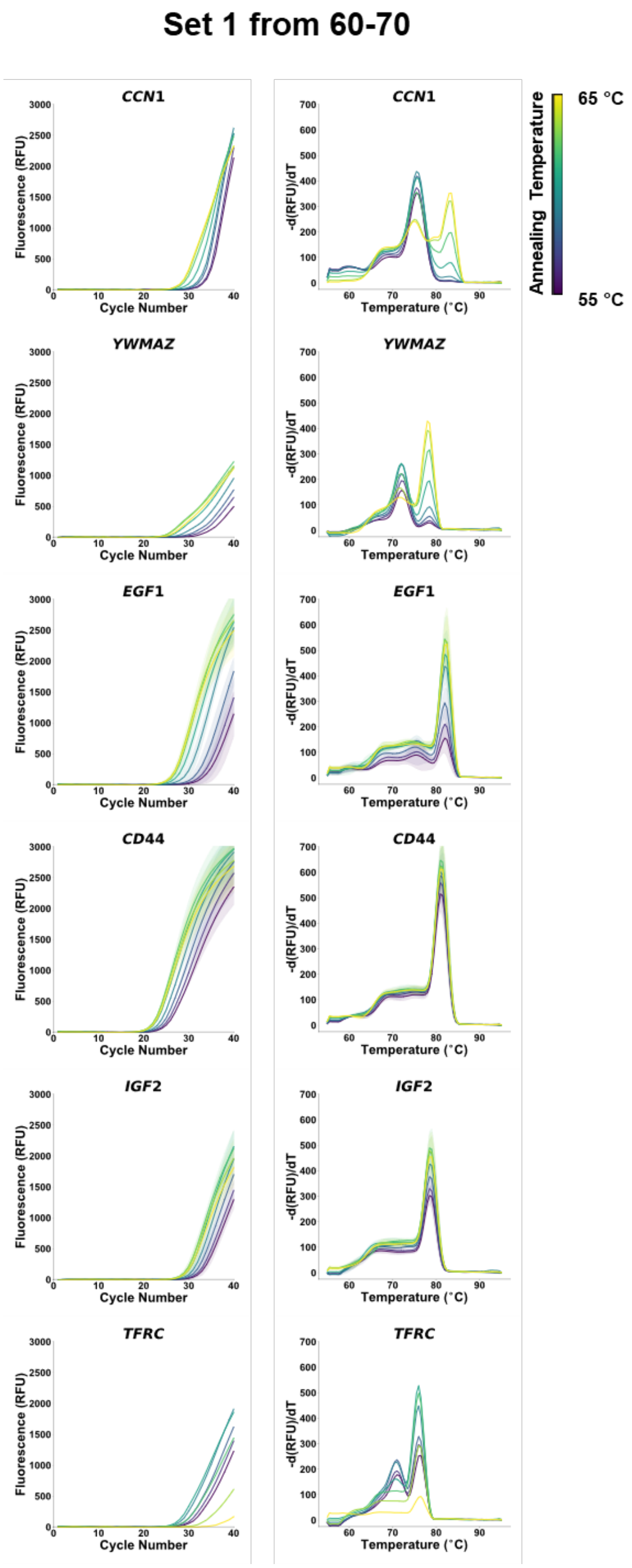


**Supplementary Figure 21. Amplification curves and melt curves for 60-70 bin set 1.** 8 different temperatures were chosen to create amplification curves (left) and melt temperature curves (right). Source data are provided as a Source Data file.

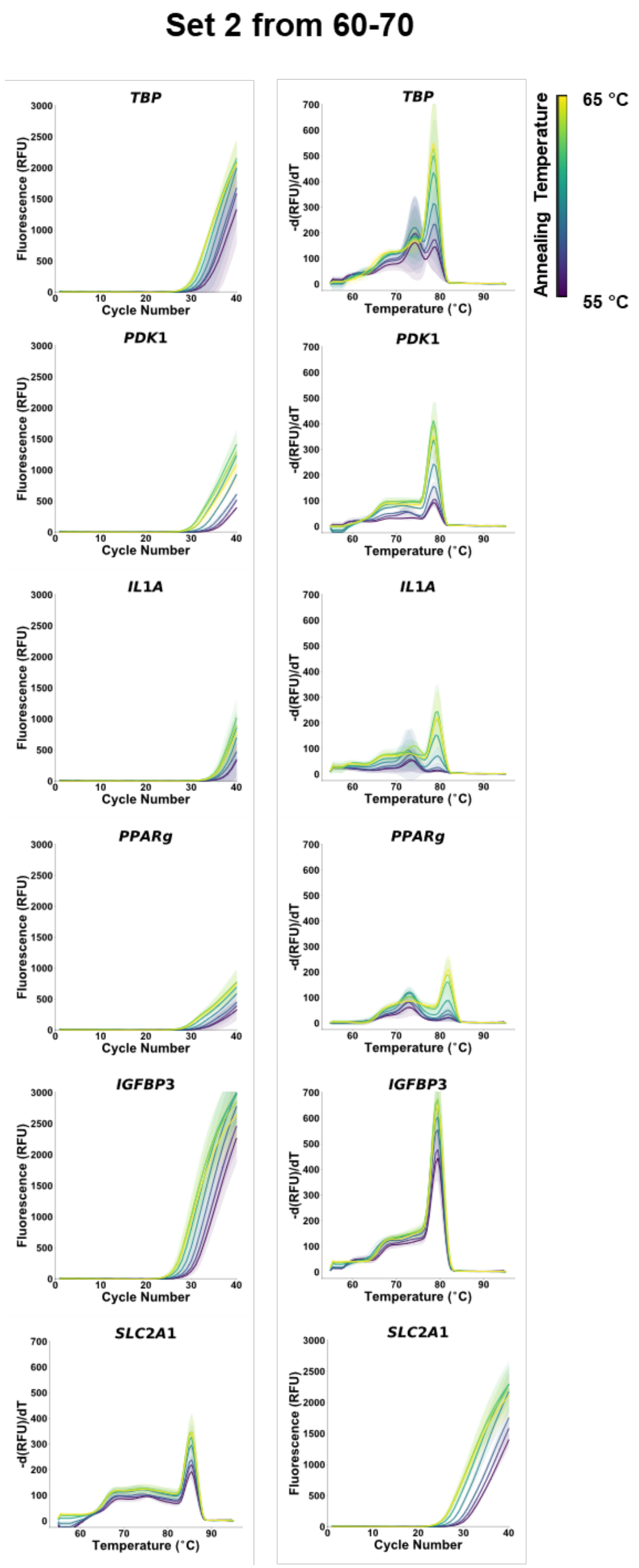


**Supplementary Figure 22. Amplification curves and melt curves for 60-70 bin set 2.** 8 different temperatures were chosen to create amplification curves (left) and melt temperature curves (right). Source data are provided as a Source Data file.

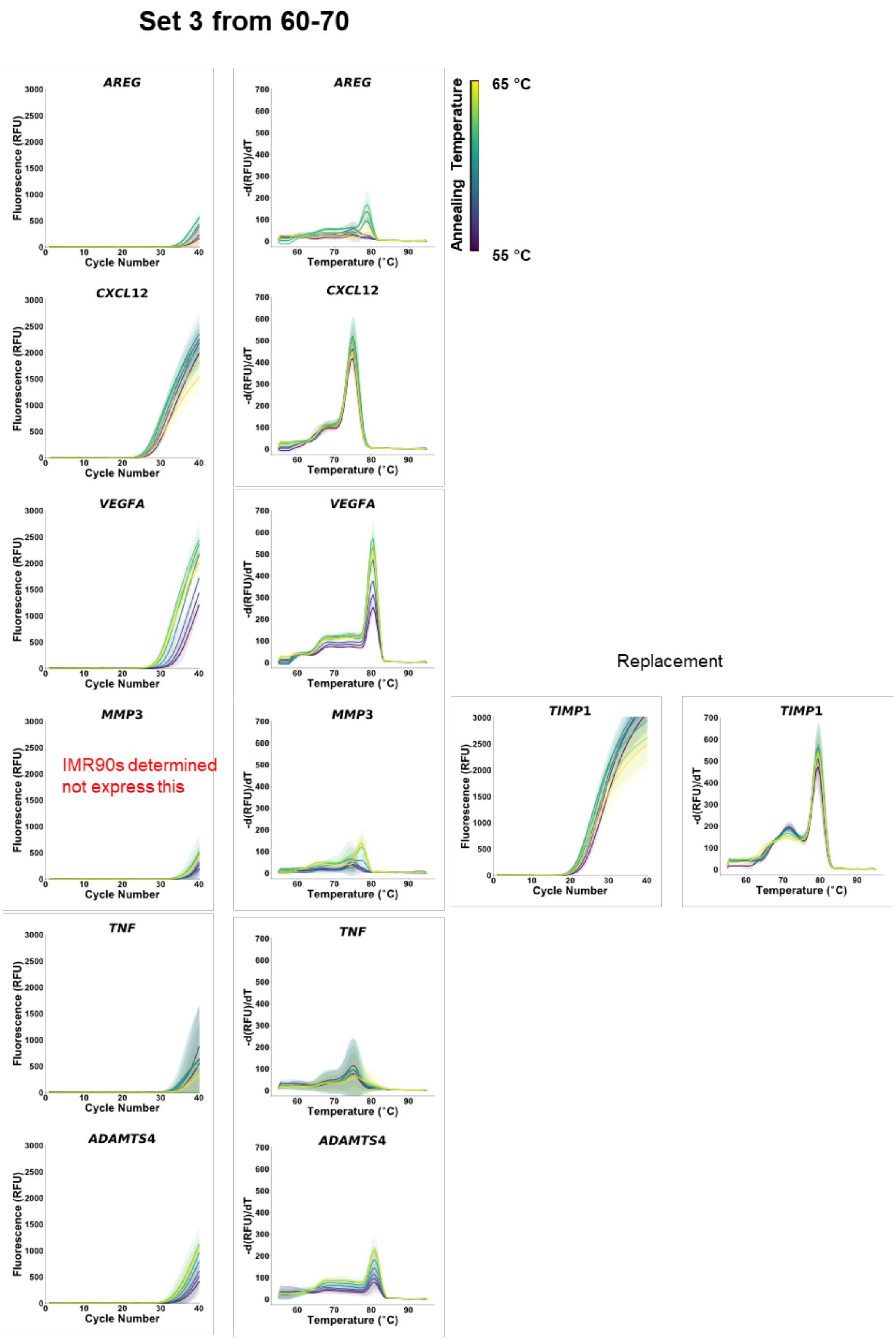


**Supplementary Figure 23. Amplification curves and melt curves for 60-70 bin set 3.** 8 different temperatures were chosen to create amplification curves (left) and melt temperature curves (right). Source data are provided as a Source Data file.

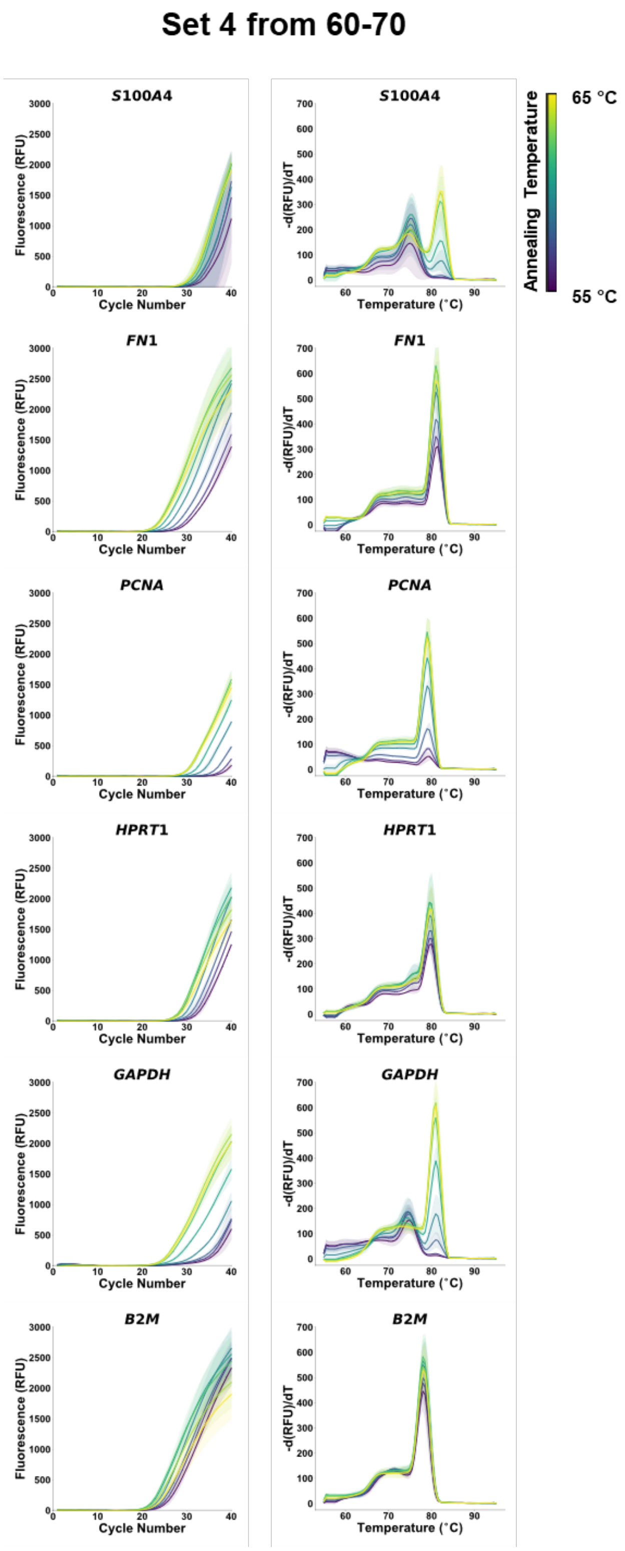


**Supplementary Figure 24. Amplification curves and melt curves for 60-70 bin set 4.** 8 different temperatures were chosen to create amplification curves (left) and melt temperature curves (right). Source data are provided as a Source Data file.

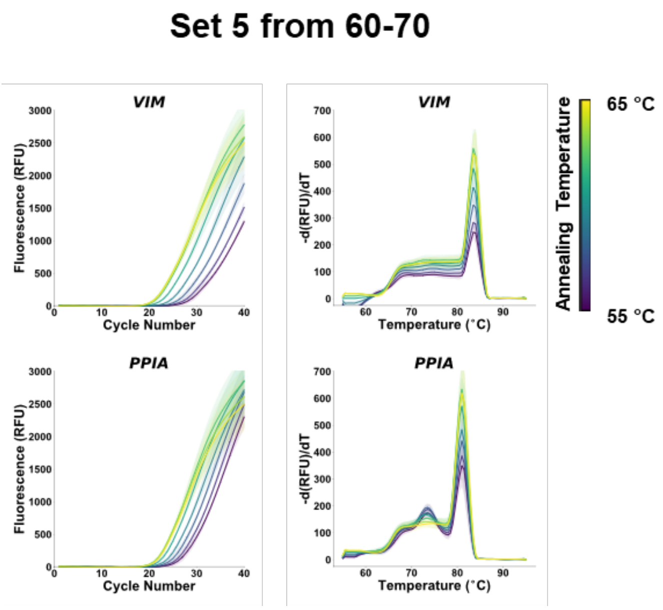


**Supplementary Figure 25. Amplification curves and melt curves for 60-70 bin set 5.** 8 different temperatures were chosen to create amplification curves (left) and melt temperature curves (right). Source data are provided as a Source Data file.

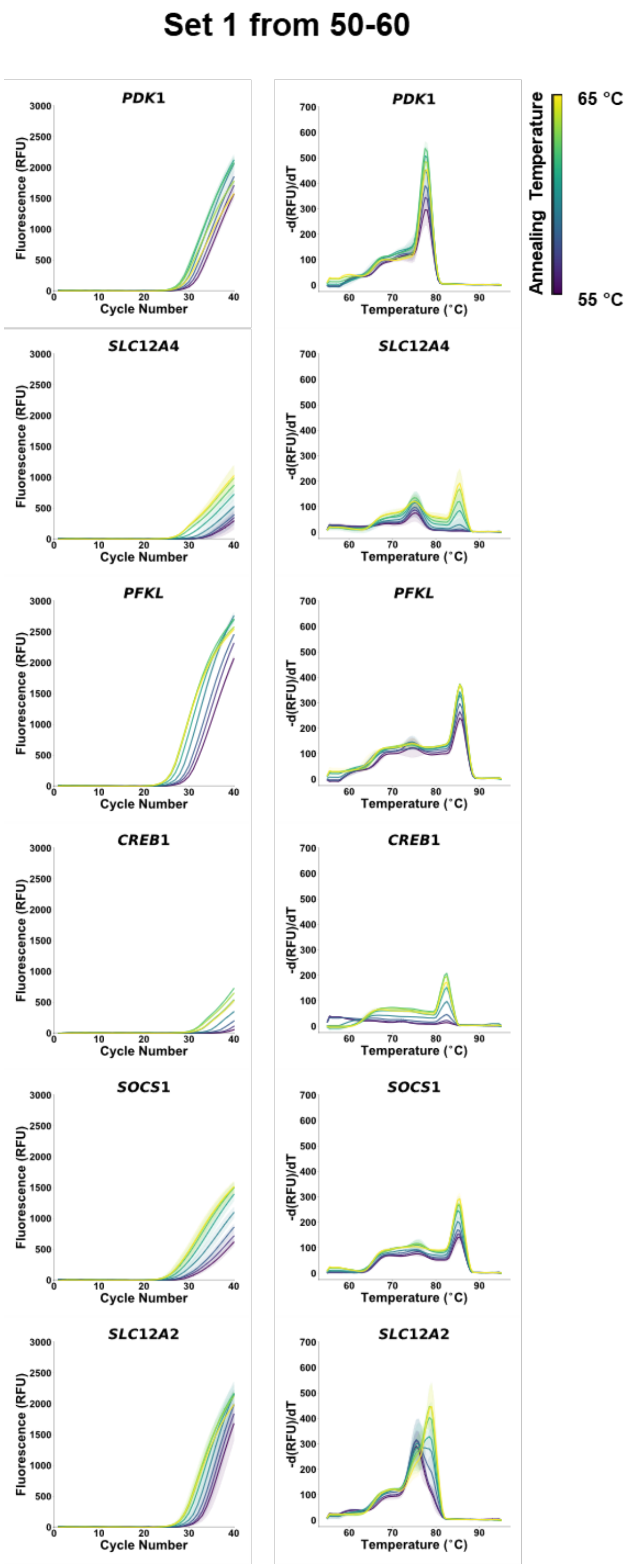


**Supplementary Figure 26. Amplification curves and melt curves for 50-60 bin set 1.** 8 different temperatures were chosen to create amplification curves (left) and melt temperature curves (right). Source data are provided as a Source Data file.

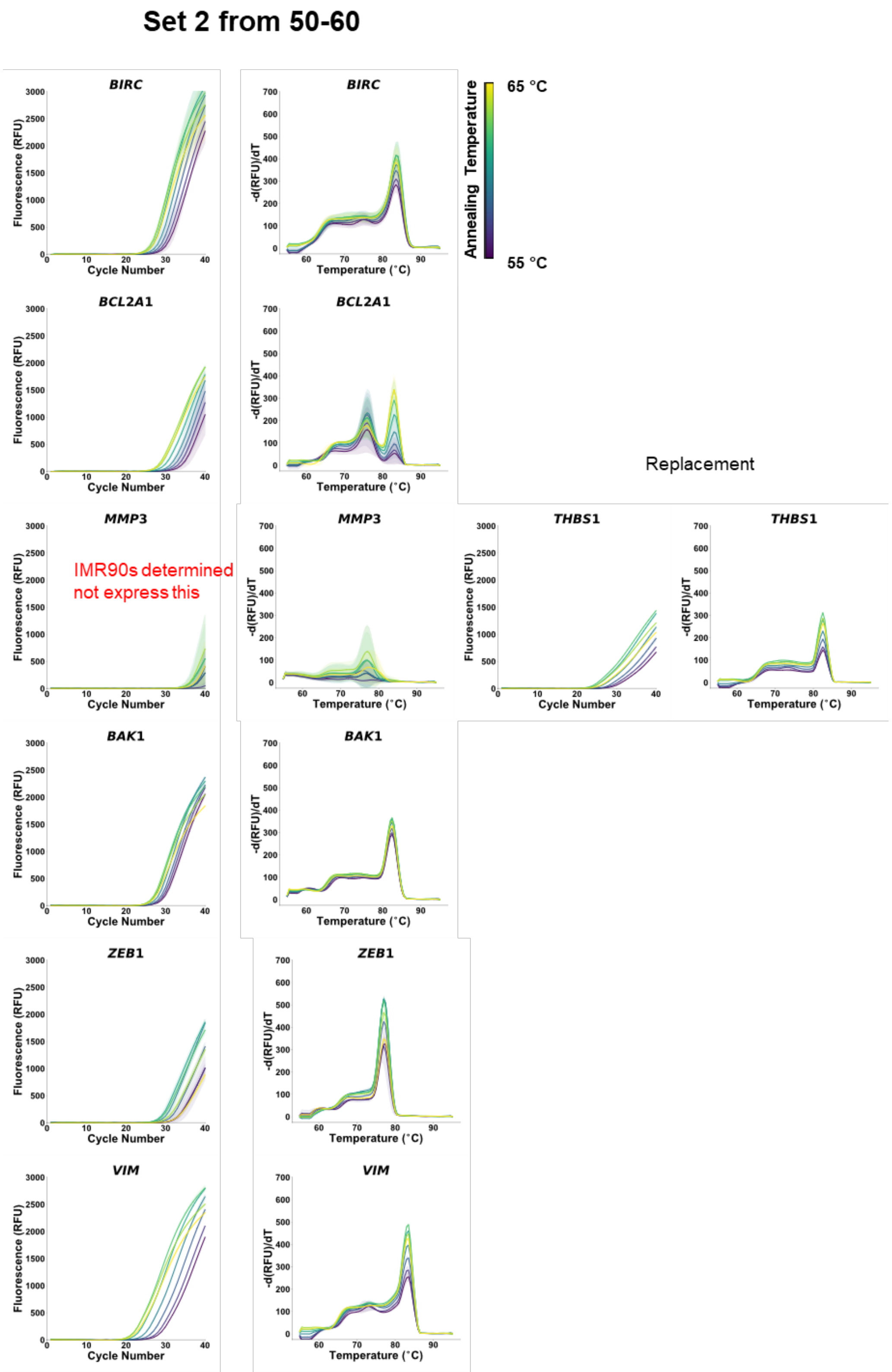


**Supplementary Figure 27. Amplification curves and melt curves for 50-60 bin set 2.** 8 different temperatures were chosen to create amplification curves (left) and melt temperature curves (right). Source data are provided as a Source Data file.

.

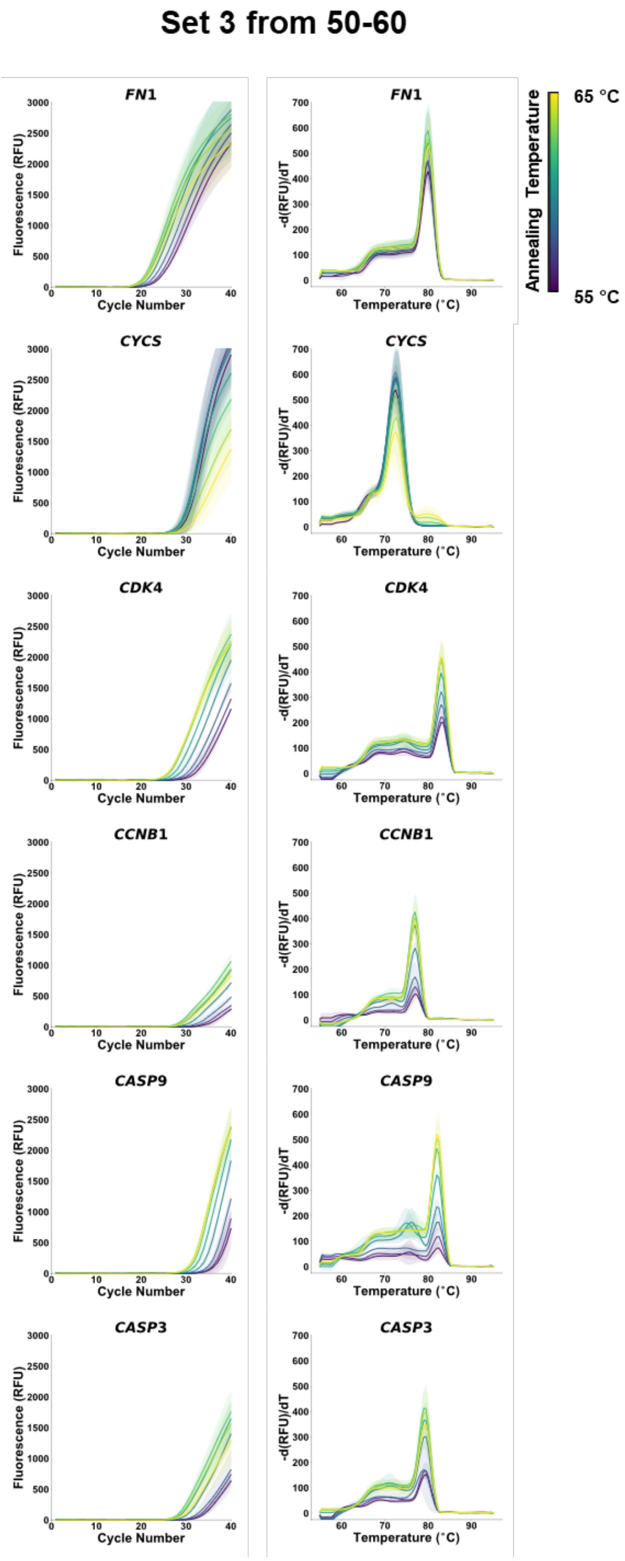


**Supplementary Figure 28. Amplification curves and melt curves for 50-60 bin set 3.** 8 different temperatures were chosen to create amplification curves (left) and melt temperature curves (right). Source data are provided as a Source Data file.

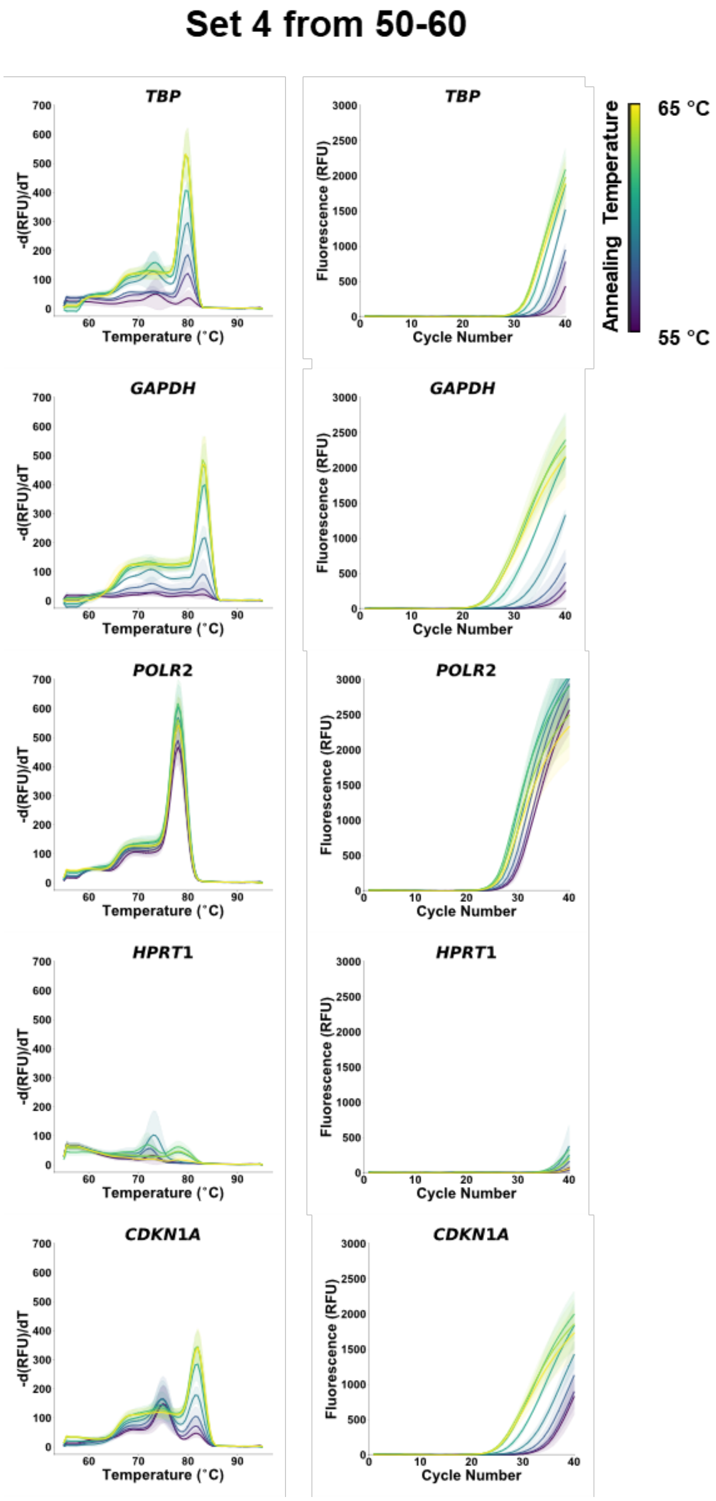


**Supplementary Figure 29. Amplification curves and melt curves for 50-60 bin set 4.** 8 different temperatures were chosen to create amplification curves (left) and melt temperature curves (right). Source data are provided as a Source Data file.

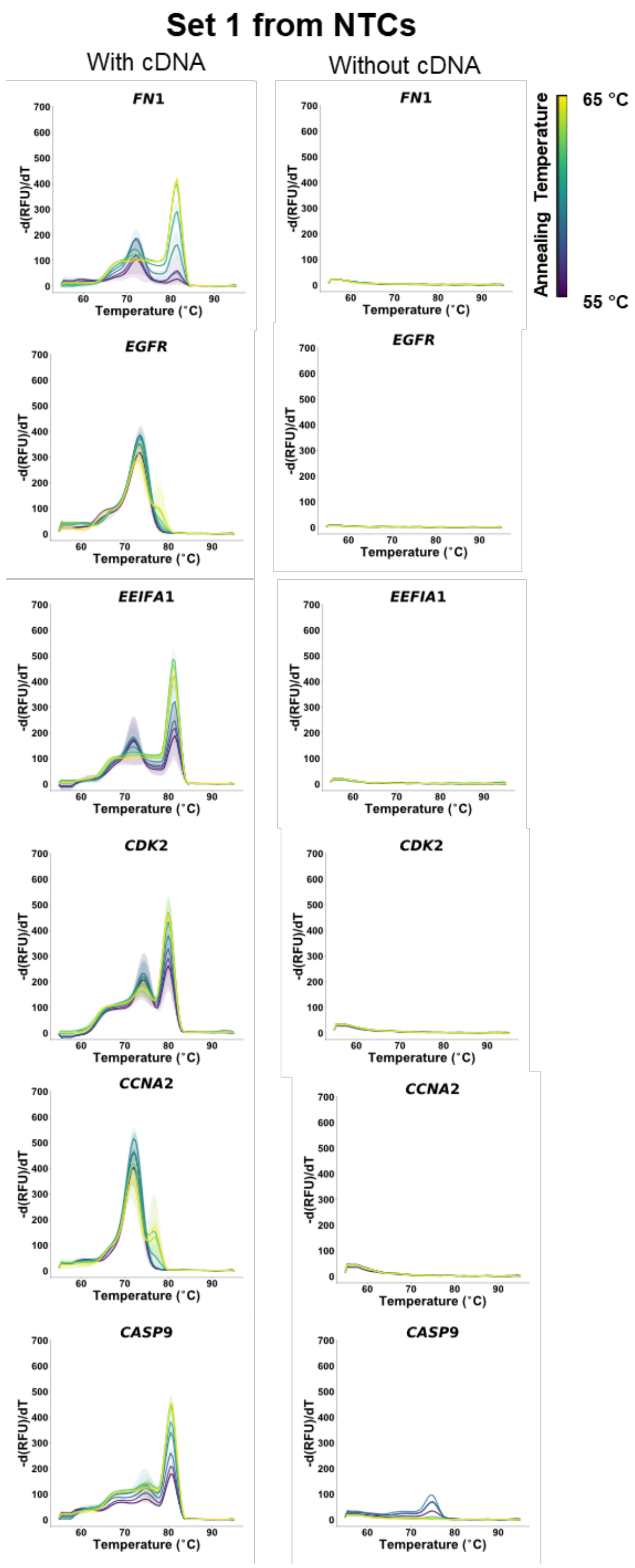


**Supplementary Figure 30. Melt curves for cDNA and NTCs of double peak genes set 1.** 8 different temperatures were chosen to create melt temperature curves with (left) and without cDNA template. Source data are provided as a Source Data file.

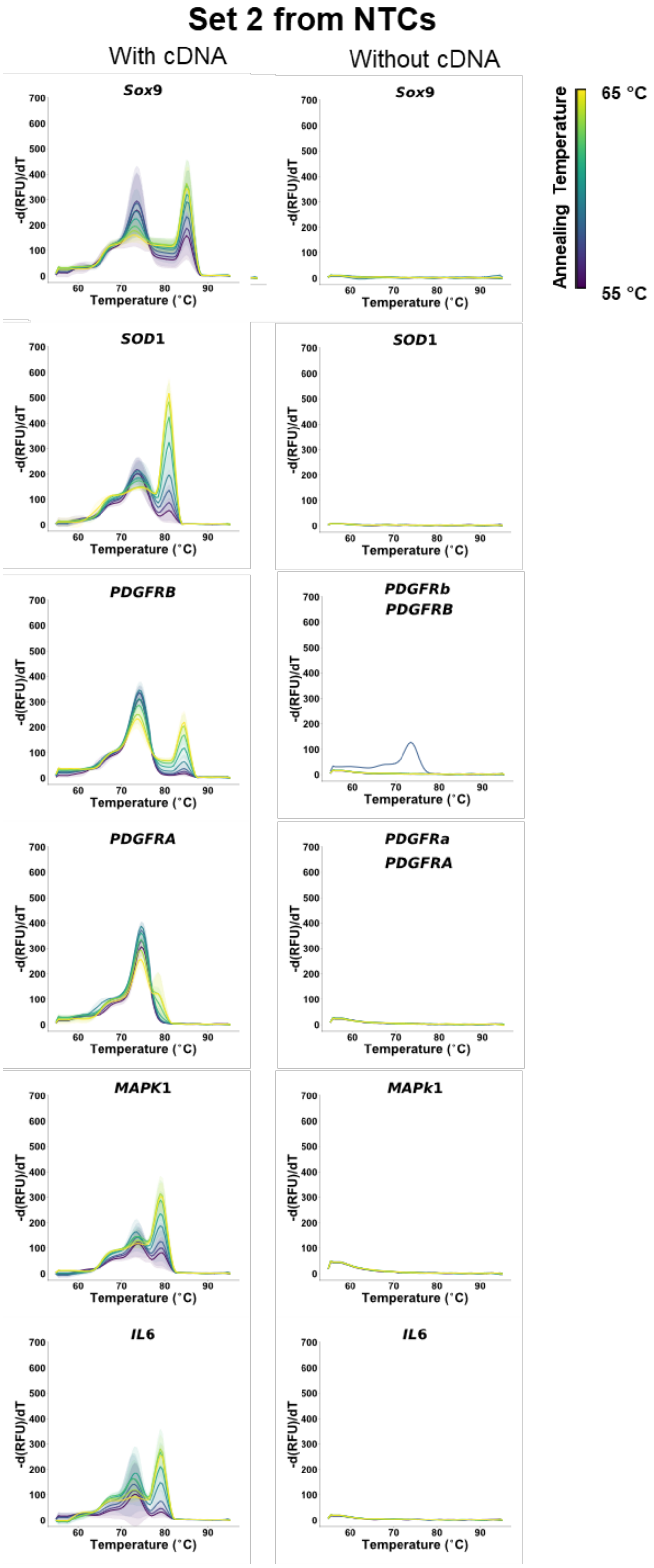


**Supplementary Figure 31. Melt curves for cDNA and NTCs of double peak genes set 2.** 8 different temperatures were chosen to create melt temperature curves with (left) and without cDNA template. Source data are provided as a Source Data file.

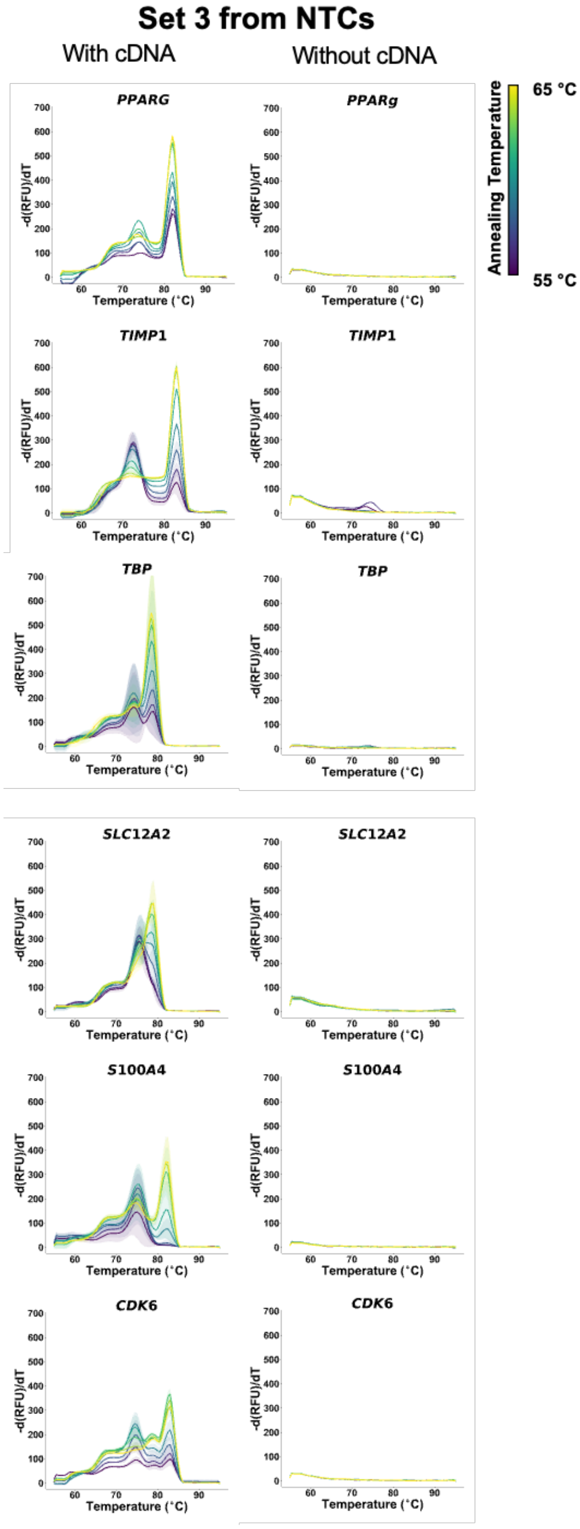


**Supplementary Figure 32. Melt curves for cDNA and NTCs of double peak genes set 3.** 8 different temperatures were chosen to create melt temperature curves with (left) and without cDNA template. Source data are provided as a Source Data file.

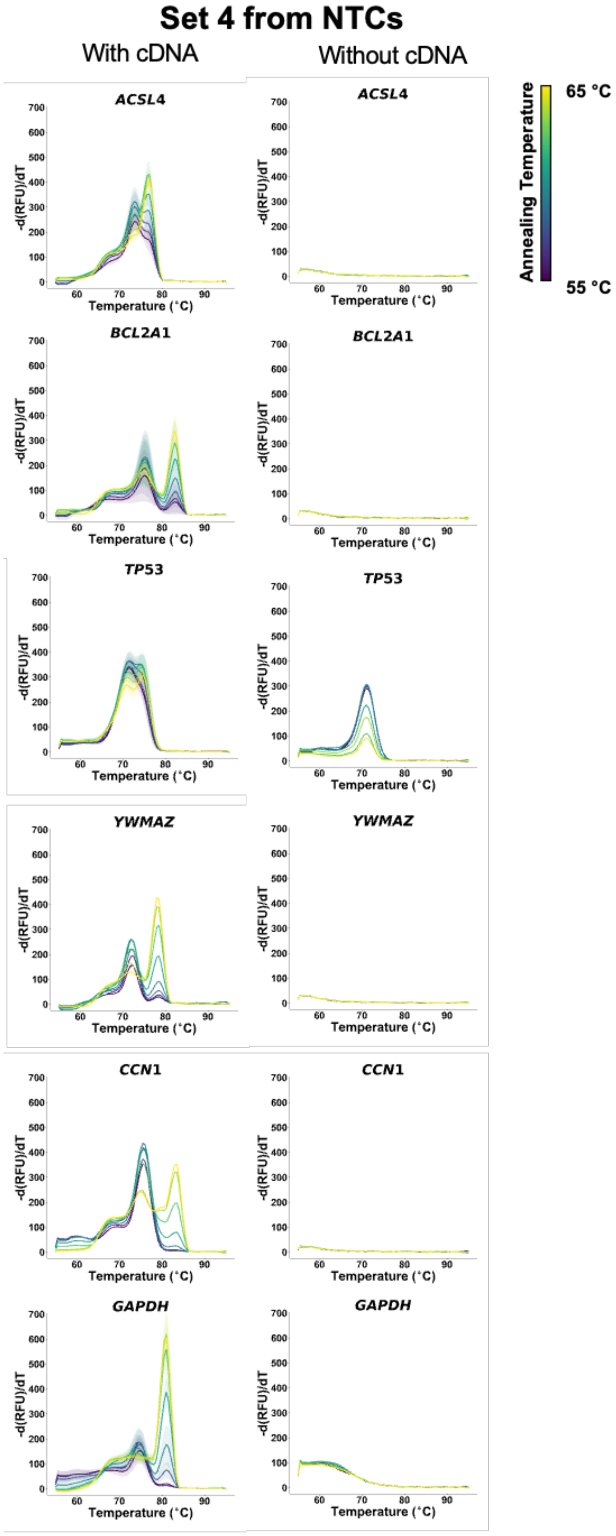


**Supplementary Figure 33. Melt curves for cDNA and NTCs of double peak genes set 4.** 8 different temperatures were chosen to create melt temperature curves with (left) and without cDNA template. Source data are provided as a Source Data file.

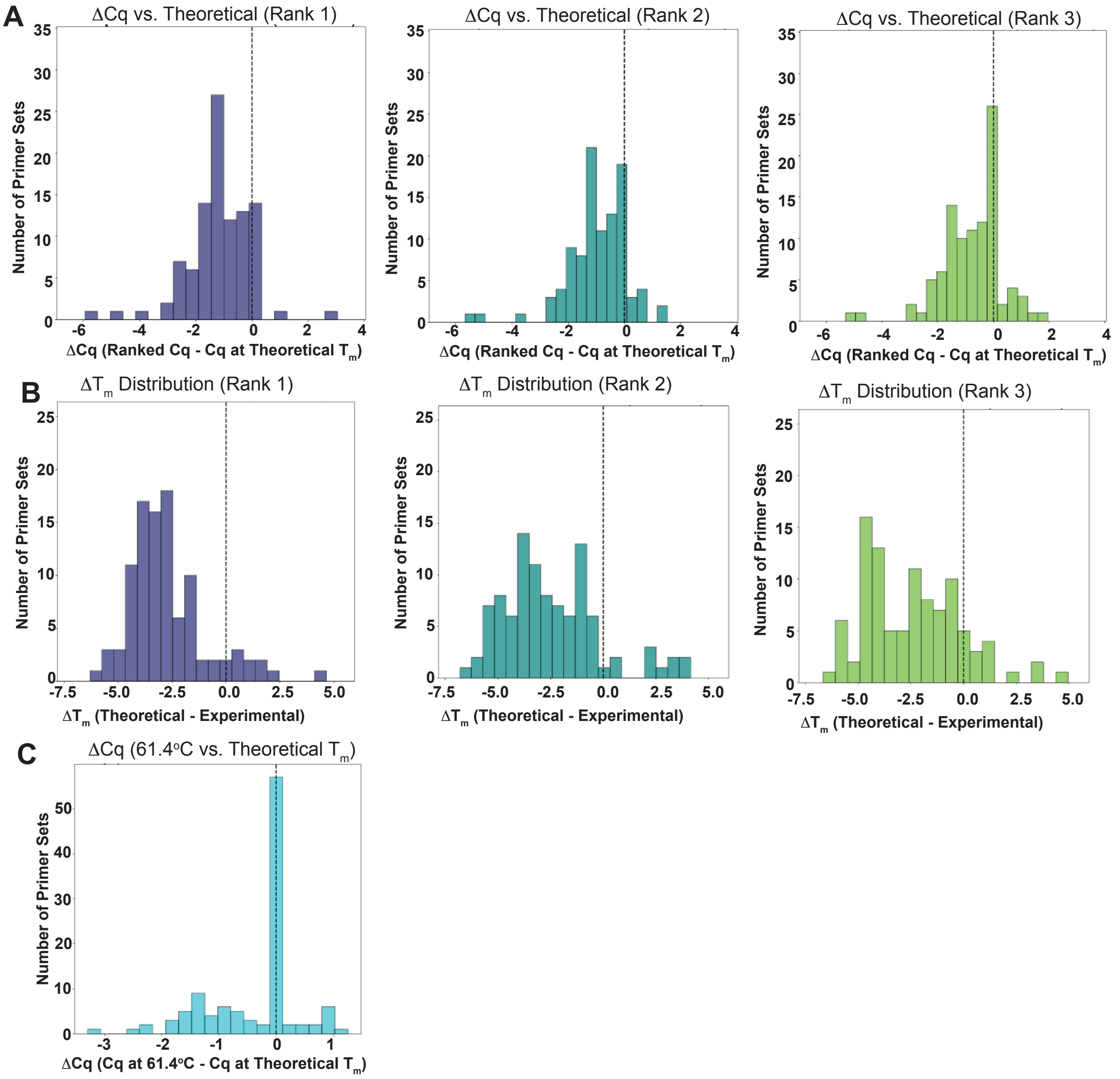


**Supplementary Figure 34. Ranking of optimal melt temps. (A)** the difference in CT value from the top three ranked melt temperatures as compared to the theoretical. **(B)** the difference in melt temperature from the top three ranked melt temperatures as compared to the theoretical. **(C)** Difference in theoretical melt temperatures for each primer pair as compared to 61.4 °C. Sample sizes are **(A)** n = 132, 132, and 132 for ranks 1, 2, and 3, respectively. Sample sizes are **(B)** n = 132, 132, and 132 for ranks 1, 2, and 3, respectively. Sample size in **(C)** n = 132. Source data are provided as a Source Data file.

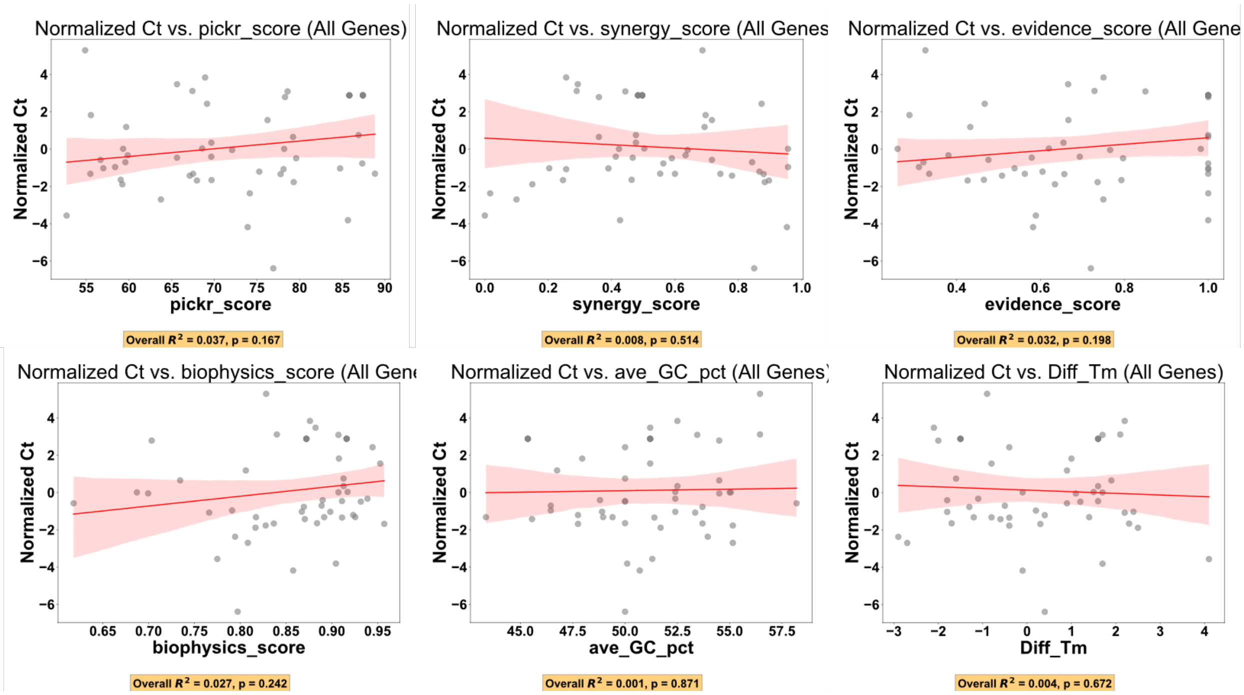


**Supplementary Figure 35. CT versus score sub-types.** Scatter plots of various scores and primer pair characteristics against normalized CT difference between a primer pair for a gene and the best CT for a pair for the same gene. Sample sizes n=112 for all the comparisons. Zero values were removed from the graphs. Source data are provided as a Source Data file. The code used in the creation of these data are published under a CC-BY-NC-ND license (https://creativecommons.org/licenses/by-nc-nd/4.0/deed.en).

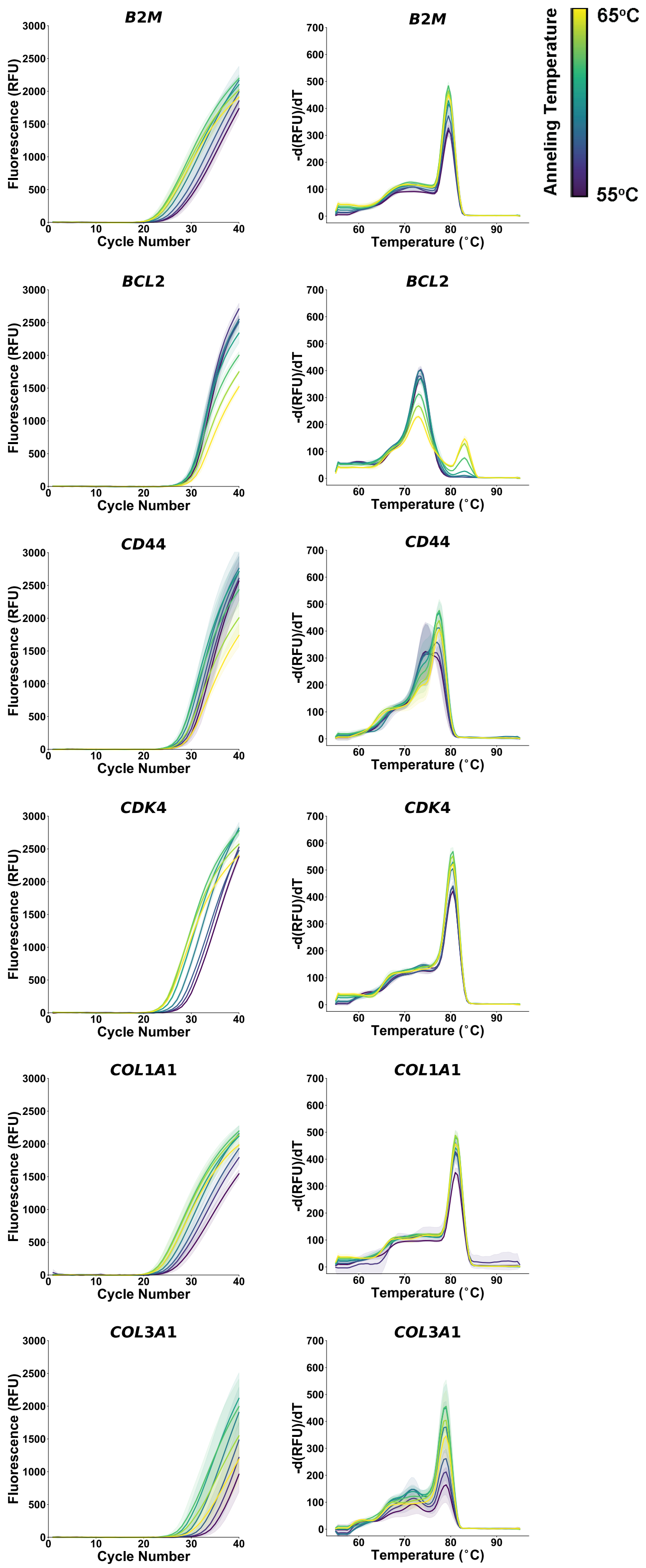


**Supplementary Figure 36. Amplification curves and melt curves for 80-100 score mouse primers.** 8 different temperatures were chosen to create amplification curves (left) and melt temperature curves (right). Source data are provided as a Source Data file.

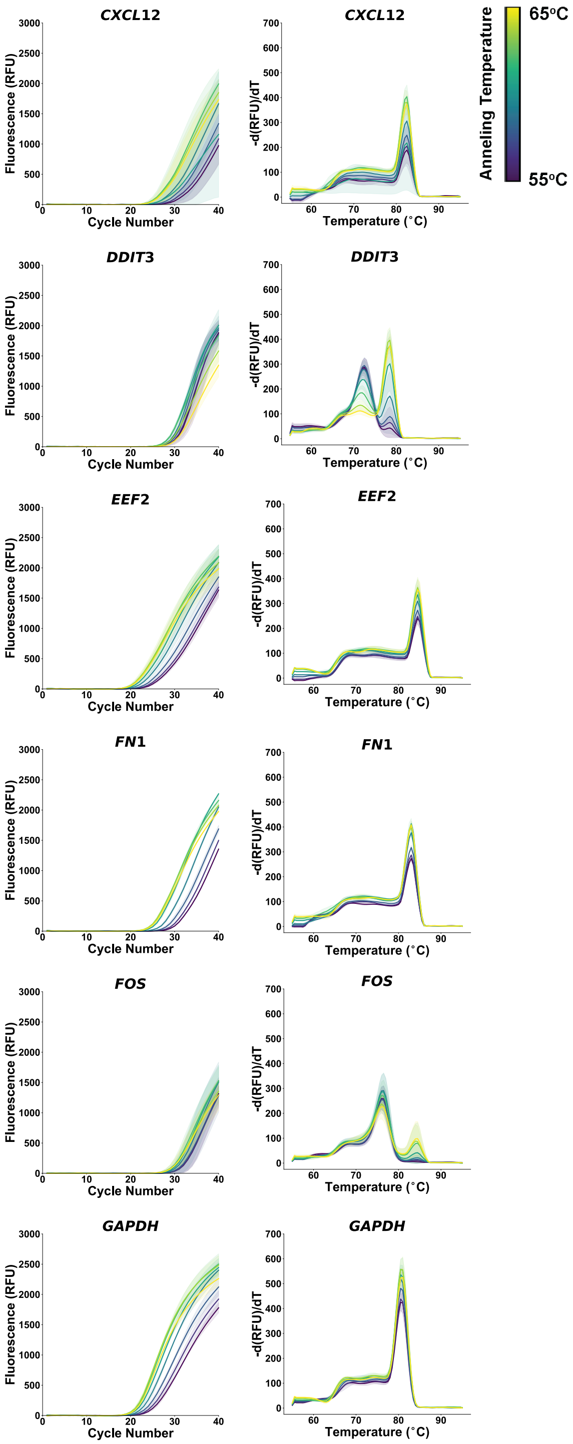


**Supplementary Figure 37. Amplification curves and melt curves for 80-100 score mouse primers.** 8 different temperatures were chosen to create amplification curves (left) and melt temperature curves (right). Source data are provided as a Source Data file.

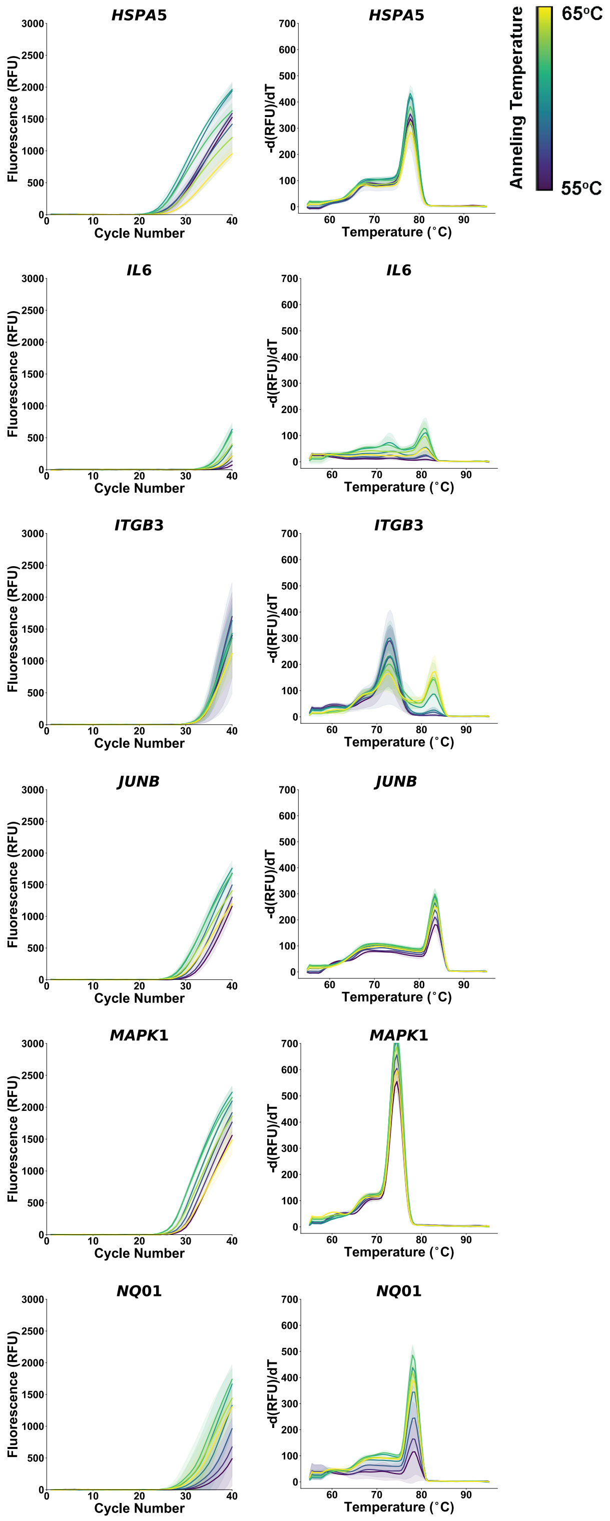


**Supplementary Figure 38. Amplification curves and melt curves for 80-100 score mouse primers.** 8 different temperatures were chosen to create amplification curves (left) and melt temperature curves (right). Source data are provided as a Source Data file.

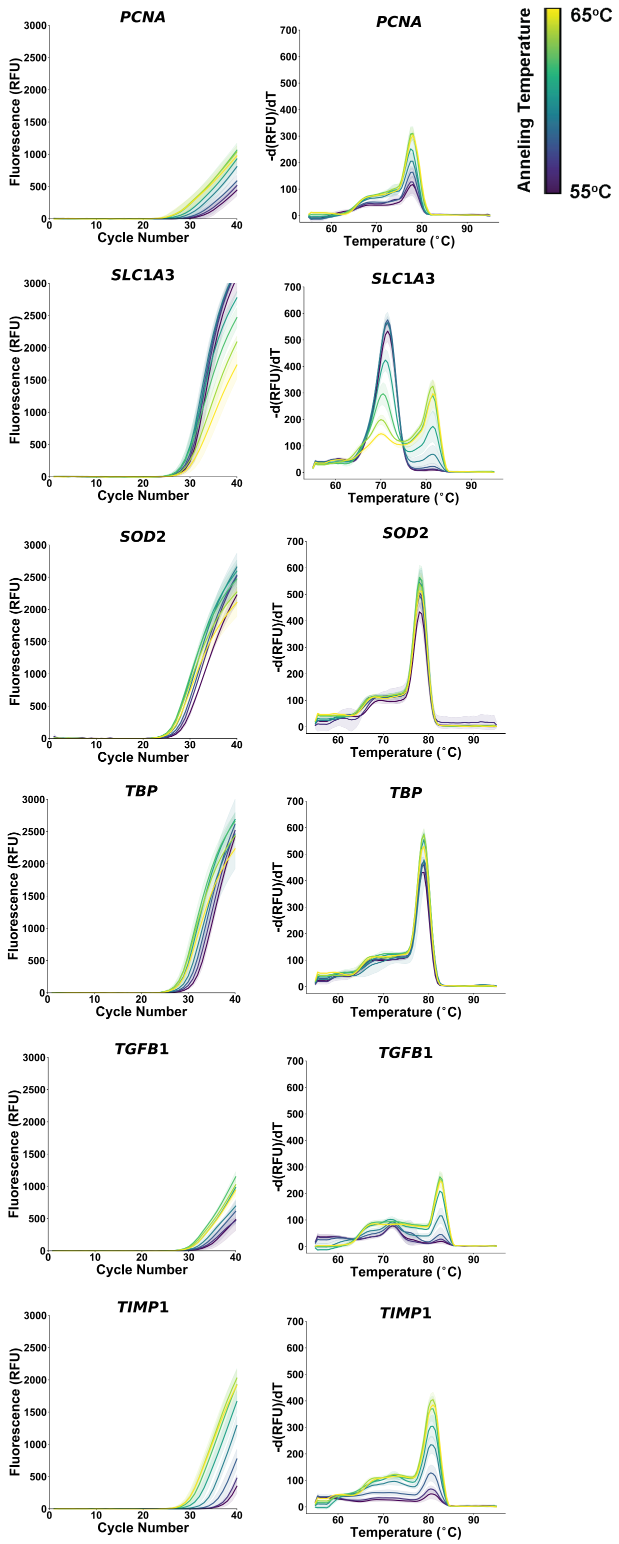


**Supplementary Figure 39. Amplification curves and melt curves for 80-100 score mouse primers.** 8 different temperatures were chosen to create amplification curves (left) and melt temperature curves (right). Source data are provided as a Source Data file.

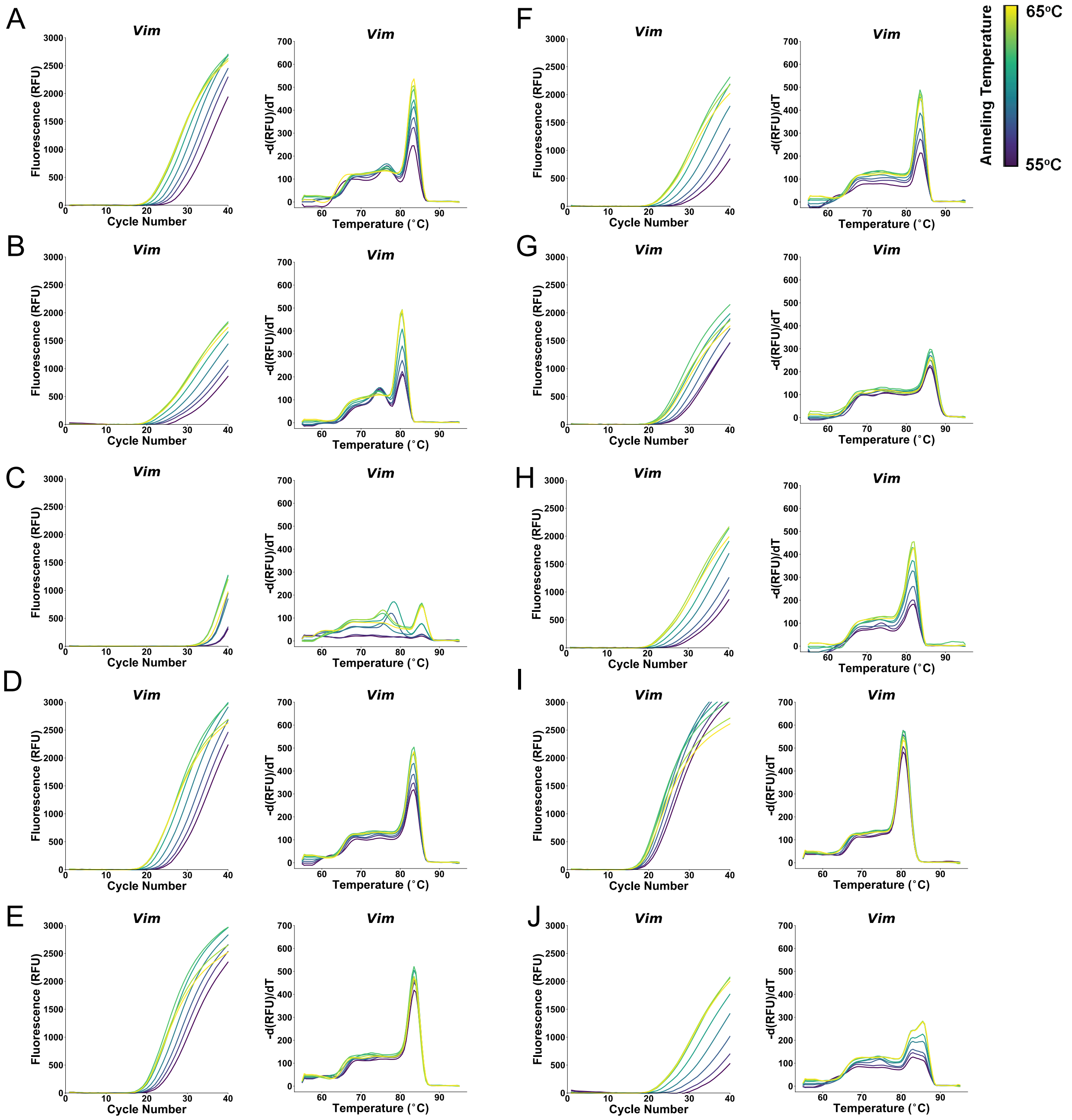


**Supplementary Figure 40. Amplification curves and melt curves for 10 primer pairs generated for human Vimentin (VIM) using Primer Blast.** 8 different temperatures were chosen to create amplification curves (left) and melt temperature curves (right). Source data are provided as a Source Data file.

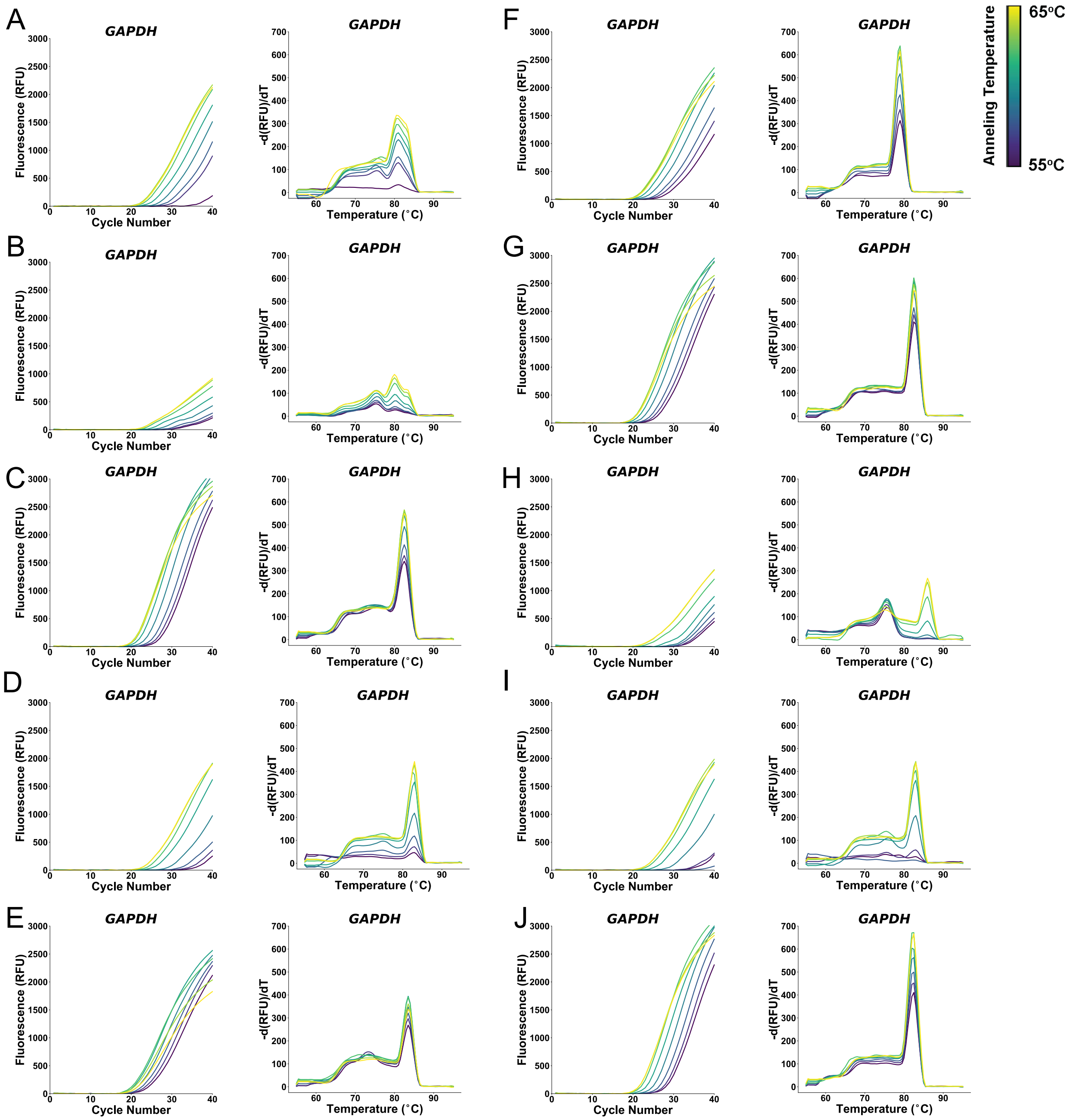


**Supplementary Figure 41. Amplification curves and melt curves for 10 primer pairs generated for human GAPDH using Primer Blast.** 8 different temperatures were chosen to create amplification curves (left) and melt temperature curves (right). Source data are provided as a Source Data file.

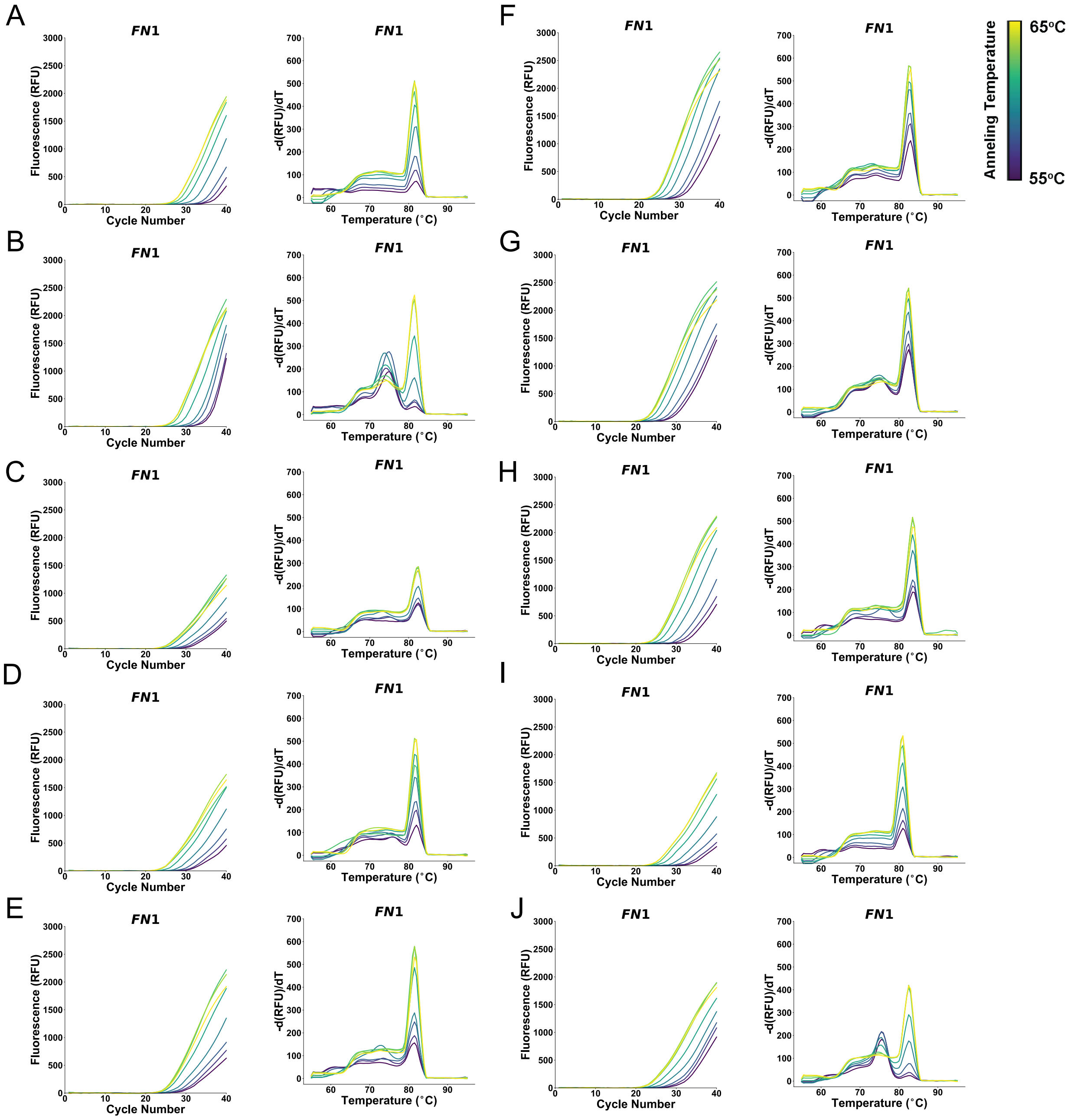


**Supplementary Figure 42. Amplification curves and melt curves for 10 primer pairs generated for human Fibronectin 1 (FN1) using Primer Blast.** 8 different temperatures were chosen to create amplification curves (left) and melt temperature curves (right). Source data are provided as a Source Data file.

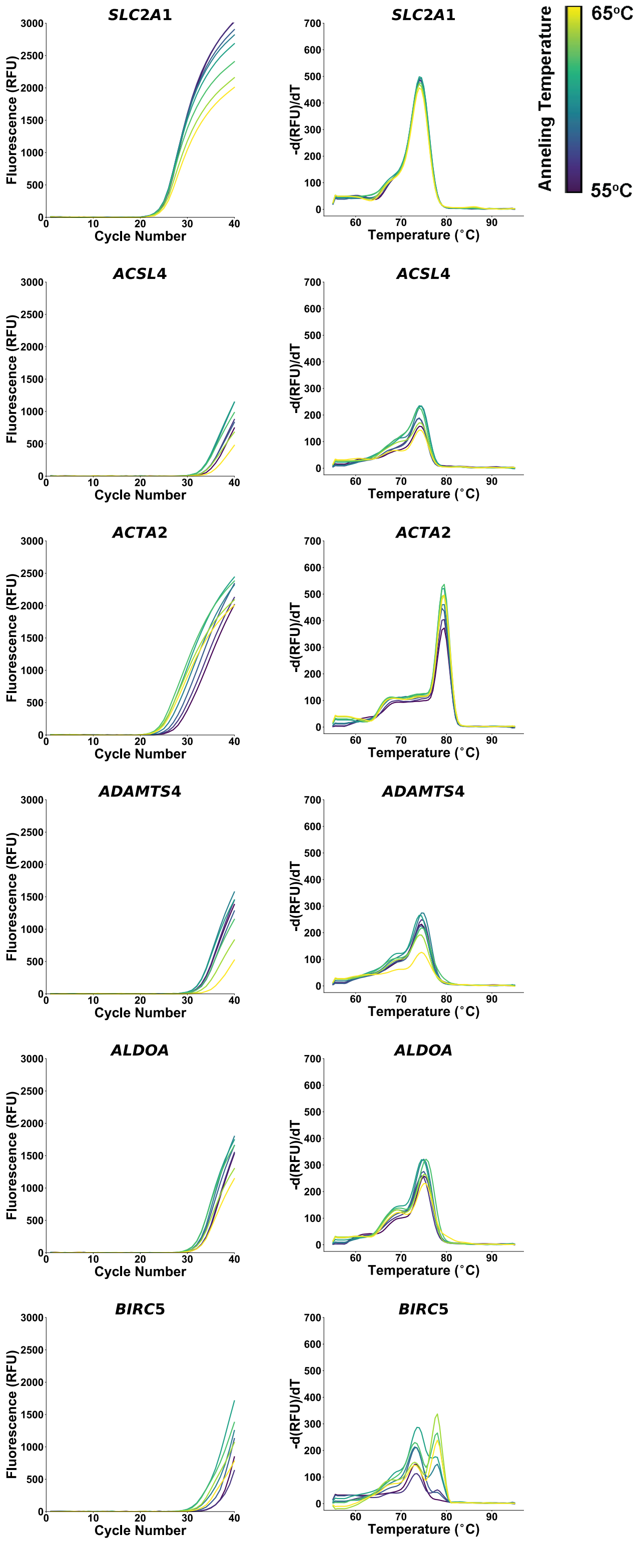


**Supplementary Figure 43. Amplification curves and melt curves for primer pairs generated using Primer Blast.** 8 different temperatures were chosen to create amplification curves (left) and melt temperature curves (right). Source data are provided as a Source Data file.

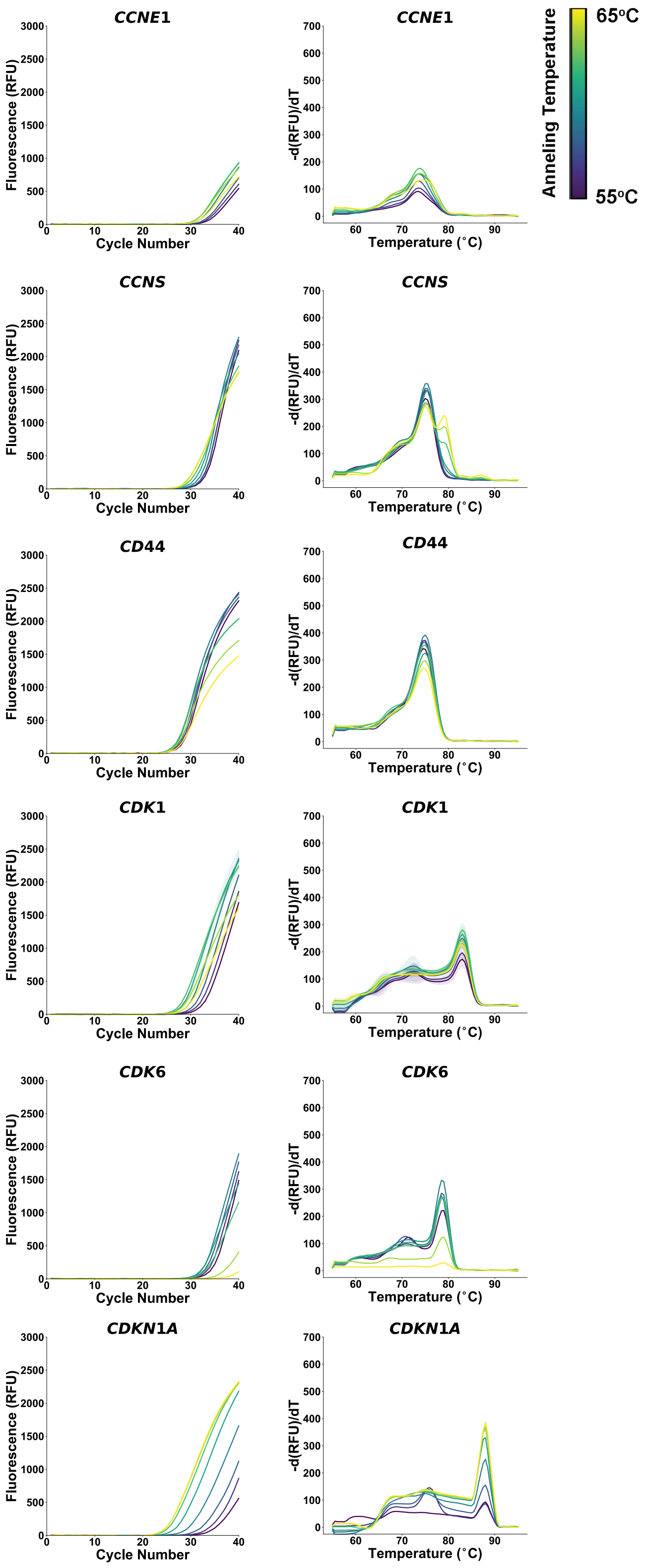


**Supplementary Figure 44. Amplification curves and melt curves for primer pairs generated using Primer Blast.** 8 different temperatures were chosen to create amplification curves (left) and melt temperature curves (right). Source data are provided as a Source Data file.

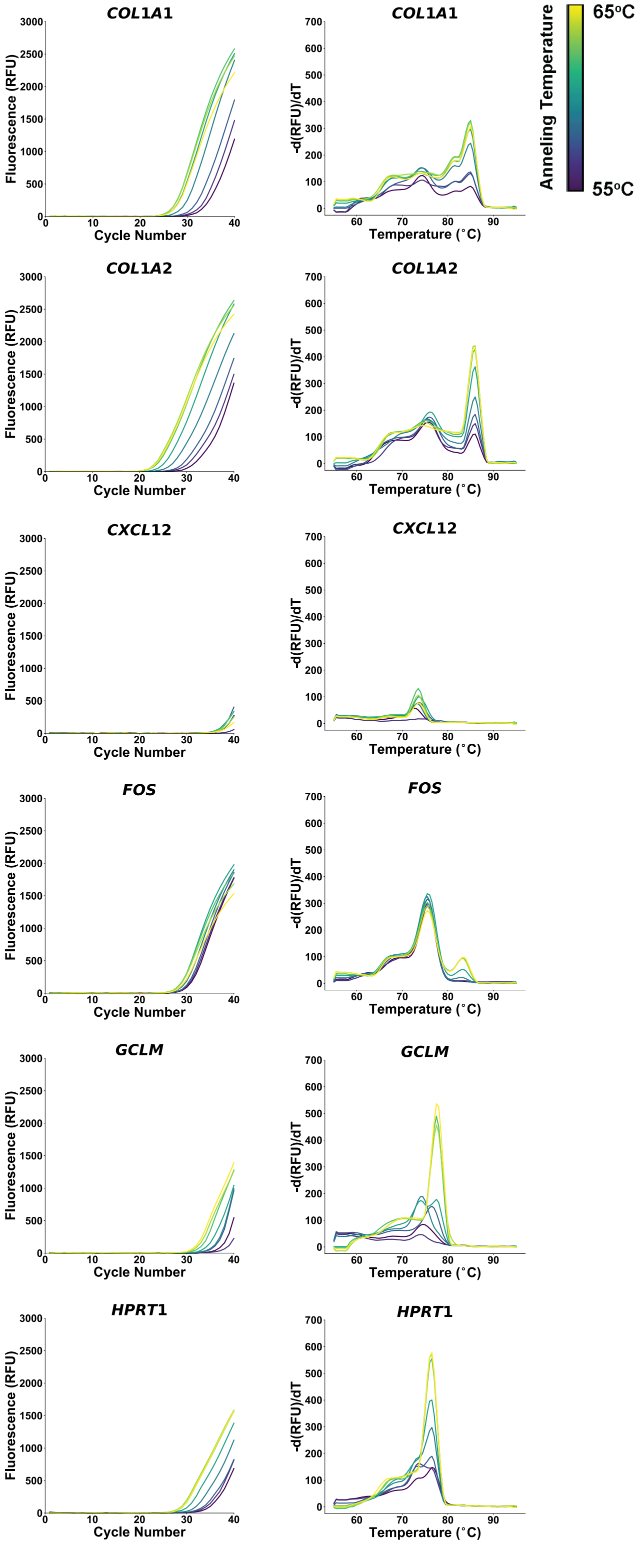


**Supplementary Figure 45. Amplification curves and melt curves for primer pairs generated using Primer Blast.** 8 different temperatures were chosen to create amplification curves (left) and melt temperature curves (right). Source data are provided as a Source Data file.

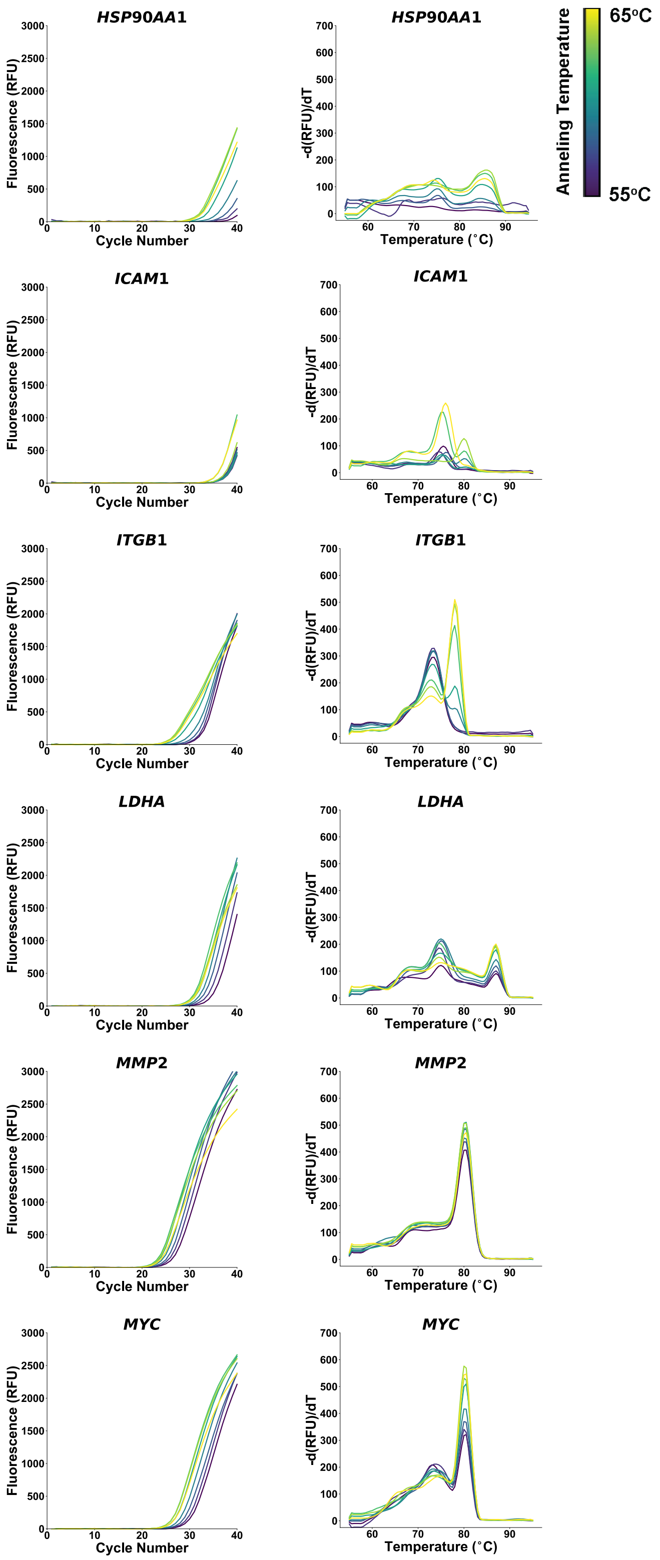


**Supplementary Figure 46. Amplification curves and melt curves for primer pairs generated using Primer Blast.** 8 different temperatures were chosen to create amplification curves (left) and melt temperature curves (right). Source data are provided as a Source Data file.

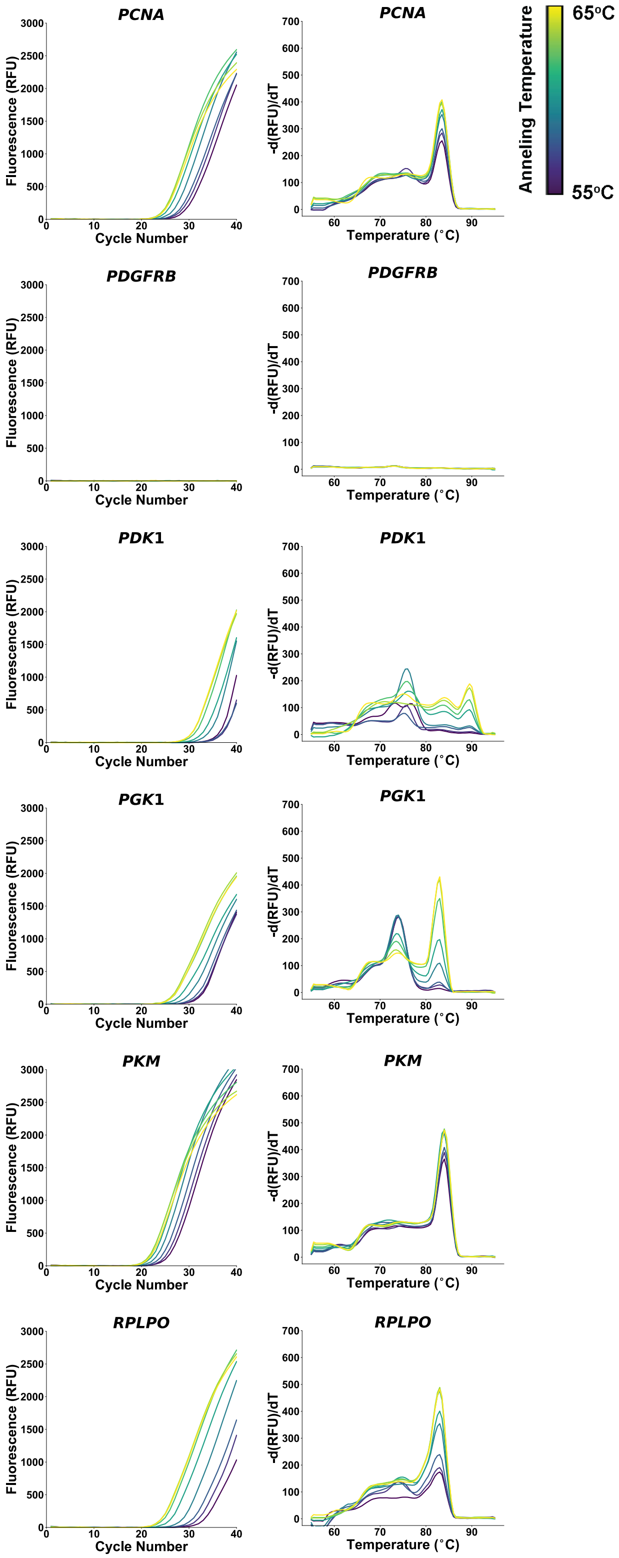


**Supplementary Figure 47. Amplification curves and melt curves for primer pairs generated using Primer Blast.** 8 different temperatures were chosen to create amplification curves (left) and melt temperature curves (right). Source data are provided as a Source Data file.

.

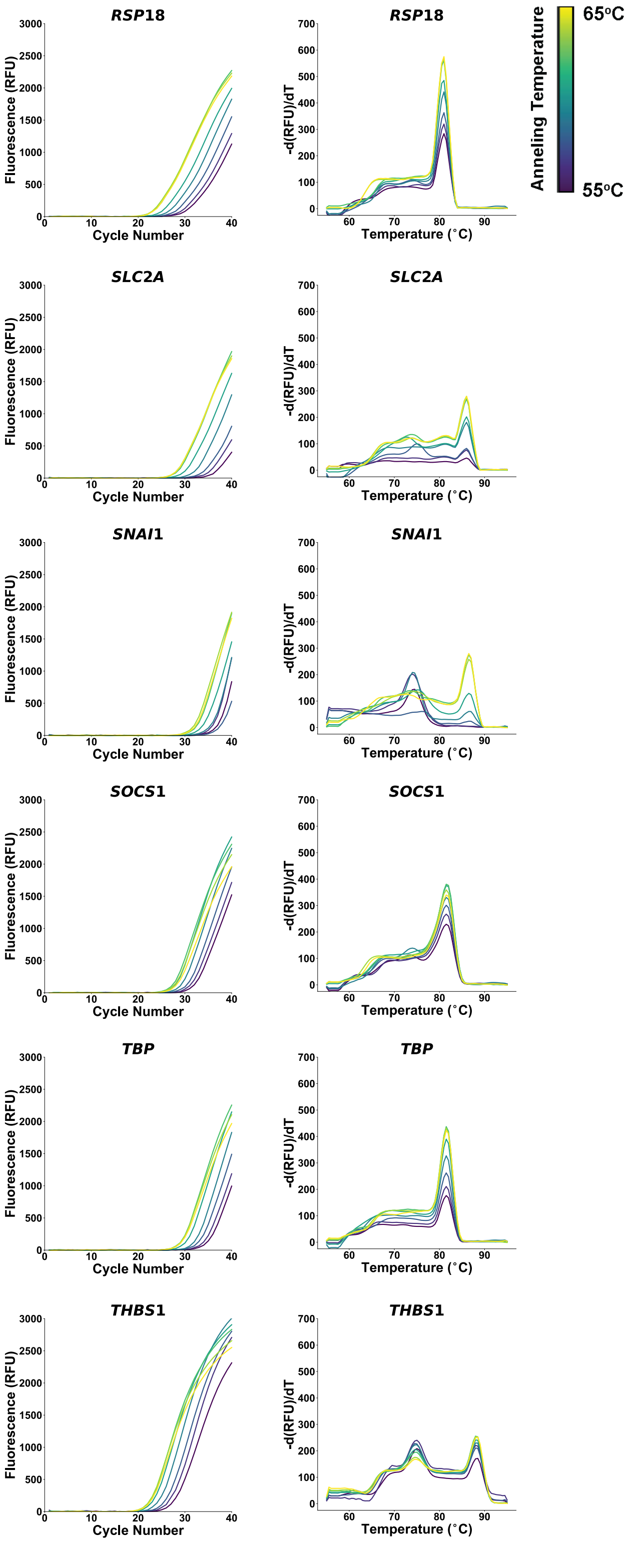


**Supplementary Figure 48. Amplification curves and melt curves for primer pairs generated using Primer Blast.** 8 different temperatures were chosen to create amplification curves (left) and melt temperature curves (right). Source data are provided as a Source Data file.

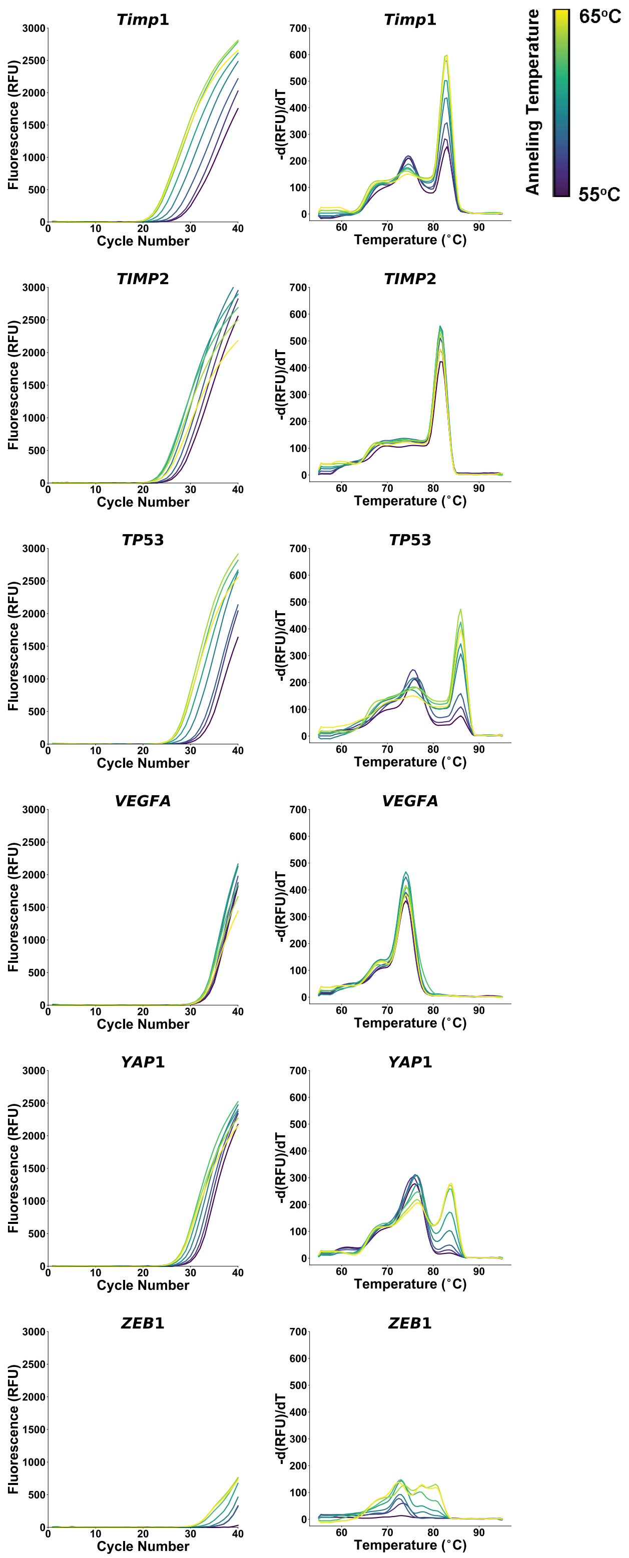


**Supplementary Figure 49. Amplification curves and melt curves for primer pairs generated using Primer Blast.** 8 different temperatures were chosen to create amplification curves (left) and melt temperature curves (right). Source data are provided as a Source Data file.

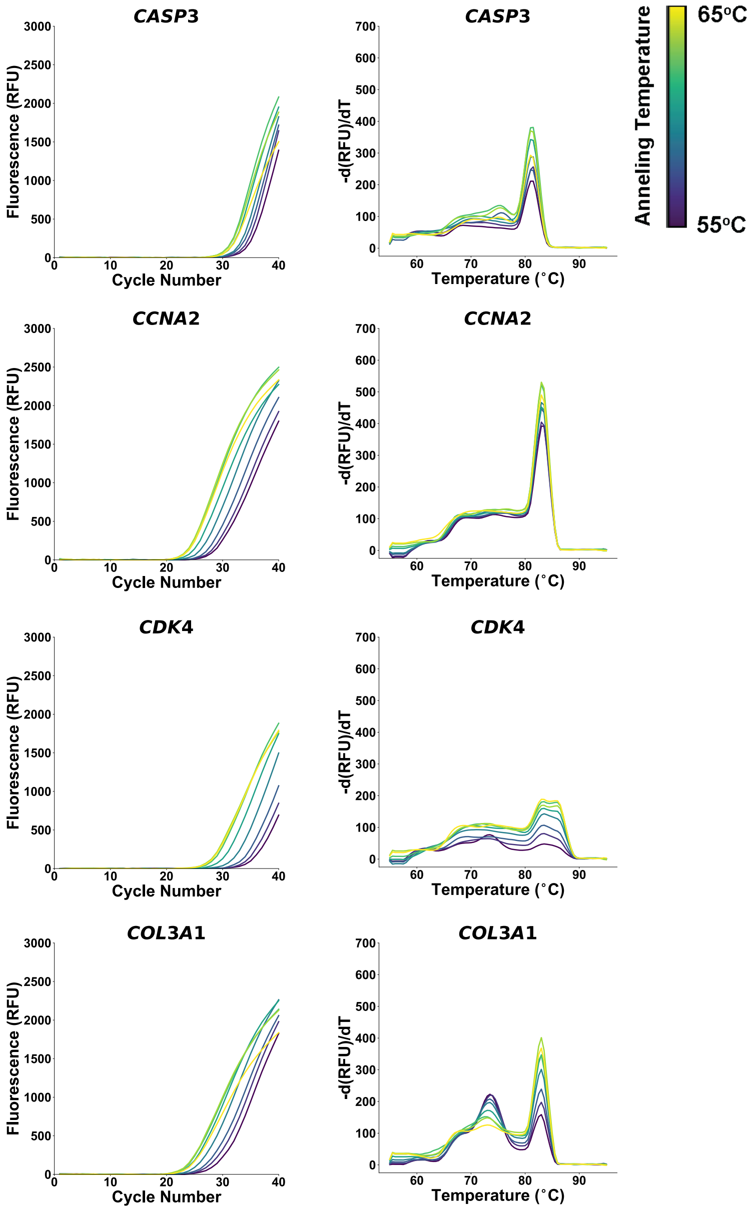


**Supplementary Figure 50. Amplification curves and melt curves for primer pairs generated using Primer Blast.** 8 different temperatures were chosen to create amplification curves (left) and melt temperature curves (right). Source data are provided as a Source Data file.

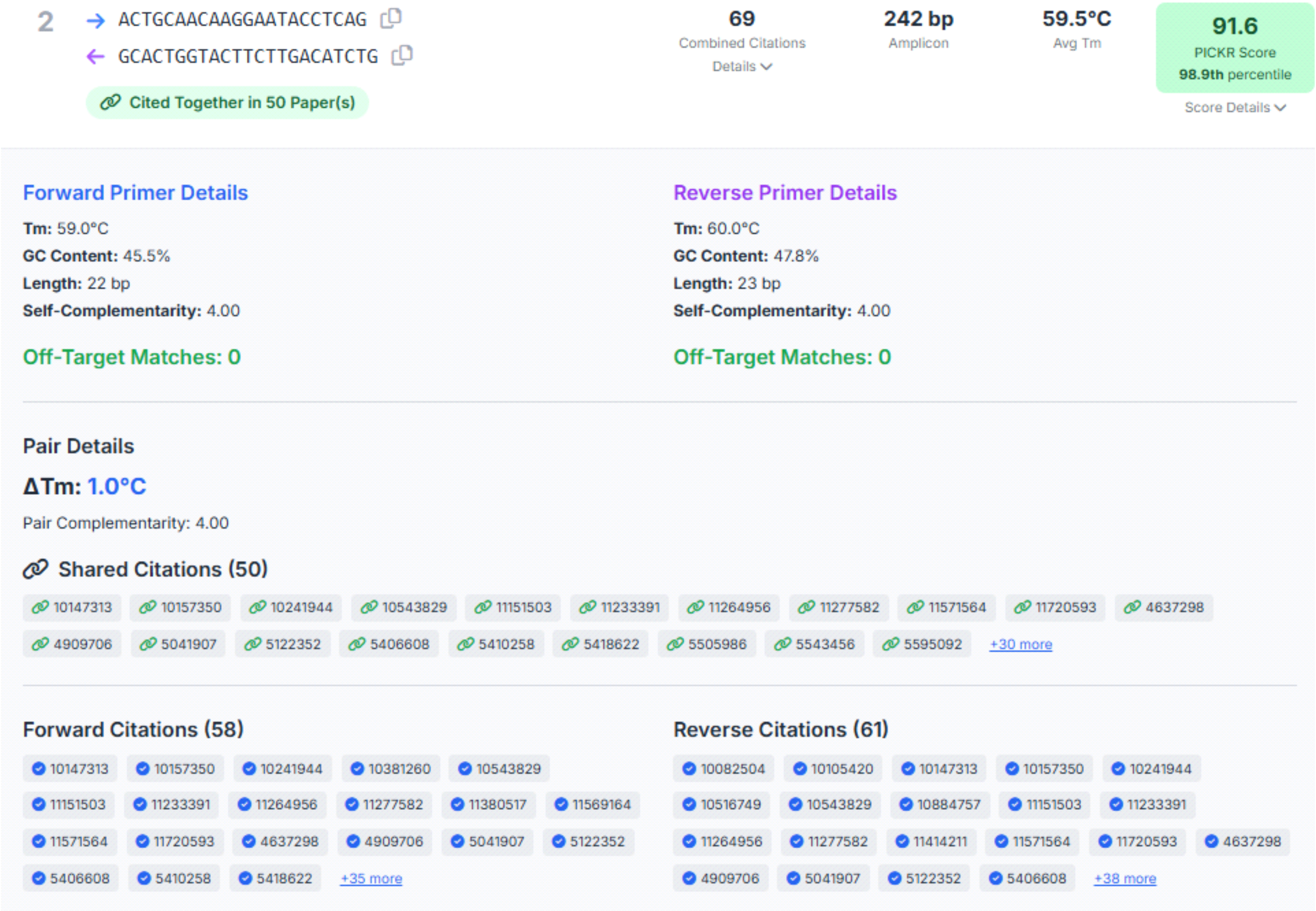


**Supplementary Figure 51. Screen shot of the primer card from www.pirmerpickr.com.** Cards display relevant data to help users consider primer details when choosing which primers to use. The code used in the creation of these data are published under a CC-BY-NC-ND license (https://creativecommons.org/licenses/by-nc-nd/4.0/deed.en).
