# Supplementary material for: Primer PICKR: literature-mined scoring platform for robust RT–qPCR primers
Source: Nat Commun. 2026 May 28;17:6938. doi: 10.1038/s41467-026-73648-2 (PMC13388996; doi:10.1038/s41467-026-73648-2)
Supplement: Supplementary file 2 — Description of Additional Supplementary Files [file 41467_2026_73648_MOESM2_ESM.pdf]

Description of Additional Supplementary Files

File Name: Supplementary Data 1

Description: **Housekeeping Gene Statistics.** List of the 15 most common genes described in the literature as 'housekeeping.' The table includes information on the number of Unique Primers, the Sum of Occurrences, and the Occurrences of Most Used Primer in the dataset. The code used in the creation of these data are published under a CC-BY-NC-ND license (https://creativecommons.org/licenses/by-nc-nd/4.0/deed.en).

File Name: Supplementary Data 2

Description: **Primer pairs for Experimental Validation.** 154 primer pairs were selected based on a distribution across PICKR score bins (90–100, 80–80, 70–79, 60–69, and 50–59) targeting 94 unique transcripts in human lung fibroblasts (IMR90) and another 24 unique transcripts in mouse NIH3T3 fibroblasts. For each primer pair, the following information is reported (left to right): Gene name, Forward primer sequence, Reverse primer sequence, Forward T_m, Reverse T_m, Theoretical_T_m, percent of GC content for the forward primer, percent of GC content for the reverse primer, number of complementary pairs, count of papers where the pair is shared, Evidence score, Biophysics score, Synergy score, and PICKR score. The code used in the creation of these data are published under a CC-BY-NC-ND license (https://creativecommons.org/licenses/by-nc-nd/4.0/deed.en).

File Name: Supplementary Data 3

Description: **Assessment of Primer Specificity.** List of 149 primers across the PICKR score range where their melt curve was analyzed for secondary peaks. For each primer, the following information is reported (left to right): Target gene name, PICKR score bin, Primer specificity (i.e., did the melt curve have a single or multiple peaks), and melt curve peak count. The code used in the creation of these data are published under a CC-BY-NC-ND license (https://creativecommons.org/licenses/by-nc-nd/4.0/deed.en).

File Name: Supplementary Data 4

Description: **Sequencing-based validation of amplicons from primer pairs exhibiting secondary melt-curve peaks.** Table contains (left to right): Best human hit (gene/locus), Query coverage (%), Percent identity (%), E-value, and Classification (on-target / off-target / inconclusive). E-values were computed by BLASTn based on Karlin–Altschul statistics, reflecting the probability of observing alignments of similar or greater score by chance given the database size. BLASTn may be accessed at https://blast.ncbi.nlm.nih.gov/ [1].

File Name: Supplementary Data 5

Description: **Primer-Blast Validation Dataset.** Table containing data on primers used for breadth and depth validation of Primer PICKR. Columns (left to right) show: Gene, Primer, Sequence (5'->3'), Template strand, Length, Start, Stop, Tm, GC%, Self complementarity, Self 3' complementarity. The code used in the creation of these data are published under a CC-BY-NC-ND license (https://creativecommons.org/licenses/by-nc-nd/4.0/deed.en).

File Name: Supplementary Data 6

Description: **Primer Database Details.** Table contains the median, average, standard deviation, and the interquartile range for (left to right): melt temperature (ºC), G/C content (%) and length (number of bases). The code used in the creation of these data are published under a CC-BY-NC-ND license (https://creativecommons.org/licenses/by-nc-nd/4.0/deed.en).

1 Altschul, S. F., Gish, W., Miller, W., Myers, E. W. & Lipman, D. J. Basic local alignment search tool. *J. Mol. Biol.* **215**, 403-410 (1990). https://doi.org:10.1016/S0022-2836(05)80360-2
