# Supplementary material for: Primer PICKR: literature-mined scoring platform for robust RT–qPCR primers
Source: Nat Commun. 2026 May 28;17:6938. doi: 10.1038/s41467-026-73648-2 (PMC13388996; doi:10.1038/s41467-026-73648-2)
Supplement: Supplementary file 10 — Transparent Peer Review file [file 41467_2026_73648_MOESM10_ESM.pdf]

nature portfolio

Peer Review File

Primer PICKR: Literature-Mined Scoring Platform for Robust RT–qPCR Primers

Corresponding Author: Professor Adam Engler

This file contains all reviewer reports in order by version, followed by all author rebuttals in order by version.

Version 0:

Reviewer comments:

Reviewer #1

(Remarks to the Author)
The manuscript introduces Primer PICKR, a large-scale primer database for RT-qPCR. Although primers are widely used, they often suffer from poor accuracy and reproducibility. To address this issue, the authors collected and automatically evaluated over seven million previously used primers, developing a tool that compiles these data into a publicly available web-based database. In addition, the authors experimentally validated a subset of these primers to further demonstrate the reliability of the proposed system.

[Major comments]
1. The criteria for primer extraction are not clearly explained. Typically, RT-qPCR primers are designed within 18-25 nt due to efficiency and specificity constraints. However, the authors queried primers within 18-40 nt. It would be helpful to clarify why the upper bound was extended to 40 nt and why shorter sequences (<18 nt) were excluded. In addition, providing a supplementary statistical summary (e.g., length, GC content, and Tm distribution) of all extracted sequences would enhance the transparency and potential utility of the dataset.

2. There is a lack of comparative benchmarking with existing primer design tools or primer databases. An analysis of PICKR scores for primers obtained from the other sources would strengthen the evidence of the score. Additionally, if possible, benchmarking not only the PICKR score but also the PCR amplification success rate across different datasets would more convincingly demonstrate the utility of the PICKR metric.

3-1. The PICKR score formula assigns the largest weight (50%) to the evidence score, which is derived from citation information. Although the authors presented correlation analyses among sub-scores (e.g., Fig. 4E), they did not directly validate the relationship between citation frequency and experimental amplification performance. The Discussion also notes that this relationship is only assumed, not empirically demonstrated. A quantitative analysis linking the evidence score to amplification outcomes would be needed to justify this weighting.
3-2. In the PICKR score calculation metric, there is a lack of explanation for how the parameters of the equation were set for (1) the weighting scheme 0.5e + 0.3b + 0.2s, and (2) the β parameters for the Biophysics score.

4. The sequence filtering step allowed only standard bases (A, C, G, T), excluding degenerate IUPAC codes. While this likely reduces false positives during parsing, it also excludes various number of valid RT-qPCR primers that contain ambiguity codes. This limitation should be acknowledged, as it may affect the completeness of the primer dataset.

[Minor comments]
1. Given the automated primer parsing approach, there is a possibility that sequences which are not true RT-qPCR primers (e.g., probes, adaptors) were collected as primer sequences. Please clarify in more detail how such false positives were identified and removed.

2. Since primer sequences were extracted from natural-language texts, did the authors encounter specific challenges? A brief discussion on how such issues were handled would be valuable for reproducibility.

3. The quality of Figure S2 is too low to read the text labels clearly.

4. The reference list is notably short for a study introducing a new database with a new scoring metric. Including more citations to prior primer design algorithms and relevant text-mining or RT-qPCR studies would strengthen the contextual grounding of the work.

[Other minor comments]
- (overall) The notation of "RT–qPCR" and "RT-qPCR" is inconsistent.
- (overall) Use italics for gene names, and Roman for protein names.
- (lines 133-137) (1) The genus name should start with a capital letter, and (2) Caenorhabditis appears in grey font.
- (line 215) "Three pair's" should be "Three pairs."
- (lines 215-216) "Three pair's (TP53_3, PDGFRb_1 and CASP9_5) on-template controls showed peaks at low annealing temperatures (Fig. S32-33)", but PDGFRb and CASP9 are not in Fig. S32–33. Possibly meant S30-33?
- (lines 204, 405, 425, 456) "Ct" is used instead of "CT" as in other lines.
- Figure 4E. "ΔTM" is used instead of "ΔTm."
- Figure S31. Are PDGFRB and PDGFRb, and PDGFRA and PDGFRa different, or are these labeling inconsistencies?

(Remarks on code availability)
1. No README file is provided, making it difficult for readers to understand the workflow.
2. The Python version used for implementation is not explicitly documented.
3. The code depends on local BLAST utility (blastn), but installation and setup instructions are missing.
4. The primer parsing regex allows ACGTRYKMSWBDHVN (all IUPAC codes), whereas the manuscript mentioned only ACGT.
5. The provided .ipynb file also does not include expected results.

Reviewer #2

(Remarks to the Author)
Review

Manuscript #: NCOMMS-25-80237-T. Nature Communications.
Title: Primer PICKR: Literature-Mined Scoring Platform for Robust RT-qPCR Primers

Summary:
This manuscript describes Primer PICKR, a novel open-access and large-scale database together with web tool that mines >400,000 publications to extract, align, and score RT-qPCR primer pairs (>7 million oligonucleotide sequences, >290,000 unique primers) for >6,000 genes across 10 model organisms. The scoring algorithm integrates literature evidence (weight 0.5), individual primer biophysics (weight 0.3), and primer-pair synergy (weight 0.2), aiming to rank primers based on community-validated performance. The authors validate 154 human primer pairs experimentally in IMR90 fibroblasts, showing high amplification success. The tool is availble at www.primerpickr.com, with monthly updates. This work addresses a key reproducibility issue in RT-qPCR, potentially saving time and resources compared to de novo design tools like Primer-BLAST or curated databases like PrimerBank.

Overall, this is a useful and timely resource that leverages literature-based large database to improve RT-qPCR assay design using primer scores. The scores integrate theoretical and practical values of RT-qPCR amplification and present the efficient ranges for primer selection. The work provides an evaluation of existing primer oligonucleotides in RT-qPCR amplification and screening the validated primers for certain genes in replication studies. The authors claim that no equivalent large-scale, continuously updated, literature-mined primer database exists. The experimental validation is valid, and the code and data are available on GitHub for accessibility and reproducibility. This work advances scientific practice and having broad implications for biomedical research, particularly in gene expression studies.
Athough this is a promissing resource, the manuscript requires major revisions before publication. Key issues include arbitrary scoring weights without empirical justification, limited validation scope (one cell type, moderate-expression genes), incomplete comparisons to existing resources, and several methodological clarifications. Addressing these will strengthen the claims and utility.

Major Points:
- Focusing on the primer mining from literature may overlook the purpose or regions of amplification. As many regions in the same gene might produce specific amplicons, newly synthesized primers might serve different purposes in research design. Therefore, there remains high percentage of newly synthesized primers that are used only once in citation. The resource should provide the regions where primers bind to amplify.
- Since the count of the articles using a certain primer was used for PICKR score calculation, higher evidence likely produces higher scores. While this score is calculated for highly cited primers, details should be added to discuss the efficiency of picked primers over the others (such as those presented in a few or only one article). Manual check and validation may be needed.
- Coverage for non-mammalian species (e.g., C. elegans or zebrafish) is notably lower than for humans. This should be highlighted as a current limitation.
- The PICKR score weights (0.5 evidence, 0.3 biophysics, 0.2 synergy) appear arbitrary. How were these determined/chosen? Please do some sensitivity analyses (e.g., varying weights) to show robustness of the method. For instance, does increasing biophysics weight improve correlation with experimental success? Eqn 1 is clear, but the caps

(e.g., 25 for shared citations) need rationale—why not scale logarithmically without caps? Additionally, the synergy score penalizes ΔTm >0°C (with a score 0 for >3°C) seems strictly, so please validate this against your data, as Fig. 4E shows weak negative correlation between ΔTm and evidence (~ -0.2-0.3?). Expand supplemental text on scoring functions.
- Validation was careful designed but limited to 94 unique transcripts in one cell line (IMR90 fibroblasts) with moderate expression, so please discuss this in details. Low-abundance transcripts or complex samples (e.g., clinical tissues, FFPE) are acknowledged as limitations, but please test a subset (e.g., 20 pairs) in another cell type or low-expression context to support the claims. Also, only SYBR Green chemistry is used – probe-based (e.g., TaqMan) compatibility should be discussed. Fig. 5G shows ~2-cycle CT improvement for high vs. low scores, but is this statistically significant across all genes? Please add p-values (e.g., ANOVA). For secondary peaks (15% overall), sequencing amplicons would confirm specificity (e.g., dimers vs. off-targets) – consider adding that for the identified 24 cases.
- The introduction pointed out some disadvantage of PrimerBank and Primer-BLAST, but there should be quantitative benchmarks. For example, how do PICKR's top pairs overlap with PrimerBank's ~130k human primers? Please test 10-20 genes where PICKR and PrimerBank differ, comparing experimental performance. Also, discuss integration: could PICKR flag isoform-specific primers (e.g., via RefSeq alignment) better than competitors? Gene coverage is strong for mammals but low for non-vertebrates (e.g., 181 for C. elegans) – please explain if this reflects literature bias or pipeline limitations.
- The resource yielded over 290K unique primers from 7.1M candidates, but ~20% non-RT-qPCR sequences (e.g., sgRNAs) were filtered heuristically. Manual validation of 100 primers ≥5 occurrences (99% accuracy) is good, but please validate a sample with <5 occurrences to assess false negatives. PDF parsing losses (e.g., paywalled supplements) are noted—quantify potential missed primers (e.g., via subsample OCR test). Cross-specificity uses Hamming distance ≤4, but justify why >4 is "highly unlikely" (cite/discuss ref. 11 appropriately). Fig. 3C shows 87% no cross-hits, but report if off-targets are pseudogenes/orthologs.
- The web interface (Fig. 6) looks intuitive, with filters and flags, but need more materials to be considered as a result. E.g., please discuss in details long-term maintenance (e.g., funding for monthly updates) and user feedback mechanisms. How does it handle user-submitted validations to refine scores? Please add metrics on current usage (e.g., queries since launch). For rare genes with low scores, suggest fallbacks (e.g., auto-link to Primer-BLAST).
- Code and database are already available for public usage. But raw validation data (e.g., melt curves, CT values) should be deposited in a repository like Zenodo or GEO, not just Table S2/S3. Ensure all scripts (e.g., for Fig. 5 analysis) are in the GitHub repo.
- The current pipeline relies exclusively on open-access Europe PMC articles. This may exclude a significant portion of high-impact literature that is paywalled or stores primer data in "locked" PDF supplements. The authors mention future iterations using OCR for locked PDFs; providing a roadmap or preliminary data on how much more "dark data" could be recovered would be valuable for the Nature Communications audience.

Minor Points:
- "Main Text Character Count" is listed as 3,520 words (limit 3,000 excluding abstract/ methods/ references/ legends), but this may be a typo (characters vs. words?).
- Trim introduction/discussion for conciseness - e.g., condense global trends description (from Line 113, Fig. 2 discussion).
- As all algorithm and verification used in this paper are for primers from open access Europe BMC articles, please clarify if primer scores retrieved in PICKR website are calculated based only on Europe BMC or other resources. (Line 99)
- Please clarify the "10th percentile" threshold used for identifying secondary peaks in melt curves. Is this a standard community threshold or one developed specifically for this study? (Line 219)
- Please admit the scraping algorithm had "insufficient accuracy" for identifying 5'–3' orientation from the text, necessitating de novo pair generation. Please briefly state how many pairs were lost or discarded due to this limitation.
- Fig-2B presents the percentage of papers using RT-qPCR in Methods, which does not indicate the uniqueness of the primers or primer sequences as mentioned in the text. (Line 66)
- Fig. 4B: hexbin could use colorbar scale.
- UMAP in Fig. 3E: please label clusters more prominently.
- Fig. 5C/D: please add arrows to peaks.
- References: please add recent reviews on RT-qPCR reproducibility (e.g., MIQE guidelines update).
- Ethics: No human/animal subjects, but confirm IRB if IMR90 RNA from donors.
- Typos/Grammar:
o Line 45, 52, 225, 356: punctuation should be after citation notes.
o Line 64: "~31% of all oligo sequences found are unique" → "31% ... are unique".
o Lines 133-134: capitalize the Genus in species' names.
o Line 144: hamming distance   Hamming distance
o Line 149-150: "prime occurrences" → "primer".
o Line 231: there is no Figure S3-33
o Line 246: "melt curve optimization" → "melting".
o Line 272-273: "31% of primers are still used only once" – consistent with Fig. 2C?
o Line 424: "thirst highest" → "third".
o Methods: Consistent units (e.g., "ug" → "µg").

(Remarks on code availability)

Reviewer #3

(Remarks to the Author)
I co-reviewed this manuscript with one of the reviewers who provided the listed reports. This is part of the Nature

Communications initiative to facilitate training in peer review and to provide appropriate recognition for Early Career Researchers who co-review manuscripts.

(Remarks on code availability)

Version 1:

Reviewer comments:

Reviewer #1

(Remarks to the Author)
The authors have addressed all concerns by providing additional analyses and experimental validation. The manuscript has been improved with several new aspects added, as reflected in the manuscript and supplementary information.

(Remarks on code availability)
The authors have provided a public GitHub repository with a well-structured README file that includes sufficient instructions for installation and execution.

Reviewer #2

(Remarks to the Author)
Review for 1st revision
Manuscript #: NCOMMS-25-80237-T. Nature Communications.
Title: Primer PICKR: Literature-Mined Scoring Platform for Robust RT-qPCR Primers

The authors have addressed most key issues in this revision, including method validation, data analysis, comparisons to existing resources, and other methodological problems. However there are still some concerns that need to be fixed before the publication:

- There should be clear discussion in the revised manuscript about the efficiency of picked primers over the others and the manual check/validation plan.
- The answear for the this question is still not clear: “Additionally, the synergy score penalizes ΔTm >0°C (with a score 0 for >3°C) seems strictly, so please validate this against your data, as Fig. 4E shows weak negative correlation between ΔTm and evidence (~ -0.2-0.3?).” Also, there should be a clearer explanation about the scoring functions. It’s not still clear why all weights are chosen and what are their impacts.
- There should be a clear discussion in revised manuscript about this: “The authors mention future iterations using OCR for locked PDFs; providing a roadmap or preliminary data on how much more "dark data" could be recovered would be valuable for the Nature Communications audience.”
- Did the author Jun K. Robbins join during this revision?
- The code URL can’t be reached: https://zenodo.org/doi/10.5281/zenodo.17058652

(Remarks on code availability)

Reviewer #3

(Remarks to the Author)
I co-reviewed this manuscript with one of the reviewers who provided the listed reports. This is part of the Nature Communications initiative to facilitate training in peer review and to provide appropriate recognition for Early Career Researchers who co-review manuscripts.

(Remarks on code availability)

Version 2:

Reviewer comments:

Reviewer #2

(Remarks to the Author)
The authors have addressed all issues in this revision. The manuscript has been improved with added materials and discussions, it's clearer now. The web tool has also been updated to be a better version. I'm happy with current form of the manuscript (although it should be revised to fix some typos).

(Remarks on code availability)
The code URL now can be reached and I have found all code developed for the work. I did not have a chance to run the

whole code but it looks okay based on the results from code in the Python notebook file.

Reviewer #3

(Remarks to the Author)
I co-reviewed this manuscript with one of the reviewers who provided the listed reports. This is part of the Nature Communications initiative to facilitate training in peer review and to provide appropriate recognition for Early Career Researchers who co-review manuscripts.

(Remarks on code availability)

Open Access This Peer Review File is licensed under a Creative Commons Attribution 4.0 International License, which permits use, sharing, adaptation, distribution and reproduction in any medium or format, as long as you give appropriate credit to the original author(s) and the source, provide a link to the Creative Commons license, and indicate if changes were made.
In cases where reviewers are anonymous, credit should be given to 'Anonymous Referee' and the source.
The images or other third party material in this Peer Review File are included in the article’s Creative Commons license, unless indicated otherwise in a credit line to the material. If material is not included in the article’s Creative Commons license and your intended use is not permitted by statutory regulation or exceeds the permitted use, you will need to obtain permission directly from the copyright holder.
To view a copy of this license, visit https://creativecommons.org/licenses/by/4.0/

We would like to thank the editors and reviewers at Nature Communications for careful consideration of the manuscript and for their comments. We have responded below to each of the concerns (in **BLACK**) on a point-by-point basis in **BLUE** and made text changes to the manuscript in **RED**.

Reviewer #1

The manuscript introduces Primer PICKR, a large-scale primer database for RT-qPCR. Although primers are widely used, they often suffer from poor accuracy and reproducibility. To address this issue, the authors collected and automatically evaluated over seven million previously used primers, developing a tool that compiles these data into a publicly available web-based database. In addition, the authors experimentally validated a subset of these primers to further demonstrate the reliability of the proposed system.

[Major comments]

1. The criteria for primer extraction are not clearly explained. Typically, RT-qPCR primers are designed within 18-25 nt due to efficiency and specificity constraints. However, the authors queried primers within 18-40 nt. It would be helpful to clarify why the upper bound was extended to 40 nt and why shorter sequences (<18 nt) were excluded. In addition, providing a supplementary statistical summary (e.g., length, GC content, and Tm distribution) of all extracted sequences would enhance the transparency and potential utility of the dataset.

We thank the reviewer for this comment as it is an important point to clarify. At 16 base pairs, the likelihood of an identical sequencing being found in the human genome is around 50%. Setting our minimal threshold to 18 drops this to 2% and follows more conventional guidelines. The upper bound of 40 base pairs was due to some primers in the 1990s and early 2000s reaching into the 30s, as seen in Figure 2D. However, users are able to filter this length to 18-25 or 18-30 if they choose using the PRICKR website. That being said, we agree that a summary table is a good idea to address this point, hence the following table has been added to the manuscript at Supplemental Table S5:

	Tm	GC_pct	Length
Median	63.3	52	20
Average	60.4	51.8	21.1
STDev	3.1	6.9	2.3
25-75% IQR	60.3 - 62.5	52 - 55	20 - 22

2. There is a lack of comparative benchmarking with existing primer design tools or primer databases. An analysis of PICKR scores for primers obtained from the other sources would strengthen the evidence of the score. Additionally, if possible, benchmarking not only the PICKR score but also the PCR amplification success rate across different datasets would more convincingly demonstrate the utility of the PICKR metric.

The new Figure 6C provides benchmarking of amplification for Primer-BLAST-created primers for a large set of human primers. So long as the PICKR score exceeds 80, the amplification for PICKR primers is better.

3-1. The PICKR score formula assigns the largest weight (50%) to the evidence score, which is derived from citation information. Although the authors presented correlation analyses among

sub-scores (e.g., Fig. 4E), they did not directly validate the relationship between citation frequency and experimental amplification performance. The Discussion also notes that this relationship is only assumed, not empirically demonstrated. A quantitative analysis linking the evidence score to amplification outcomes would be needed to justify this weighting.

We thank the reviewer for this inciteful comment. The referee raises an important point, as we have done more analyses to justify our evidence score weights. As figure on the right shows, for the 11 primers that failed in our data set, low evidence score (Median on 59) strongly predicted failure, with minimal effect from biophysics (Median of 89) or synergy (Median of 84). That figure has been added as a new Figure 5B. The following text has been added to the manuscript:

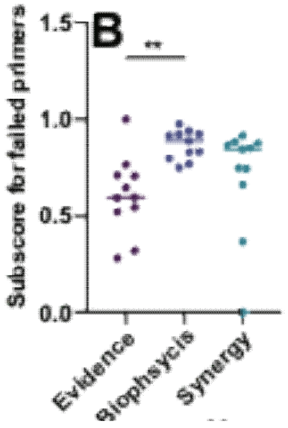

"Of the 11 primer pairs that failed, the median evidence score was 59, with biophysics and synergy median scores of 89 and 84 respectively. Together, this supports our algorithm weighting with evidence score accounting for half the PICKR score."

For the 22 primers tested with secondary peaks, a smaller difference was seen between all three sub-scores, though synergy had a long tail towards poor scores. We felt that the lower sub-score variance was less informative if one is attempting to identify secondary peaks, hence we did not include the figure on the right in the manuscript ("double peak primers").

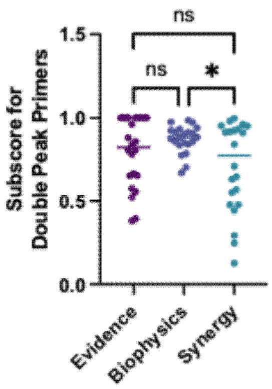

3-2. In the PICKR score calculation metric, there is a lack of explanation for how the parameters of the equation were set for (1) the weighting scheme 0.5e + 0.3b + 0.2s, and (2) the β parameters for the Biophysics score.

We agree with the referee, and in addition to new data and explanation from comment 3-1, we have added the following text to the second paragraph of the discussion section:

"Low evidence score also predicted primer pair failure more than any other metric, supporting a 0.5 weighting; although, a flawed primer may propagate as noted by a minority of primers tested with double peaks in their melt curves. Biophysics and synergy scores were less predictive of primer quality when no off-target effects are found, yet off targets will lead to significant trust issues with primer choice, reflecting the need for a higher weight for biophysics than synergy. The penalty scores for the types off-target hits reflect the perceived increase in mistrust of primer pairs."

4. The sequence filtering step allowed only standard bases (A, C, G, T), excluding degenerate IUPAC codes. While this likely reduces false positives during parsing, it also excludes various

number of valid RT-qPCR primers that contain ambiguity codes. This limitation should be acknowledged, as it may affect the completeness of the primer dataset.

> We thank the referee for raising this important discussion point and have added the following text to the discussion:
>
> > "Furthermore, to reduce the instances of false positives, sequences only containing standard bases (A, C, T, and G) we used. This led to a loss of valid primer pairs which limits our database's ability to suggest primers which identify gene families or homologs where exact sequences vary."

[Minor comments]
1. Given the automated primer parsing approach, there is a possibility that sequences which are not true RT-qPCR primers (e.g., probes, adaptors) were collected as primer sequences. Please clarify in more detail how such false positives were identified and removed.

> The following text was modified in the manuscript to highlight our removal of false positives:
>
> > "Using sequences with multiple occurrences greatly reduced our false positive rate (**Fig. 1C**). Therefore, we aggregated oligo sequences with ≥3 occurrences, leading to 290,191 unique RT-qPCR primers were identified (53% of the 7,170,000 oligos)… To reduce a false positive rate to near 0, primers had to match exactly one transcript with 0 mismatched base pairs for each species to be added to species specific gene lists."

2. Since primer sequences were extracted from natural-language texts, did the authors encounter specific challenges? A brief discussion on how such issues were handled would be valuable for reproducibility.

> The following text was added:
>
> > "Due to the enormous number of ways in which scientists list primers in their manuscripts, meta-data extraction for primers was not possible, including primer direction, melt temperatures used, and even which genes primers were linked to. This led to our algorithm needing to rederive all of this information using BLAST and other algorithms"

3. The quality of Figure S2 is too low to read the text labels clearly.

> We apologize for the quality of the labels. We believe that this has been fixed in the current draft.

4. The reference list is notably short for a study introducing a new database with a new scoring metric. Including more citations to prior primer design algorithms and relevant text-mining or RT-qPCR studies would strengthen the contextual grounding of the work.

> We have added 5 references to the introduction to better relate the manuscript to prior algorithms.

[Other minor comments]

- (overall) The notation of "RT–qPCR" and "RT-qPCR" is inconsistent.

We have fixed the inconsistent notation.

- (overall) Use italics for gene names, and Roman for protein names.

We have fixed the use of italics and apologize for the inconsistency.

- (lines 133-137) (1) The genus name should start with a capital letter, and (2) Caenorhabditis appears in grey font.

We have fixed the inconsistent notation and color.

- (line 215) "Three pair's" should be "Three pairs."

We have fixed the grammar error.

- (lines 215-216) "Three pair's (TP53_3, PDGFRb_1 and CASP9_5) on-template controls showed peaks at low annealing temperatures (Fig. S32-33)", but PDGFRb and CASP9 are not in Fig. S32–33. Possibly meant S30-33?

We have fixed the notation.

- (lines 204, 405, 425, 456) "Ct" is used instead of "CT" as in other lines.

Ct notation has been updated to CT throughout the manuscrip.

- Figure 4E. "ΔTM" is used instead of "ΔTm."

Figure 4E has been updated with the correct notation.

- Figure S31. Are PDGFRB and PDGFRb, and PDGFRA and PDGFRa different, or are these labeling inconsistencies?

We have fixed the inconsistent notation.

Remarks on code availability
1. No README file is provided, making it difficult for readers to understand the workflow.

The README file is available at https://github.com/tmolley2/PICKR.

2. The Python version used for implementation is not explicitly documented.

The README file specifies the version recommended for uses, which is at least version 3.9 or newer (the current version is 3.14.2).

3. The code depends on local BLAST utility (blastn), but installation and setup instructions are missing.

The README file now includes installation and setup instructions for blastn.

4. The primer parsing regex allows ACGTRYKMSWBDHVN (all IUPAC codes), whereas the manuscript mentioned only ACGT.

This has been removed.

5. The provided .ipynb file also does not include expected results.

We have included a new section in the README file about expected results from the code.

Reviewer #2 (Remarks to the Author):

Summary:
Overall, this is a useful and timely resource that leverages literature-based large database to improve RT-qPCR assay design using primer scores….Although this is a promising resource, the manuscript requires major revisions before publication. Key issues include arbitrary scoring weights without empirical justification, limited validation scope (one cell type, moderate-expression genes), incomplete comparisons to existing resources, and several methodological clarifications. Addressing these will strengthen the claims and utility.

We thank the reviewer for these positive comments and criticisms that we address below. We believe that these changes have markedly improved the paper, and we hope that the reviewer agrees.

Major Points:
- Focusing on the primer mining from literature may overlook the purpose or regions of amplification. As many regions in the same gene might produce specific amplicons, newly synthesized primers might serve different purposes in research design. Therefore, there remains high percentage of newly synthesized primers that are used only once in citation. The resource should provide the regions where primers bind to amplify.

The referee raises an important point as we have now added the primer position to the primer card on the database website for each primer and pair.

- Since the count of the articles using a certain primer was used for PICKR score calculation, higher evidence likely produces higher scores. While this score is calculated for highly cited primers, details should be added to discuss the efficiency of picked primers over the others (such as those presented in a few or only one article). Manual check and validation may be needed.

We thank the reviewer for this concern. The nature of crowd-sourcing any data is that it will be at the mercy of the community. By definition, Primer PICKR will miss both good and bad primers that were not previously used frequently in the literature. However, the sub-scores not dependent on frequency, i.e., biophysics and synergy scores, should balance this out somewhat. To put it another way, if a terrible primer–despite being terrible–happen to be used often, half of the PICKR score would still disfavor it so that it would not be a top hit. As an example of this

in the PICKR database, HPRT1, which is one of the genes used in Figure 5H, has a top cited primer with a score of 85 with 540 combined citations. However, the primer with the best score of 96.7 only has 246 combined citations. So while citations can drive a PICKR score up, it is unlikely to have a score close to 100. Indeed the figure at right illustrates that for HPRT1, the highest scores have moderate citations and the correlation is weak; good primer scores can be moderately cited.

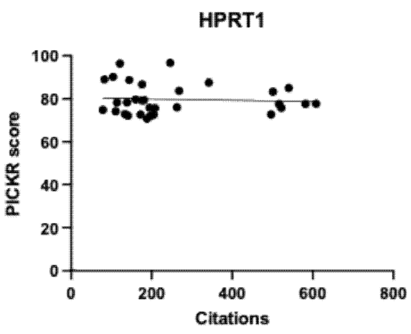

Assuming that this database becomes widely adopted, we expect that the field will converge on good primers that have had reasonable use in the past and that over time, the database will help the field converge on the best primers, i.e., the relationship above will become tighter and with a more positive slope. Does that mean that poorly cited but good primers might still be missed? Yes, but there will still be a significant number of good and highly cited primers as well as the field converges after using this tool.

- Coverage for non-mammalian species (e.g., C. elegans or zebrafish) is notably lower than for humans. This should be highlighted as a current limitation.

We agree with the referee and have added the following to the manuscript's discussion section (3rd paragraph):

"Our database also has limited representation of genes for less studied species which share less genetic overlap with humans, such as C. elegans or Zebrafish, due to the inherent nature of crowd sourcing."

- The PICKR score weights (0.5 evidence, 0.3 biophysics, 0.2 synergy) appear arbitrary. How were these determined/chosen? Please do some sensitivity analyses (e.g., varying weights) to show robustness of the method. For instance, does increasing biophysics weight improve correlation with experimental success? Eqn 1 is clear, but the caps (e.g., 25 for shared citations) need rationale—why not scale logarithmically without caps? Additionally, the synergy score penalizes ΔTm >0°C (with a score 0 for >3°C) seems strictly, so please validate this against your data, as Fig. 4E shows weak negative correlation between ΔTm and evidence (~ -0.2-0.3?). Expand supplemental text on scoring functions.

We thank the referee for raising this important consideration. We analyzed our failed primer sequences for a similar concern that reviewer 1 had which highlighted the strong link between primer failure and poor evidence score. The biophysics weight is not well correlated when there are no off-target effects found, but we chose the 0.3 weight to enable off-targets to significantly reduce primer scores when they are found. While a sub-score of 0 for a $\Delta T_m > 0$ seems harsh, this actually only reflects a maximum decrease in final picker score of 3%, allowing for us to slightly shift primers with more favorable T_m's higher up the list without significant penalty to deviations.

To the reviewer's question about a log scale vs. a cap, having a cap to limit the score prevents the equation from getting skewed by the limited number of very highly used yet diverse primer sets, e.g., loading control genes with hundreds of primers with a few that are highly used. Without a cap, primers with 1000+ citations would overwhelm/dominate the score. The figure at right illustrates the distribution of primer citations for both forward and reverse primer citations. Capping at 25 vs. 50 citations means that 6.7% vs. 2.6% of the data is compressed, respectively. The cap at 25 allows for more differentiation between citations at the lower end of the distribution. To put it another way, the incremental improvement in primer quality above 25 citations is likely minimal.

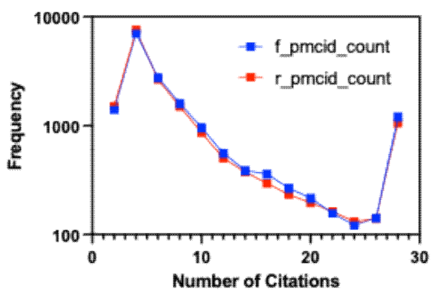

- Validation was careful designed but limited to 94 unique transcripts in one cell line (IMR90 fibroblasts) with moderate expression, so please discuss this in details. Low-abundance transcripts or complex samples (e.g., clinical tissues, FFPE) are acknowledged as limitations, but please test a subset (e.g., 20 pairs) in another cell type or low-expression context to support the claims.

We thank the reviewer for this concern. While we discuss low abundance genes with additional melt temperature optimization in the third paragraph of the Bench-top Validation section of the results, we have also addressed the reviewer's other request for validation in a non-human line; we picked the mouse NIH3T3 fibroblast line, which is well-documented in the literature, in which to test primers from PICKR. We found that of the 24 primers tested from PICKR, 95% amplified with 12% having double peaks. Hence there was an 83% success rate; these data are shown in a new Fig. 5J. The following text was added to the main text:

"Further, to access cross-species performance, we tested 24 mouse primer pairs (**Table S2,** PICKR score >80) using NIH 3T3 fibroblast cells. Of these, 95% amplified with 12% having double peaks (83% success rate; **Fig. 5J, S37-40**), similar to amplification of human PICKR scores >80 (**Fig. 5A**), supporting the generalizability of the PICKR scoring across species."

Method section was updated as well:

"*Cell culture.* IMR90 human lung fibroblasts (ATCC CCL-186) and NIH/3T3 (ATCC CRL-1658) were cultured in DMEM (Thermo Fisher Scientific 11965092) with 10% fetal bovine serum (Omega Scientific FB-01) and 1% penicillin streptomycin (Thermo Fisher Scientific 15140122)."

Also, only SYBR Green chemistry is used – probe-based (e.g., TaqMan) compatibility should be discussed.

TaqMan primers use a specified quencher probe that is predesigned for primer sequences and sold that way. Our database is incompatible with TaqMan probes as probe chemistry can significantly change biophysical properties of the probe

(making that part of our score less predictive). However, we have added the following discussion to the manuscript to reflect this:

> "In addition, our database is limited to SYBR Green qPCR, as the requirement for proprietary, probe-dependent design in TaqMan assays precludes large-scale, cross-study primer reuse and ranking. "

Fig. 5G shows ~2-cycle CT improvement for high vs. low scores, but is this statistically significant across all genes? Please add p-values (e.g., ANOVA).

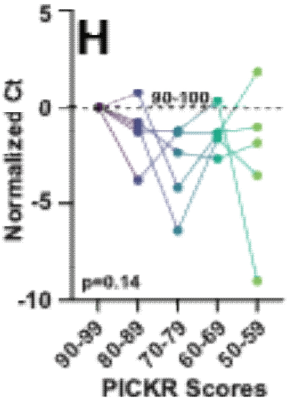

We thank the reviewer for raising this important question. The data for the five genes, i.e., *FN*, *GAPDH, HPRT1, TBP,* and *VIM*, shows increased variance when using primers with lower PICKR scores for the same gene relative to primers with higher PICKR scores. We illustrate that better now in Figure 5H by tracking each gene across the different score bins; standard deviation increases from 1.65 (80-89 bin) to 4.02 (50-59 bin). More to the reviewer's point, we now note the ANOVA result for bin score, which is p=0.14 for these 5 genes. While not quite 0.05, the increased variance in Ct reduces our confidence in the reproducibility and reliability of primers with lower PICKR scores.

For secondary peaks (15% overall), sequencing amplicons would confirm specificity (e.g., dimers vs. off-targets) – consider adding that for the identified 24 cases.

We thank the reviewer for this comment. We sequenced PCR products from 12 out of the 24 reactions exhibiting visually significant distinct secondary melt-curve peaks (n = 12 amplicons) and aligned the resulting sequences against the human reference genome using BLASTn. In all cases, the dominant amplicon mapped uniquely to the intended target gene with high confidence (mean query coverage = 97%, mean percent identity = 99.6%, E-values ranging from 10^{-36} to 10^{-139}). No dominant off-target genomic loci were detected, indicating that secondary peaks likely reflect low-abundance artifacts (e.g., primer dimers or minor mis-primed species) rather than off-target amplification.

However, one primer set, i.e., TP53, produced no detectable amplification in sample wells and yielded product only in the no-template control, consistent with primer-dimer formation; no sequenceable amplicon was obtained and this case was excluded from specificity classification. These results are summarized in Supplementary Table S4. We have added the following text to the manuscript to address the point:

> "From the 24 primer pairs exhibiting secondary melt peaks, a subset of 12 reactions with visually distinct, high-amplitude secondary peaks were selected for amplicon purification and Sanger sequencing, and resulting reads were aligned to the Homo sapiens reference genome using BLASTn to assess genomic specificity (**Fig. S34, Table S4**). In all cases, the dominant amplicon mapped uniquely to the intended target gene with high

confidence (mean query coverage = 97%, mean percent identity = 99.6%, E-values ranging from 10^{-36} to 10^{-139}). No dominant off-target genomic loci were detected, indicating that secondary peaks likely reflect low-abundance artifacts (e.g., primer dimers or minor mis-primed species) rather than off-target amplification. However, one primer set, i.e., *TP53_3*, had no sequenceable amplicon and this case was excluded from specificity classification."

The methods section was updated with the following text addition:

''For reactions exhibiting secondary melt peaks and passing CT thresholds, PCR products were purified and subjected to Sanger sequencing. Sequence reads were aligned to the Homo sapiens reference genome (taxid:9606) using BLASTn (core_nt), and classified as on-target when the top hit exhibited ≥90% query coverage, ≥90% percent identity, and an E-value $\leq 1 \times 10^{-10}$."

- The introduction pointed out some disadvantage of PrimerBank and Primer-BLAST, but there should be quantitative benchmarks. For example, how do PICKR's top pairs overlap with PrimerBank's ~130k human primers? Please test 10-20 genes where PICKR and PrimerBank differ, comparing experimental performance.

We thank the reviewer for this comment. We have added both additional breadth and depth to the individually curated samples in Figures 6. For depth, we tested amplification of 10 human primers each from *GAPDH*, *VIM*, and *FN1* in a new Fig. 6A-C. 97% amplified without double peaks though CT values were less consistent, even from the same template. For breadth, when using Primer-BLAST to design primers for 46 additional human genes (taking the first hit), we found an 89% amplification rate but 35% double peak rate (54% success rate; Fig. 6D); this was worse than high PICKR scored primers, which when the score is >80 has a >90% chance of amplifying with almost no double peaks (Fig. 5A).

As mentioned in initial comments for reviewer 1, we believe that Primer PICKR's crowd-sourcing advantage will improve with time and use as a handful of primer sets per gene are preferentially cited over others (there is a positive feedback in the literature score). We added the following to the main text:

"To benchmark PICKR against existing tools, we compared its performance with Primer-BLAST as it is one of the most widely used primer design tools (~7000 citations)[9]. We tested both the **depth** (per-gene quality) and **breadth** (cross-gene reliability) of Primer-BLAST. For depth, we tested the amplification of 10 human primer pairs each for *GAPDH*, *VIM*, and *FN1* generated using Primer-BLAST (**Table S5**). Overall, we found that almost all primer pairs amplified (**Fig. 6A; S41-43**), though on average ~20% of the amplicons had double peaks (**Fig. 6B**) and the CT values were less consistent (**Fig. 6C**), even from the same template. Although we broadly categorized them as double peaks, some of the amplifying primers have additional features such as delayed amplification or abnormal curve shapes, which suggested suboptimal design despite successful amplification (**Fig. 6B**). For breadth benchmarking, we randomly selected 46 human primer pairs generated by Primer-BLAST for

genes previously tested in the 90–100 PICKR score group (Fig. 5A, **Table S5**). We observed ~89% amplification rate but 35% double peak rate (i.e., a 54% success rate; **Fig.6D, S44-51**) making outcomes likely worse than PICKR, especially for human PICKR scores >80. Given these data, we believe that the PICKR approach can save significant cost and help the community build consensus for high performance primers."

Also, discuss integration: could PICKR flag isoform-specific primers (e.g., via RefSeq alignment) better than competitors?

We thank the reviewer for bringing this point up. It has been our experience that in searching for terms such as isoform-specific primers, we do not find them to be articulated prominently in the literature. Since we are at the mercy of what the literature offers, we hope that if noting isoform specificity becomes common in the future, that feature could be added. However for now since Primer PICKR uses empirical data rather than a model like PrimerBank, our output is validated, even if it is not specifically mapped to a specific isoform.

Gene coverage is strong for mammals but low for non-vertebrates (e.g., 181 for C. elegans) – please explain if this reflects literature bias or pipeline limitations.

The is due to literature bias as well as limited genetic overlap with the human genome. We have added discussion to the manuscript that was highlighted above:

"Our database also has limited representation of genes for less studied species which share less genetic overlap with humans, such as C. elegans or Zebrafish, due to the inherent nature of crowd sourcing."

- The resource yielded over 290K unique primers from 7.1M candidates, but ~20% non-RT-qPCR sequences (e.g., sgRNAs) were filtered heuristically. Manual validation of 100 primers ≥5 occurrences (99% accuracy) is good, but please validate a sample with <5 occurrences to assess false negatives.

The final data set used the 290k primers with at least 3 occurrences. We had 2.5 million unique oligos with occurrences of only 1-2 times with a false positive rate of ~21%. This led to our selection criteria giving ~2.3 million unique RT-qPCR primers, of which we used only 290k (87% on-target rate). While this rate is quite high, given that our database's goal is to curate sequences which are commonly used, we find this value sufficient.

PDF parsing losses (e.g., paywalled supplements) are noted—quantify potential missed primers (e.g., via subsample OCR test).

We have added the following text to the manuscript to address the point:

"Given an average of 18 nucleotides per paper and ~220,000 papers mapped, we may have missed ~3-4 million unique oligos, equating to 30% of our database."

Cross-specificity uses Hamming distance ≤4, but justify why >4 is "highly unlikely" (cite/discuss ref. 11 appropriately).

We have added the following text to the manuscript to address this:

> "At ≥5 mismatches, predicted duplex stability drops by orders of magnitude (ΔΔG ≈ +5–10 kcal/mol → ~10^3–10^5-fold weaker binding at qPCR temperatures), and the likelihood of a 3′-proximal mismatch becomes high, further suppressing extension."

Fig. 3C shows 87% no cross-hits, but report if off-targets are pseudogenes/orthologs.

Given low Hamming distances for the off-targets (99% are <4 bases), the mismatches do not likely involve pseudogenes. Blasting these sequences, we do not find them mapping onto orthologs, despite low species coverage in other model organisms.

- The web interface (Fig. 6) looks intuitive, with filters and flags, but need more materials to be considered as a result. E.g., please discuss in details long-term maintenance (e.g., funding for monthly updates) and user feedback mechanisms. How does it handle user-submitted validations to refine scores? Please add metrics on current usage (e.g., queries since launch).

We thank the referee for this suggestion and have added the following text to the manuscript:

> "The database is hosted on PythonAnywhere for $6 a month with multiple workers, allowing for simple long-term hosting at a low price. This enables the database to handle millions of queries per day. Current maximum usage was 10,000 queries in 12 hours with minimal impact on loading times, with 100,000 queries since launch (mostly web crawlers)."

Currently, the user feedback is only accepted via email. However, the scoring architecture is explicitly modular, allowing us to modify the database in the future to incorporate user-submitted experimental validations to be to the evidence subscore rather. In practice, such validations would be weighted by metadata completeness and reproducibility and used to update scores conservatively, preserving robustness while enabling iterative refinement.

For rare genes with low scores, suggest fallbacks (e.g., auto-link to Primer-BLAST).

This functionality has been included.

- Code and database are already available for public usage. But raw validation data (e.g., melt curves, CT values) should be deposited in a repository like Zenodo or GEO, not just Table S2/S3. Ensure all scripts (e.g., for Fig. 5 analysis) are in the GitHub repo.

Figures, tables and, other raw data for validation is available at https://doi.org/10.6084/m9.figshare.31082878. Note that we include validation for Figure 5J and 6B-C as well as their associated raw amplification and melt curves in Figure S36-50.

- The current pipeline relies exclusively on open-access Europe PMC articles. This may exclude a significant portion of high-impact literature that is paywalled or stores primer data in "locked" PDF supplements. The authors mention future iterations using OCR for locked PDFs; providing a roadmap or preliminary data on how much more "dark data" could be recovered would be valuable for the Nature Communications audience.

> Please see the response to the above comment about quantifying the missed primers. Overall, we expect that this number will go down over time since the NIH mandates for open publication after 12 month/no paywall, European commission requires open access for all papers published after 2020, and the UC system, MIT, and Harvard expressed preference for open access. As mentioned below, we estimate missing ~3-4M oligos but did capture >8.1M oligos total. We believe that this is a good start to illustrate general principles of RT-qPCR primers and their curation into a single crowd-sourced database.

Minor Points:
- "Main Text Character Count" is listed as 3,520 words (limit 3,000 excluding abstract/ methods/ references/ legends), but this may be a typo (characters vs. words?).

> This is not a typo. Due to the changes mentioned below with reductions to the introduction but expansion of the discussion, the word count has grown slightly to 4,220 words. We recognize that the word count is higher than the limit, but due to clarifications to reviewers, we were less able to reduce the word count than we hoped. Given the online nature of the journal and the importance of the additions to address reviewer comments, we trust that some degree of editorial flexibility is possible.

- Trim introduction/discussion for conciseness - e.g., condense global trends description (from Line 113, Fig. 2 discussion).

> We cut 121 words from the introduction (basically one paragraph of text). However, due to clarifications in response to reviewer inquiries, 268 words were added to discussion.

- As all algorithm and verification used in this paper are for primers from open access Europe BMC articles, please clarify if primer scores retrieved in PICKR website are calculated based only on Europe BMC or other resources. (Line 99)

> Using a back-of-the-envelope calculation assuming an average of 18 nucleotides per paper and ~220,000 papers mapped, we estimate that there could be another ~30% of our database that was not captured. This limitation is discussed in the first paragraph of the results section:
>
> > "Given an average of 18 nucleotides per paper and ~220,000 papers mapped, we may have missed ~3-4 million unique oligos, equating to 30% of our database. Future text-mining iterations will incorporate optical character recognition for locked PDFs to further close this gap. However, the pipeline yielded 8.1 million candidate oligos."

- Please clarify the "10th percentile" threshold used for identifying secondary peaks in melt curves. Is this a standard community threshold or one developed specifically for this study? (Line 219)

We apologize for the confusion as this is a tolerance that we set to ensure detection of secondary peaks across the entire primer dataset. The threshold itself was determined iteratively to ensure that only true secondary peaks were counted. We have updated the language regarding this “10th percentile” threshold to be more clear:

> While the melting temperature of *PDGFRb_1* and *CASP9_5* had small peaks corresponding to secondary peaks in the experimental group, peak height was significantly lower than the threshold we set for classifying secondary peaks, i.e., 10% of all amplified primary peaks.

- Please admit the scraping algorithm had "insufficient accuracy" for identifying 5'–3' orientation from the text, necessitating de novo pair generation. Please briefly state how many pairs were lost or discarded due to this limitation.

We have added the following:

> “By matching directly to the Ref_seq databases, no primers were lost due missing metadata.”

- Fig-2B presents the percentage of papers using RT-qPCR in Methods, which does not indicate the uniqueness of the primers or primer sequences as mentioned in the text. (Line 66)

We apologize for including reference to the wrong figure panel. The text should have said Figure 2C, not 2B. We have updated it as such.

- Fig. 4B: hexbin could use colorbar scale.

The colorbar was originally to the right of the plot and labeled as “synergy score” with a range of 0 to ~0.9. However given the density of panels within the figure relative to its size, we believe that that point is easily missed. We have spread out the panels within the figure and highlighted Figure 4B with a dashed box inclusive of the colorbar to make the bar stand out more.

- UMAP in Fig. 3E: please label clusters more prominently.

Clusters have been numbered corresponding the group names at right in the figure panel.

- Fig. 5C/D: please add arrows to peaks.

This correction was made.

- References: please add recent reviews on RT-qPCR reproducibility (e.g., MIQE guidelines update).

We have added 6 references to the manuscript include those describing MIQE guidelines.

- Ethics: No human/animal subjects, but confirm IRB if IMR90 RNA from donors.

IMR90 lung fibroblasts (ATCC CCL-186) were commercially obtained from ATCC. Hence no institutional approvals were required.

- Typos/Grammar:

o Line 45, 52, 225, 356: punctuation should be after citation notes.

This correction was made.

o Line 64: "~31% of all oligo sequences found are unique" → "31% ... are unique".

This correction was made.

o Lines 133-134: capitalize the Genus in species' names.

This correction was made.

o Line 144: hamming distance ◊ Hamming distance

This correction was made.

o Line 149-150: "prime occurrences" → "primer".

This correction was made.

o Line 231: there is no Figure S3-33

This correction was made so that it now reads Figure S3-S33.

o Line 246: "melt curve optimization" → "melting".

This correction was made.

o Line 272-273: "31% of primers are still used only once" – consistent with Fig. 2C?

Yes the peak occurs at 31% for a single occurrence. However, by starting at an occurrence of 1 and having the x-axis start a 2, it obscured the fact that the distribution is a peak at 1 with a value of 31%. We have rescaled the distribution from 0 to 10 on the x-axis to illustrate this point.

o Line 424: "thirst highest" → "third".

This correction was made.

o Methods: Consistent units (e.g., "ug" → "µg").

This correction was made.

Reviewer #3 (Remarks to the Author):

I co-reviewed this manuscript with one of the reviewers who provided the listed reports. This is part of the Nature Communications initiative to facilitate training in peer review and to provide appropriate recognition for Early Career Researchers who co-review manuscripts.

We thank this reviewer for their time and hope that the comments above and manuscript changes have improved the paper in the reviewers' opinions.

We would like to thank the editors and reviewers at Nature Communications for careful consideration of the manuscript and for their comments. We have responded below to each of the concerns (in **BLACK**) on a point-by-point basis in **BLUE** and made text changes to the manuscript in **RED**.

Reviewer #2

The authors have addressed most key issues in this revision, including method validation, data analysis, comparisons to existing resources, and other methodological problems. However there are still some concerns that need to be fixed before the publication:

[Minor comments]

1. There should be clear discussion in the revised manuscript about the efficiency of picked primers over the others and the manual check/validation plan.

> We agree with this assessment. The following has been added to the discussion section of manuscript:
>
> > These 83 primer pairs represent 4.1% of our 2,017 primer pairs over a score of 80. Applying the Wilson Score Interval gives us a 95% confidence interval of a true amplification efficiency of all primers with PICKR scores >80 as 93.6 to 99.8%. Further validation will come from community tested sequences that cite our manuscript which will be highlighted on the database.

2. The answear for the this question is still not clear: “Additionally, the synergy score penalizes ΔTm >0°C (with a score 0 for >3°C) seems strictly, so please validate this against your data, as Fig. 4E shows weak negative correlation between ΔTm and evidence (~ -0.2-0.3?).” Also, there should be a clearer explanation about the scoring functions. It’s not still clear why all weights are chosen and what are their impacts.

> We thank the reviewer for this suggestion. The following discussion has been added to the manuscript:
>
> > Evidence thresholds of 25, 100, and 500 for matched papers, unmatched papers with the correct sequence, and unmatched papers with reverse complement sequences were chosen due to perceived increase in mistrust. All three biophysics sub-parameters were weighed equally as there was no evidence of one factor having more influence than another. … An increasing synergy penalty for forward and reverse primers with ΔTm > 0 °C (with 0 for ΔTm > 3°C) was implemented to increase the likelihood of top primer pairs amplifying the best around 60 °C. A synergy score of 0 was also applied for any primer pairs with complementary > 5 to limit off target amplifications.

3. There should be a clear discussion in revised manuscript about this: “The authors mention future iterations using OCR for locked PDFs; providing a roadmap or preliminary data on how much more "dark data" could be recovered would be valuable for the Nature Communications audience.”

> We thank the reviewer for this suggestion. The following discussion has been added to the manuscript:

Given an average of 18 nucleotides per paper and ~220,000 papers mapped as potential candidates that we failed to extract sequences from, we may have missed ~3-4 million unique oligos, equating to 30% of our database. This could enable our gene counts to reach 10,000. Future text-mining iterations will incorporate optical character recognition for locked PDFs to further close this gap. This will include the use of Mistral OCR 3 and LLM integration.

4. Did the author Jun K. Robbins join during this revision?

Yes, and the editors have been informed of his new role in the manuscript. As noted in the prior revision, Jun contributed as noted on lines 547-8:

"…Al.B. and J.K.R. designed and executed all RT–qPCR validation experiments and generated the benchmark data set."

5. The code URL can't be reached: https://zenodo.org/doi/10.5281/zenodo.17058652

We have refreshed the uploaded code to help ensure accessibility.
